# EphrinB1/EphB3b Coordinate Bidirectional Epithelial-Mesenchymal Interactions Controlling Liver Morphogenesis and Laterality

**DOI:** 10.1016/j.devcel.2016.10.009

**Published:** 2016-11-07

**Authors:** Jordi Cayuso, Aliaksandr Dzementsei, Johanna C. Fischer, Gopal Karemore, Sara Caviglia, Josefin Bartholdson, Gavin J. Wright, Elke A. Ober

**Affiliations:** 1 Division of Developmental Biology, Mill Hill Laboratories, The Francis Crick Institute, London NW7 1AA, UK; 2 Danish Stem Cell Center (DanStem), University of Copenhagen, 2200 Copenhagen N, Denmark; 3 Novo Nordisk Foundation Center for Protein Research, Protein Imaging Platform, University of Copenhagen, 2200 Copenhagen N, Denmark; 4 Wellcome Trust Sanger Institute, Cell Surface Signalling Laboratory, Cambridge CB10 1HH, UK

**Keywords:** liver, morphogenesis, migration, repulsion, bidirectional, protrusion, EphrinB1, EphB3, lateral plate mesoderm, zebrafish

## Abstract

Positioning organs in the body often requires the movement of multiple tissues, yet the molecular and cellular mechanisms coordinating such movements are largely unknown. Here, we show that bidirectional signaling between EphrinB1 and EphB3b coordinates the movements of the hepatic endoderm and adjacent lateral plate mesoderm (LPM), resulting in asymmetric positioning of the zebrafish liver. EphrinB1 in hepatoblasts regulates directional migration and mediates interactions with the LPM, where EphB3b controls polarity and movement of the LPM. EphB3b in the LPM concomitantly repels hepatoblasts to move leftward into the liver bud. Cellular protrusions controlled by Eph/Ephrin signaling mediate hepatoblast motility and long-distance cell-cell contacts with the LPM beyond immediate tissue interfaces. Mechanistically, intracellular EphrinB1 domains mediate EphB3b-independent hepatoblast extension formation, while EpB3b interactions cause their destabilization. We propose that bidirectional short- and long-distance cell interactions between epithelial and mesenchyme-like tissues coordinate liver bud formation and laterality via cell repulsion.

## Highlights

• Cell-shape changes and oriented hepatoblast migration mediate asymmetric liver budding
• Bidirectional EphrinB1/EphB3b signaling coordinates liver and LPM movements
• Hepatoblast and LPM protrusions include long-distance connections to each other
• EphB3b-dependent and -independent EphrinB1 functions regulate hepatoblast migration

## Graphical Abstract

**Figure:**
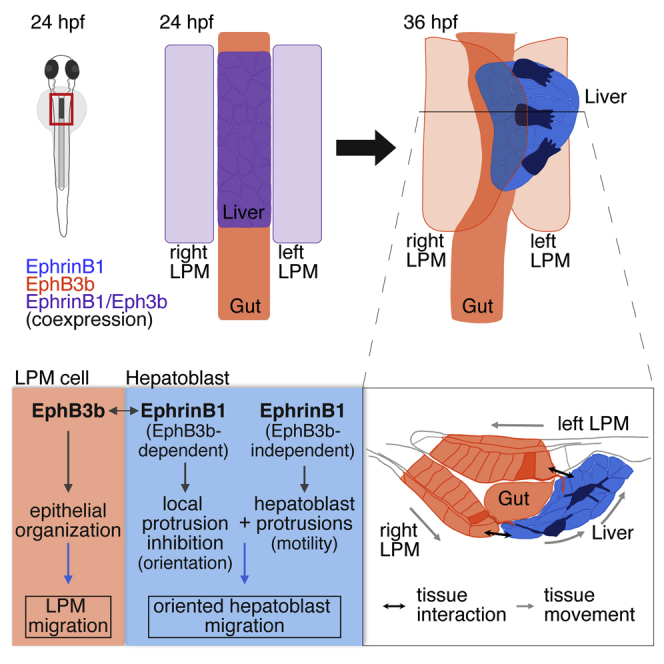

## Teaser

The mechanisms controlling the asymmetric positioning of visceral organs are largely unknown. Cayuso et al. show that bidirectional EphrinB1/EphB3b signaling during liver positioning in zebrafish coordinates active directional hepatoblast migration with the movements of the adjacent lateral plate mesoderm. Long-distance cell-cell interaction between hepatoblasts and mesoderm is mediated via filopodia.

## Introduction

Complex cell rearrangements are a fundamental feature of embryonic development, converting patterning information into organs and embryos of distinct shapes, sizes, and organization. Great progress has been made in unraveling how single cells and groups of cells move, whereas it is largely unknown how the movement of multiple tissues is coordinated. In the digestive system, the progenitors of the foregut and its accessory organs, the lungs, liver, and pancreas, are specified from a pool of foregut endoderm cells (Zorn and Wells, 2007). These progenitor populations rearrange to form organ buds in stereotypic positions along the alimentary canal, establishing the foundation of the adult organs. Asymmetric positioning of the majority of the visceral organs, including the liver and pancreas is required for their compact packing within the abdominal cavity. Seminal studies in mouse and zebrafish suggest that migration or asymmetric cell rearrangement of adjacent mesodermal tissues have essential roles in the left-right placement of the endodermal organ progenitors (Davis et al., 2008, Horne-Badovinac, 2003). This highlights a fundamental question in development: How are complex morphogenetic movements of multiple tissues coordinated at the cellular and the molecular level?

**Figure 1:**
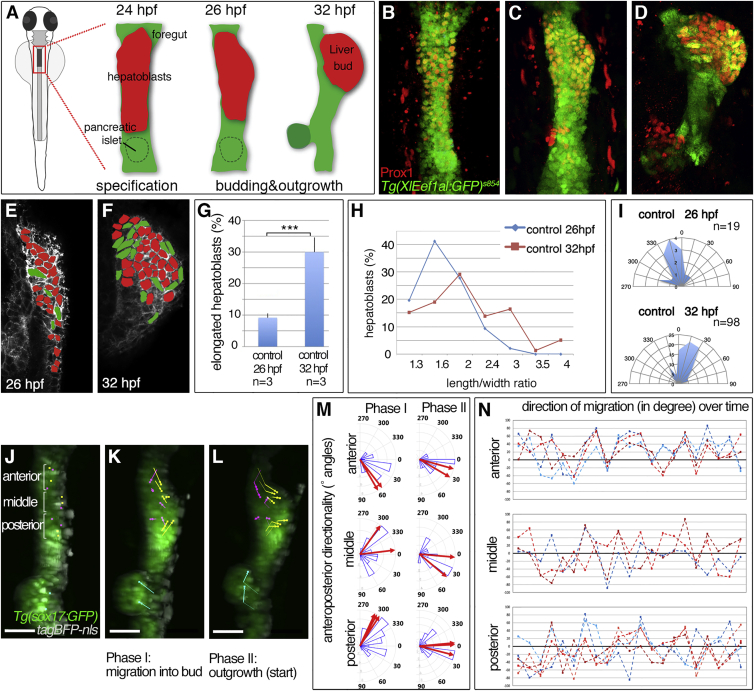
Hepatoblast Polarization Coincides with Liver Budding (A–D) Stages of liver budding: Schematic (A) and confocal projections of corresponding stages with *Tg(XlEef1a1:GFP)*^*s854*^ marking the endoderm and Prox1 hepatoblasts; ventral views (B–D). (E and F) EphrinB1 staining highlights cell shapes at the start of budding (E) and when a bud is apparent (F). Morphometric measurements were performed on serial coronal sections of the bud (E and F); elongated hepatoblasts (L/W ≥ 2) are shown in green. (G–I) Quantification of hepatoblast shape in control embryos at 26 and 32 hpf: (G) proportion of elongated cells per bud; SEs are shown, (H) L/W distribution for one representative bud; (I) orientation of elongated hepatoblasts with respect to the anteroposterior axis. (J–L) Time lapse of *Tg(sox17:GFP)*-positive foregut starting around 25 hpf (J) shows distinct hepatoblast movements during liver budding (K) and onset of outgrowth (L); dorsal views. TagBFP-nls (gray) marks nuclei for tracking of liver (yellow), gut (magenta), and pancreas progenitors (cyan). (M and N) Hepatoblasts from different anteroposterior positions migrate with distinct orientation. (M) Rose plots show the distribution of angular displacement with respect to the embryonic midline for 28 min intervals (blue sectors) and the angle of mean displacement per cell for the entire period (red arrow). (N) Line plots representing directionality of displacement over time show individual angular cell displacement for various liver (red hues) and gut progenitors (blue hues). Scale bars represent 40 μm. ^∗∗∗^p < 0.001. See also Figure S1; Movies S1, S2, and S4.

In zebrafish, the liver progenitors, or hepatoblasts, are specified in the ventral foregut by signals secreted from the adjacent lateral plate mesoderm (LPM) (Chung et al., 2008, Ober et al., 2006, Poulain and Ober, 2011, Shin et al., 2012). Hepatoblasts are initially located symmetrically at the embryonic midline and form shortly after specification an organ bud left of the midline (Field et al., 2003) (Figures 1A–1D). Several transcriptional regulators expressed in hepatoblasts, including Hhex and Prox1, have been associated with different aspects of early liver outgrowth, such as cell proliferation, adhesion, and basal lamina remodeling (Bort et al., 2006, Lüdtke et al., 2009, Margagliotti et al., 2007, Sosa-Pineda et al., 2000, Wallace et al., 2001). To date, there is little evidence for active hepatoblast movements in liver budding (Bort et al., 2006, Klein et al., 2011), whereas the movement of the bilateral LPM epithelia adjacent to the foregut has been shown to be crucial for leftward hepatoblast positioning (Horne-Badovinac, 2003). Concomitant with leftward gut looping and liver positioning, the left LPM moves dorsal to the endoderm, while the right LPM moves ventrolaterally toward the endoderm (Figures 4A‴–4B‴). Mutants with disrupted LPM epithelial morphology or impaired ECM degradation show defective LPM movement and midline-positioned gut and liver, which led to the model that active LPM movements, in particular of the right LPM, exert a motive force on the passive endodermal progenitors directing leftward gut looping and liver outgrowth (Hochgreb-Hagele et al., 2013, Horne-Badovinac, 2003, Yin et al., 2010). How exactly the mesoderm controls this complex morphogenetic rearrangement of the liver progenitors into the liver bud is unclear.

Eph receptor tyrosine kinases and their membrane-tethered Ephrin ligands are divided into two classes: A-type GPI-linked Ephrin ligands interact primarily with EphA receptor tyrosine kinases, and conversely B-type transmembrane EphrinB ligands interact predominantly with EphB receptors (Kania and Klein, 2016). A unique property of Eph/Ephrin interactions is the bidirectional activation of signaling. The *trans*-interaction of Ephrin and Eph from adjacent cells initiates forward signaling in Eph-expressing cells and reverse signaling downstream of Ephrins. However, ligand and receptor expression in the same cell can result in *cis*-interactions that interfere with forward and reverse signaling (Yaron and Sprinzak, 2012). Eph/Ephrin signaling regulates a great variety of cell behaviors, including cell adhesion, shape changes, and migration, important for diverse morphogenetic processes during embryonic development and tissue homeostasis (Kania and Klein, 2016, Pasquale, 2005). Therefore, members of the Eph and Ephrin families represent attractive candidates for controlling the morphogenetic events driving leftward liver outgrowth. Intriguingly, hepatic *EphrinB1* expression has been observed in several vertebrates (Costa et al., 2003, Fletcher et al., 1994, Thisse and Thisse, 2005), whereas its function and an interacting Eph receptor in this context are unknown.

Here, we show that bidirectionally coordinated endoderm and mesoderm movements are crucial for liver bud morphogenesis within the embryo. Contrary to previous models, we show that active hepatoblast migration is essential for liver bud formation and positioning. We identify EphrinB1 and the receptor EphB3b as key factors coordinating the interlinked morphogenetic behaviors of the hepatic endoderm and adjacent LPM, essential for directional liver outgrowth. Mechanistically, we show that EphB3b-independent EphrinB1 function controls hepatoblast protrusion formation, while asymmetric expression of EphB3b in the right LPM triggers EphrinB1-mediated repulsive activity that provides instructive directional cues for mediating asymmetric liver morphogenesis.

## Results

### Hepatoblasts Actively Migrate during Liver Budding

To determine whether hepatoblasts rearrange actively or are passively displaced during liver budding, we examined their cell behaviors by first assessing cell shapes. Hepatoblasts were outlined by immunolabeling against the transmembrane protein EphrinB1 (Figures 1E and 1F; EphrinB1 expression is described in detail later). We determined the length/width (L/W) ratio of hepatoblasts in coronal and transverse sections at two time points: at 26 hr post fertilization (hpf), the onset of budding when the first hepatoblasts are found left of the midline; and at 32 hpf, when an organ bud has formed and outgrowth is still ongoing (Field et al., 2003). These analyses revealed significant cell-shape changes over time: in coronal sections only 9.2% of all hepatoblasts were elongated (L/W ≥ 2) at 26 hpf, while at 32 hpf this population increased dramatically, comprising 30% (Figures 1E–1G). Concurrently, the overall hepatoblast L/W ratio increases significantly (Figure 1H). During budding, elongated cells were predominantly oriented in a 0°–30° angle with respect to the anteroposterior axis (Figure 1I), consistent with directional, anterior-leftward hepatoblast outgrowth. Cell-shape analysis in transverse sections (encompassing dorsoventral and mediolateral axes) revealed no difference in hepatoblast elongation at 26 and 32 hpf (Figure S1), suggesting cell polarization along the anteroposterior axis. In contrast to previous models, in which gut looping and liver positioning are solely the result of asymmetric LPM migration and passive hepatoblast displacement (Hochgreb-Hagele et al., 2013, Horne-Badovinac, 2003, Yin et al., 2010), these shape changes indicate oriented hepatoblast movement during budding.

To further validate our hypothesis, we followed hepatoblast movement in the embryo using time-lapse confocal microscopy during budding, between 24 and 36 hpf. Tracking of fluorescently labeled nuclei revealed that hepatoblasts move in a coordinated fashion and neighbor exchange occurs between hepatoblasts, suggesting active collective cell migration (Figures 1J–1N and S1F–S1H, Movies S1 and S2). First, hepatoblasts move directionally to aggregate into the liver bud, with anterior hepatoblasts moving posterior leftward, posterior ones moving anterior-leftward, and the intermediate population just leftward (Figures 1K and 1M). This is followed by a second phase where hepatoblasts move more directionally to the left with a more consistent angular displacement (Figures 1L and 1M). In contrast, other endodermal populations exhibit distinct motile behaviors, such as future gut cells, which initially reside at the same anteroposterior position of the endodermal rod, but undergo smaller displacements with clear differences in the direction of movement (Figures S1I and S1J, Movies S1 and S2) and pancreatic cells which move in the opposite direction, anterior-right (Figures 1J–1M). Although hepatoblasts from the same area migrate in the same overall direction, individual cells display different directionality relative to each other at a given time point, corroborating active migration (Figure 1N). Together, these data show that hepatoblasts actively migrate during liver budding.

### Hepatoblasts Form Lamellipodia- and Filopodia-like Protrusions

**Figure 2:**
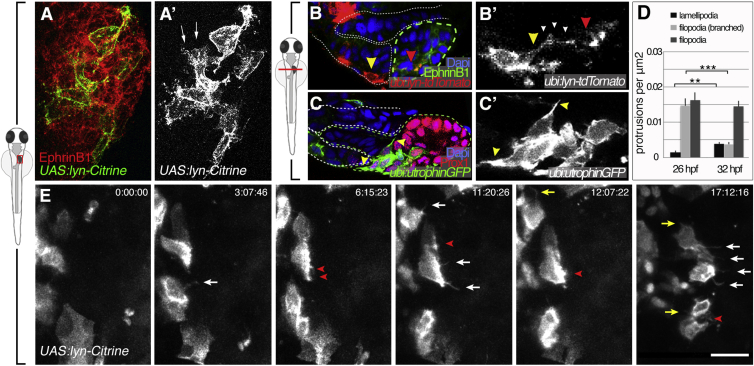
Hepatoblasts Form Filopodia- and Lamellipodia-like Protrusions during Liver Budding (A–B′) Mosaic *UAS:lyn-citrine* or *ubi:lyn-tdTomato* expression shows EphrinB1^+^ hepatoblasts form lamellipodia (arrows in A′) and filopodia-like extensions (B and B′). Extensions (white arrowheads) connect the LPM (yellow arrowhead) and hepatoblasts (red arrowhead) over several cell diameters (B and B′). Dashed lines outline the LPM (white) and endoderm (green). (C and C′) Utrophin-GFP highlights actin in the cortical network and protrusions of hepatoblasts (arrowheads). Dashed lines delineate endoderm (green) and LPM (white). (D) Quantification of hepatoblast protrusions shows an increase of lamellipodia and decrease of filopodia-like extensions during budding. (E) Time lapse of migrating hepatoblasts during liver budding and early outgrowth; dorsal views. Membrane labeling with *UAS:lyn-Citrine* shows filopodia in the direction of outgrowth (white arrows) and toward the LPM (yellow arrows) and lamellipodia (red arrowheads); stills of Movie S3. Scale bars represent 30 μm. ^∗∗^p < 0.01, ^∗∗∗^p < 0.001). See also Movie S3.

Our cell-shape and time-lapse analyses suggest that hepatoblasts actively move during liver budding. To corroborate this finding, we examined hepatoblast morphology at greater resolution by expressing membrane-tethered fluorescent proteins in small clones in the forming liver. This analysis revealed that wild-type hepatoblasts as well as LPM cells exhibit unexpectedly elaborate morphologies, including distinct cellular protrusions (Figure 2). We identified two major protrusion types: flat, sheet-like protrusions resembling lamellipodia (Figures 2A and 2A′), and thin filopodia-like extensions, a subset of which is branched (Figures 2B and 2B′). Filopodia and lamellipodia are F-actin-rich structures (Ridley, 2011). To examine the distribution of cellular actin in clones, we expressed GFP-tagged Utrophin, a protein associating with F-actin without interfering with its function (Burkel et al., 2007). Labeled cells showed an enrichment of GFP close to the cell membrane corresponding with cortical actin, as well as in hepatoblast and LPM protrusions (Figures 2C and 2C′), supporting their classification as filopodia- and lamellipodia-like extensions.

Hepatoblasts form filopodia-like extensions that are on average 3.4 μm long, and can reach up to 13.6 μm (equivalent to ∼2 cell diameters). Similarly, epithelial LPM cells form basal protrusions, which are on average 7.2 μm and up to 26.5 μm long. These extensions interconnect both tissues, as they frequently extend from LPM clones into the hepatic domain making contacts with hepatoblasts away from the tissue border and from hepatoblasts to the border of the LPM (Figures 2B and 2B′). These findings indicate that both tissues form direct physical contacts not only at the hepatoblast/LPM interface, but also long-distance cell-cell interactions.

Each protrusion type contributes to complex cellular behaviors, with filopodia exploring and sensing the environment and lamellipodia mediating movement (Ridley, 2011). To elucidate hepatoblast behaviors during budding, protrusions were quantified between 26 and 32 hpf. Hepatoblasts form about the same number of simple filopodia-like protrusions at both stages, while the number of branched filopodia-like protrusions decreases by 75% and lamellipodia formation dramatically increases by 163% at 32 hpf (Figure 2D), indicating a significant shift from predominantly sensing to more motile cell behaviors during budding. This protrusive activity was corroborated by live imaging, showing filopodia and lamellipodia-like protrusions dynamically extending in the direction of migration, as well as some toward the midline and LPM (Figure 2E and Movie S3).

### Cell Protrusions Are Important for Hepatoblast Positioning

**Figure 3:**
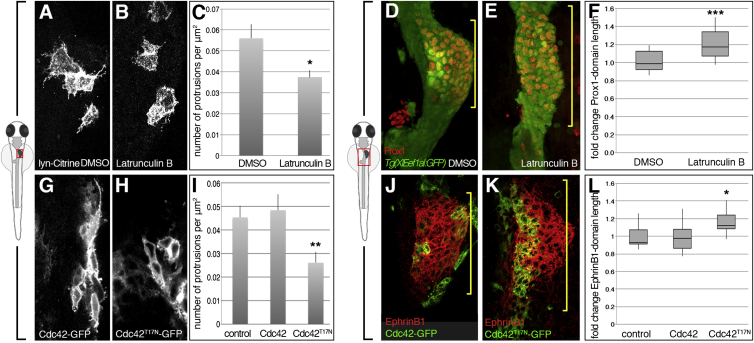
Compromised Protrusion Formation Correlates with Liver Budding Defects (A–F) Latrunculin B (0.1 μg/ml) treatment during liver budding (26–32 hpf) leads to significantly less hepatoblast protrusions (A–C; DMSO clones, n = 7; Lat B clones, n = 9, N = 1), and a significantly longer Prox1 domain (bracket) (D–F; DMSO, n = 23; Lat B, n = 13, N = 2). (G–L) Hepatoblasts expressing Cdc42^T17N^-GFP during liver budding (26–32 hpf) form significantly less protrusions compared to controls and Cdc42-GFP (G–I; control clones, n = 10; Cdc42-GFP clones, n = 16; Cdc42^T17N^-GFP clones, n = 18, N = 1) and a significantly longer EphrinB1 domain (bracket) (J–L; control, n = 7; Cdc42-GFP, n = 18; Cdc42^T17N^-GFP, n = 16, N = 2). N indicates the number of experiments. ^∗^p < 0.05, ^∗∗^p < 0.01, ^∗∗∗^p < 0.001.

To investigate the functional relevance of these cell extensions in liver bud morphogenesis, *Tg(XlEef1a1:GFP)*^*s854*^ embryos with GFP highlighting the endoderm were incubated during early budding stages with the F-actin-depolymerizing drug Latrunculin B (Lat B). Given the importance of actin polymerization for numerous cellular processes, we minimized the exposure to Lat B and treated the embryos from 26–32 hpf with a low dose of the drug (0.1 μg/ml). This treatment resulted in a 33% decrease in the number of hepatoblast protrusions at 32 hpf (Figures 3A–3C), and ectopic Prox1-positive hepatoblasts in posterior positions leading to a 20% increase of the anteroposterior extent of the Prox1 domain compared with DMSO-treated controls (Figures 3D–3F). In addition, hepatoblasts resided at or close to the midline, suggesting that oriented anterior-leftward hepatoblast migration is compromised.

To determine the cell autonomous functions of protrusions in liver budding, we manipulated the small Rho GTPase Cdc42, a well-known regulator of filopodia formation (Nobes and Hall, 1995). Mosaic expression of the dominant-negative Cdc42^T17N^ (Nalbant et al., 2004) at 26 hpf resulted in a significant reduction of hepatoblast protrusions at 32 hpf (Figures 3G–3I), which is accompanied by a significant increase of the anteroposterior extent of the Prox1 domain (Figures 3J and 3K). In contrast, wild-type Cdc42 overexpression alters neither protrusion number (Figures 3I and 3K) nor the length of the Prox1 domain.

Altogether, these findings support the importance of cellular extensions for hepatoblast movement in liver budding.

### EphrinB1 and EphB3b Expression Is Dynamic and Complementary in the Liver-Forming Foregut Domain

**Figure 4:**
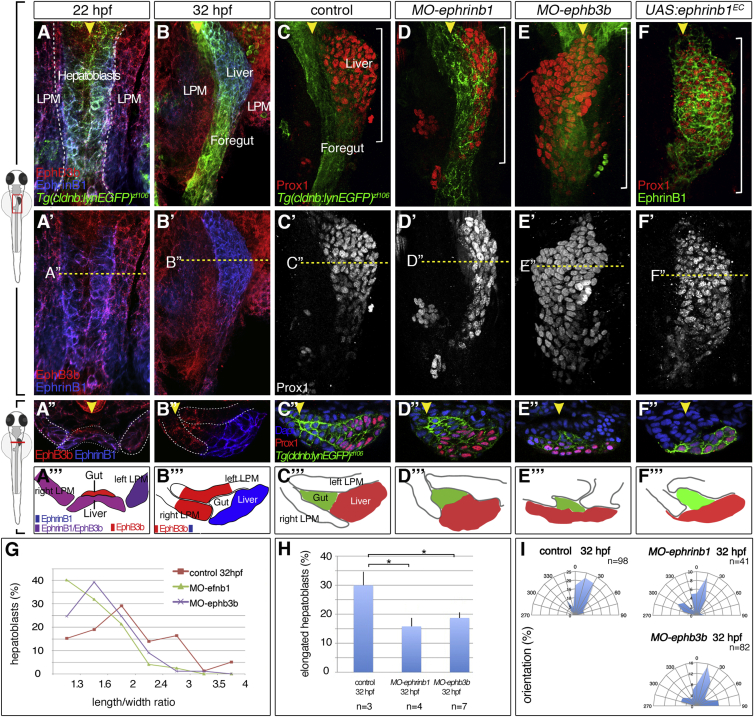
Complementary EphrinB1 and EphB3b Expression Controls Hepatoblast Positioning and Cell-Shape Changes in Liver Bud Formation (A–F‴) At 22 hpf, EphrinB1 and EphB3b expression largely overlaps in future hepatoblasts and the LPM (A–A‴). Complementary expression of both factors coincides with the start of liver budding: EphrinB1 in hepatoblasts and EphB3b in the gut and LPM (B–B‴). At 32 hpf, hepatoblasts are located more posteriorly and medially compared with controls in *MO-ephrinb1* (C–D‴), in *MO-ephb3b* (E–E‴), and upon conditional *UAS:ephrinb1*^*EC*^ expression (F–F‴). (A–F′) ventral views of confocal projections, anterior to the top; (A″–F″) transverse sections of the foregut, as indicated by the dashed line in (A′–F′), and matching schematics (A‴–F‴); yellow arrowheads specify the midline and white brackets the length of the Prox1 domain. (G–I) Cell shapes were determined with EphrinB1-staining at 32 hpf (see Figure 1F). Quantification of hepatoblast shape in control, *MO-ephrinb1*, and *MO-ephb3b* embryos: (G) L/W distribution for one representative bud; (H) proportion of elongated cells per bud; SEs are shown; and (I) orientation of elongated hepatoblasts with respect to the anteroposterior axis. ^∗^p < 0.05. See also Figure S2.

Searching for factors that mediate hepatoblast movement, we identified *ephrinb1* expression in the liver domain during budding stages (Figure S2C). EphrinB1 represented an excellent candidate, since Ephrin ligands and their Eph receptors can control cytoskeletal dynamics and thereby diverse morphogenetic processes (Kania and Klein, 2016). Given that EphrinB1 signaling is generally activated by interaction with EphB receptors, we searched for one expressed in the foregut area and identified the EphB3 homolog *ephb3b* (Figure S2D). In order to examine EphrinB1 and EphB3b expression at cellular resolution, we generated antibodies against their ectodomains that recapitulate the corresponding mRNA expression (Figures 4A–4B‴ and S2A–S2D). We found that EphrinB1 represents one of the first genes expressed in zebrafish liver precursors, starting around 22 hpf, which corresponds with the onset of previously described hepatoblast gene expression (Ober et al., 2006). At this stage, EphB3b is co-expressed with EphrinB1 in the hepatic endoderm and adjacent LPM, while, in the gut, solely EphB3b is detected (Figures 4A–4A‴). From 26 hpf, with the onset of liver budding, ligand and receptor expression become complementary: EphrinB1 is expressed in hepatoblasts, while EphB3b is present in the LPM and restricted gut domains anterior and posterior to the liver anlage (Figures 4B–4B‴). Altogether, EphrinB1 and EphB3b interaction interfaces are established between hepatoblasts and the adjacent LPM during liver budding.

### EphrinB1 and EphB3b Control Early Liver Bud Morphogenesis

To determine the role of EphrinB1 and EphB3b in liver organogenesis, and the functional significance of the hepatoblast-LPM interactions, we used morpholino antisense oligonucleotide (MO)-mediated knockdown (Figures S2E–S2J′). In *ephrinB1* or *ephB3b* morphant embryos, liver specification is indistinguishable from controls at 25 hpf (Figures S2K–S2N), whereas hepatoblast positioning and bud formation are defective in 88% of *ephrinB1* morphants (n = 140) and 86% of *ephB3b* morphants (n = 211) at 32 hpf (Figures 4C–4E and S2X–S2Z). Similar defects were observed upon transgenic expression of EphrinB1^EC^, a dominant-negative form encompassing the extracellular domain of EphrinB1 (Holder and Klein, 1999), confirming a requirement for EphrinB/EphB signaling in liver morphogenesis (Figures 4F–4F‴). In embryos with impaired EphrinB1 or EphB3b function, ectopic Prox1-positive hepatoblasts reside in more posterior and medial positions (Figures 4D–4F″ and S2X–S2Z), causing subsequent liver morphology defects at 54 hpf, with altered extrahepatic duct formation and liver tissue located closer to or ectopically at the midline (Figures S2O–S2W′). In morphant embryos, liver budding is more severely disrupted by EphB3b knockdown, with frequently bilaterally located hepatoblasts (Figures 4E–4E‴ and S2V–S2V′). To test whether the difference in phenotype might be due to incomplete EphrinB1 knockdown, we generated a genetic mutant using the Crispr/Cas9 system. e*phrinb1*^*nim26*^ mutants exhibit ectopic Prox1-positive hepatoblasts with 90% showing an elongated Prox1 domain at 32 hpf (n = 16; Figures S2Bb–S2Bb″). *ephrinb1* mutant and morphant embryos therefore show a similar phenotype and, unlike in EphB3b morphants, hepatoblasts are generally positioned to the left and not across the midline. This indicates that the EphrinB1 and EphB3b knockdown phenotypes are specific and not due to incomplete knockdown of EphrinB1. Moreover, the number of Prox1-positive cells is not altered in *ephrinB1* and *ephB3b* morphants (Figure S2Aa), indicating that the domain expansion is due to morphogenesis defects and not increased proliferation or ectopically specified progenitors.

### EphrinB1 and EphB3b Regulate Hepatoblast Cell-Shape Changes and Epithelial LPM Organization

Ephrins/Ephs are important regulators of tissue morphogenesis, including polarization and oriented migration of cells (Pasquale, 2005, Poliakov et al., 2004). To ascertain whether EphrinB1 and EphB3b regulate liver morphogenesis by controlling cell polarity and migration during budding, we analyzed hepatoblast morphologies at 32 hpf. Coronal sections showed 47% and 37% fewer elongated hepatoblasts in *ephrinB1* or *ephb3b* morphants, respectively, displaying significantly reduced L/W ratios and altered orientations with respect to the midline (Figures 4G–4I). These findings suggest that EphrinB1 and EphB3b regulate hepatoblast rearrangement during budding.

**Figure 5:**
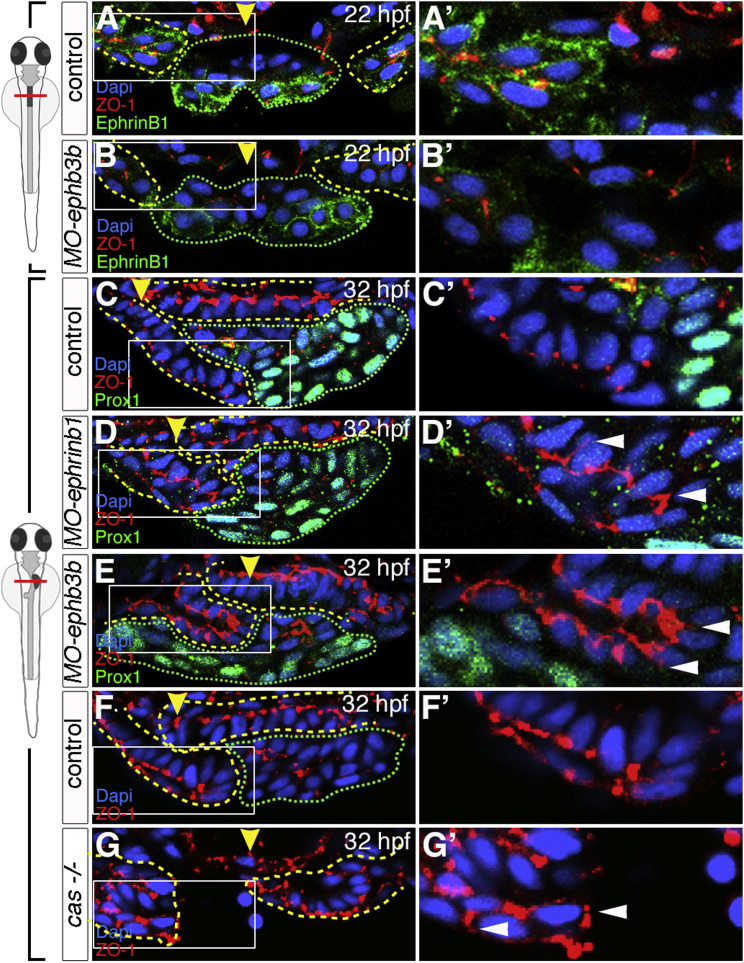
EphB3b Controls LPM Polarity and Asymmetric Movement (A–E′) EphrinB1 and EphB3b regulate LPM polarity in the foregut region. α-ZO-1 staining in *MO-ephb3b* embryos reveals that junctions in the LPM form similar to controls at 22 hpf (A–B′); while at 32 hpf, ZO-1 is mislocalized (arrowheads) in *MO-ephrinb1* and *MO-ephb3b* embryos (C–E′). Yellow arrowheads specify the midline and reveal impaired gut looping in *MO-ephrinb1* and *MO-ephb3b* (C–E). (F–G′) *casanova* mutants exhibit ZO-1 localization defects in the LPM (arrowheads) at 32 hpf. (A–G′) Transverse sections at liver level, left side to the right; dashed lines delineate the endoderm (green) and LPM (yellow); (A′–G′) are magnifications of the areas indicated by a box in (A–G).

Since EphrinBs and EphBs represent bidirectional signaling pairs, we analyzed the morphology of the EphB3b-expressing LPM and its behaviors. Polarization of the LPM epithelia is key for asymmetric LPM migration, which directs leftward gut looping and liver positioning (Horne-Badovinac, 2003). In *ephrinb1* and *ephb3b* morphants, gut looping was impaired, with more pronounced defects in the latter (Figures 4D–4E‴ and 5C–5E′). Consistent with more severe defects in *ephb3b* morphants, medially positioned guts were accompanied by symmetrically placed left and right LPM, mostly dorsal to the endoderm (Figures 4E″, 5E, and 5E′). To visualize LPM cell polarity in morphants, we analyzed Zonula occludens1 (ZO-1) localization marking tight junctions. In *ephrinb1*-and *ephb3b*-morphant LPMs, tight junctions were established initially at 22 hpf (Figures 5A–5B′), however, their localization was subsequently disrupted and apical ZO-1 frequently expanded basally at 32 hpf (Figures 5C–5E′). Hence, both EphrinB1 and EphB3b are required to maintain LPM epithelial organization and associated asymmetric LPM movement, likely through activation of forward signaling downstream of EphB3b in the LPM.

These findings also implicate that signals from the endoderm to the mesoderm are important for mesoderm morphogenesis. To further explore the role of the endoderm in this process, we performed ZO-1 stainings in endoderm-less *casanova/sox32* mutants. This revealed severe defects in LPM polarity (Figures 5F–5G′), confirming the crucial function of the endoderm and derived signals in LPM organization.

### EphrinB1 Controls Hepatoblast Protrusions by EphB3b-Independent and -Dependent Functions

**Figure 6:**
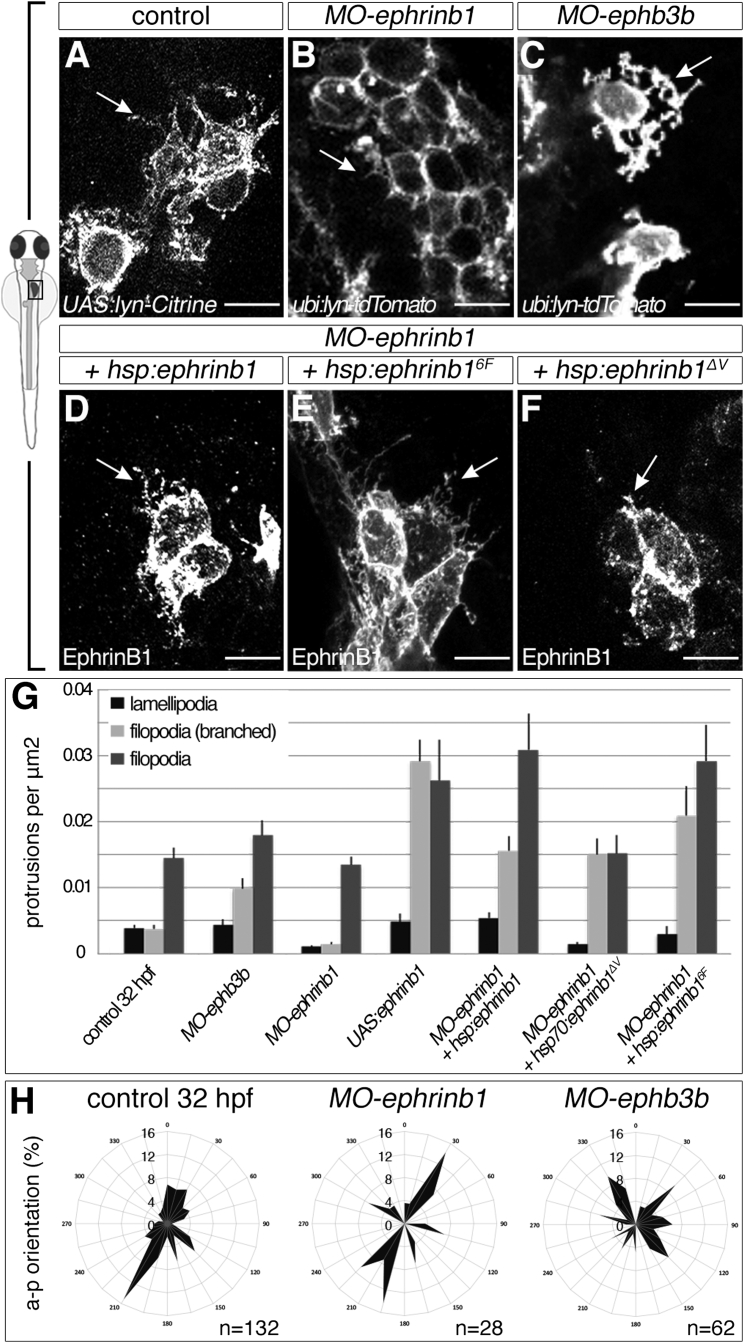
Opposing EphrinB1 and EphB3b Functions Control Formation of Hepatoblast Protrusions (A–G) EphrinB1 and EphB3b regulate hepatoblast protrusion number and morphology. Sparse labeling reveals fewer and shorter filopodia in *MO-ephrinb1* hepatoblasts than in controls (A and B). Conversely, *MO-ephb3b* hepatoblasts show more complex, branched extensions (C). In contrast to EphrinB1 and EphrinB1^6F^ (D and E), EphrinB1^ΔV^ fails to rescue extension formation in *MO-ephrinb1* (F). Arrows indicate representative protrusions (A–F). Quantification of hepatoblast protrusion types in various conditions; comparative p values are shown in Table S1 (G). (H) Hepatoblast lamellipodia orientation is randomized in *MO-ephb3b*. (A–C) lyn-Citrine and lyn-Tomato outline hepatoblasts, (D–F) EphrinB1 staining highlights overexpression of different forms of EphrinB1, (A–F) ventral projections, anterior to the top. SEs are shown. Scale bars represent 10 μm. See also Figure S3; Table S1.

Given that EphrinB1 and EphB3b control cell-shape changes and polarity during liver bud morphogenesis, and the similarity of the *MO-ephrinb1* and *MO-ephb3b* liver budding phenotypes to those following Lat B treatment, we decided to examine the role of EphrinB1 and EphB3b in hepatoblast protrusion formation. Using the sparse labeling strategy to visualize cell membranes, we observed that extension formation was generally impaired in *ephrinB1* morphants, resulting in fewer and shorter protrusions (Figures 6A, 6B, and 6G), whereas EphrinB1 overexpression significantly increases protrusion formation (Figures 6D, 6G, and S3A–S3B″, Table S1). In contrast, knockdown of EphB3b resulted in an unexpected and striking increase in branched filopodia-like protrusions (Figures 6A, 6C, and 6G), indicating that both factors seem to have opposite effects on protrusion formation. Furthermore, when examining the direction of these protrusions with respect to the anteroposterior axis, we found that lamellipodia in control or *MO-ephrinb1* embryos are preferentially oriented in the direction of hepatoblast movement and bud outgrowth, while lamellipodia orientation in *MO-ephb3b* embryos is randomized (Figure 6H). This indicates that EphrinB1 is required and sufficient to promote protrusion formation in hepatoblasts. Interaction with EphB3b expressed in LPM cells destabilizes filopodia and orients lamellipodia, thus providing spatial information. Hence, *MO-ephrinb1* hepatoblasts exhibit impaired motility, while this is intact in *ephb3b* morphant hepatoblasts, which instead, due to the lack of repulsive directional cues, distribute across the midline, explaining the common and distinct hepatoblast positioning phenotypes in either knockdown. These results also imply that EphrinB1 functions both dependently and independently of EphB3b in hepatoblasts.

Receptor-dependent and -independent EphrinB1 functions are mediated by conserved signaling motifs in the cytoplasmic domain, including six tyrosine phosphorylation sites and a C-terminal PSD-95/discs large/ZO-1 (PDZ)-interacting domain (Bochenek et al., 2010, Cowan and Henkemeyer, 2002). To distinguish domain-specific activities in hepatoblasts, we conditionally expressed full-length EphrinB1 or EphrinB1 mutant proteins in *MO-ephrinb1* hepatoblasts and examined hepatoblast protrusions. Mosaic expression of EphrinB1 or EphrinB1^6F^, in which phosphotyrosine signaling is impaired, rescues the formation of all protrusion types during liver budding (Figures 6D, 6E, and 6G). In contrast, expression of EphrinB1^ΔV^, which is unable to interact with PDZ-domain proteins (Davy et al., 2004), failed to restore lamellipodia as well as basic filopodia formation (Figures 6F and 6G, Table S1). Notably, protrusion branching is increased upon expression of the different EphrinB1 proteins, similar to *ephb3b* morphants (Figure 6C and Table S1). This may reflect increased receptor internalization, due to possible saturation with ligands (Bush and Soriano, 2010), and is consistent with compromising oriented cell motility (Figure S3C). In summary, our results suggest that EphrinB1 promotes extension formation through its PDZ-binding domain, independent of EphB3b, while, upon receptor interaction, filopodia-like protrusions collapse and lamellipodia re-orient in line with repulsive receptor functions.

**Figure 7:**
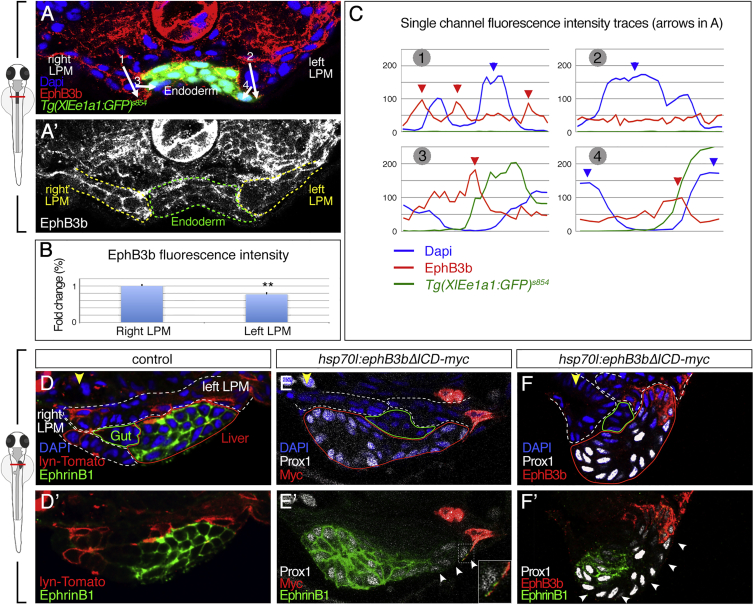
Asymmetric EphB3b Can Exert Repulsive Activity during Liver Budding (A–C) EphB3b expression is higher on the right than the left LPM at 24 hpf (A and A′). Quantification of EphB3b expression measuring overall fluorescent intensity (B) and intensity profile (C). Fluorescent intensity profiles show high EphB3b at cell membranes (red arrowheads) of the right but not left LPM; numbered arrows in (A) indicate the position of profiles. The right LPM-hepatoblast interface (profile 3) shows highest EphB3b expression. High DAPI levels indicate nuclei position (blue arrowheads). (D–F) Ectopic EphB3b^ΔICD^ expression alters hepatoblast position. Compared with mosaic lyn-Tomato (D), mosaic EphB3b^ΔICD^ expression on the left LPM (E) or left LPM and hepatoblasts (F) at 26 hpf causes positioning of Prox1^+^ hepatoblasts away from the clone at 32 hpf. EphrinB1 is absent from membranes in 3–7 hepatoblasts next to EphB3b^ΔICD^ clones (white arrowheads), without altering Prox1 expression (E′ and F′). (E′) Inset shows an EphrinB1 signal in EphB3b^ΔICD^ protrusion, indicating direct cell interaction. (A, A′ and D–F′) Transverse sections at liver level, left side to the right; yellow arrowhead specifies the midline; lines delineate the left and right LPM (white), gut (green), hepatoblasts (red). ^∗∗^p < 0.01. See also Figures S4 and S5.

A repulsion-based mechanism for leftward liver positioning would call for the asymmetric distribution of repulsive EphB3b to mediate leftward hepatoblast movement. Quantification of EphB3b levels in the LPM at 24 hpf, when the foregut endoderm, including the prospective hepatoblasts, reside at the embryonic midline, revealed a 23% higher level of EphB3b throughout the right LPM compared with the left LPM (n = 9, p = 0.0095; Figures 7A–7C). This difference is more pronounced at the plasma membranes where EphB3b levels are 70% higher at the right LPM-endoderm interphase than on the left (n = 3, p = 0.0197; Figures 7A and 7C). Shortly after, at 26 hpf, when leftward hepatoblast movement is initiated, the average expression of EphB3b is still 21% higher throughout the right LPM compared with the left. EphB3b membrane localization on the left LPM is very low or absent next to leftward migrating hepatoblasts, in line with a permissive environment for outgrowth. The spatially confined EphB3b expression is maintained and more distinct at 32 hpf (Figures 4B–4B‴). This indicates that differences of EphB3b levels and distribution between the left and the right LPM epithelia are established prior to morphological signs of asymmetric liver outgrowth.

If the LPM provides repulsive EphB3b cues to control hepatoblast positioning, then ectopic expression of EphB3b should re-direct hepatoblasts. To test this, we generated a truncated form of EphB3b lacking the intracellular domain, EphB3b^ΔICD^, that stimulates reverse signaling upon interaction with EphrinB ligands without eliciting forward signaling (Zimmer et al., 2003). By conditional expression of EphB3b^ΔICD^ at 26 hpf, we generated 38 clones in the liver area (38/>200 embryos) with 16 on the left side in domains with no or low endogenous EphB3b. In contrast to controls, in 81% of these embryos (13/16), liver morphology was disrupted with hepatoblasts turning around and moving to the right side, in many cases ventrally to the gut and right LPM (Figures 7D–7F′). This high correlation of altered hepatoblast location and clone position cannot be explained solely by LPM movement defects, because 19% of embryos analyzed at 32hpf (3/16) and 75% analyzed at 28–30 hpf (3/4) with ectopic liver budding still show asymmetric migration of the LPM (data not shown). The gut is mostly in its normal position to the left of the midline, indicating that liver and gut progenitors can move independently from each other and that EphB3b provides directional cues. This is further supported by experiments in which transient injection of *ephrinb1* gRNAs/Cas9 causes mosaic depletion of endogenous EphrinB1 (Figure S4). If EphB3b would not repel EphrinB1-positive hepatoblasts, they should distribute randomly among the EphrinB1-negative cells, as observed in controls in which hepatoblasts mosaically express lyn-Tomato (Figures S4A, S4A′, and S4C′). However, in 9/10 embryos, the EphrinB1-positive hepatoblasts accumulate in the budding area separated from the LPM by 1–6 hepatoblasts expressing no or low level of EphrinB1 (Figure S4). This corroborates that EphrinB1-positive hepatoblasts move away from EphB3b and that they sense EphB3b not only at the direct tissue interface but also over longer distances. Cell interactions away from the tissue interface are further supported by the observation that several cell layers of hepatoblasts next to an EphB3b^ΔICD^ clone show no EphrinB1 at the membrane (Figures 7E′–7F′), in line with direct Eph/Ephrin interaction-triggered removal of the complex by endocytosis (Pitulescu and Adams, 2010).

## Discussion

Our studies provide functional evidence that liver bud formation and its asymmetric positioning require the coordinated movement of two tissues: the hepatic endoderm and the adjacent LPM (Figure S5). Filopodia-like protrusions extending over several cell diameters create LPM-hepatoblast contacts away from the immediate tissue interface, indicating long-distance interactions. Moreover, we identify EphrinB1 in the liver and EphB3b in the LPM as the bidirectional molecular link orchestrating the interconnected movement of both tissues by regulating hepatoblast motility and orientation, and the differentiation of the highly polarized LPM epithelia critical for LPM migration. We propose that the LPM directs the liver into its position by a repulsion-based mechanism, uncovering an additional mechanism for generating left-right tissue asymmetries.

Previous studies suggested that gut looping and digestive organ asymmetry is the result of actively rearranging and moving mesodermal tissues pushing the passive endoderm into position, including the zebrafish liver (Davis et al., 2008, Hecksher-Sorensen, 2004, Horne-Badovinac, 2003). Our findings indicate that zebrafish hepatoblasts are already in an asymmetric position at a stage before overt signs of gut looping (Figures 7A and 7A′) and leftward liver bud positioning is a consequence of active and coordinated rearrangement of both the LPM and endodermal hepatoblasts. By tracking individual cells during budding, we show that hepatoblasts migrate, forming dynamic filopodia and lamellipodia. Compared with adjacent gut progenitors, hepatoblasts are displaced more directionally and generally over a greater distance, supporting their active role in liver positioning. In addition, the presence of ectopic hepatoblasts at posterior positions in EphrinB1- and EphB3b-depleted embryos is consistent with impaired active hepatoblast movement or the loss of spatial information, respectively. In line with this idea EphB3b expression in the LPM and gut delineates the right as well as the anterior and posterior limits of the liver bud. Ectopic expression of EphB3b^ΔICD^ on the left side directs hepatoblasts toward the opposite side of the embryo, while gut tissue remains unaffected, supporting asymmetric repulsive EphB3b-EphrinB1 interaction controlling oriented hepatoblast movement. We therefore propose that EphB3b from LPM cells interacts with EphrinB1 at hepatoblast membranes having a local effect on the cytoskeleton resulting in protrusion collapse, lamellipodia orientation, hepatoblast polarization, and directional migration. These hepatoblast-LPM interactions are reminiscent of morphogenetic movements at the ectoderm-mesoderm border during *Xenopus* gastrulation, where Eph/Ephrin control protrusive activity and cycles of cell attachment and detachment indicating repulsive activity between the two tissues (Rohani et al., 2011). In addition to providing the spatial cues for migrating hepatoblasts via activation of reverse signaling, EphB3b forward signaling mediates the differentiation of a highly polarized LPM epithelium underlying the asymmetric migration of this tissue. Asymmetric LPM migration in turn ensures that the interaction interface and the signaling between the LPM and hepatoblasts is maintained during progressive leftward outgrowth. Unlike the existing model for asymmetric liver positioning, our results indicate that (1) hepatoblasts actively migrate into the liver bud, (2) the LPM directs the movement of hepatoblasts during budding by triggering cell repulsion, and (3) the endoderm signals back to the LPM epithelium controlling its epithelial organization and asymmetric migration. Furthermore, our study supports the notion that neuronal guidance factors have functions outside the developing nervous system (Adams and Eichmann, 2010), including endoderm morphogenesis (Domyan et al., 2013, Klein et al., 2011).

The unique characteristic of EphrinB/EphB bidirectional signaling in eliciting specific cellular responses allows the execution of specialized behaviors of each cell population. In the LPM, EphB3b controls the differentiation of the initially squamous epithelia into a highly polarized morphology, which is prerequisite for the distinct migration of the left and right LPM (Horne-Badovinac, 2003). This is reminiscent of EphB receptors promoting mesenchymal to epithelial transition in several cancer cells (Chiu et al., 2009, Cortina et al., 2007) and in the developing zebrafish where EphB4a regulates epithelialization of somites (Barrios et al., 2003). In hepatoblasts, EphrinB1 controls cell-shape changes and the formation of cellular protrusions. EphrinB1 and EphB3b knockdowns exhibit similar mis-positioning of hepatoblasts, however they unexpectedly result in opposite defects in protrusion formation indicative of EphB3b-dependent and -independent functions of EphrinB1 during budding. EphB-independent functions of EphrinBs were reported in cultured cells where expression of mutant EphrinB variants unable to interact with EphB led to a dramatic increase of filopodia or cell-shape changes (Bochenek et al., 2010, Tomita et al., 2006). We show that EphrinB1 controls protrusion formation via its N-terminal PDZ-binding domain. Interaction between EphrinBs and several PDZ-domain-containing proteins has been reported to occur constitutively and to be antagonized by receptor-mediated activation of EphrinBs (Brückner et al., 1999). This is consistent with our observation that hepatoblasts have more protrusions upon EphB3b knockdown, suggesting that EphB3b-EphrinB1 binding may destabilize protrusions via tyrosine phosphorylation. The different effect on hepatoblast protrusions could also be explained by compensation through other Eph receptors interacting with EphrinB1, however the similarity of the phenotypes after knockdown of EphB3b or expression of the extracellular domain of EphrinB1 suggests that EphB3b is the main receptor of EphrinB1 in this context.

Cell-cell communication is essential for guiding moving cells and coordinating these movements with those of other tissues. In contrast to tissue interaction via secreted signals, communication by cell protrusions allows the signal to be delivered to the receiving cell with a high degree of control and at a distance. Protrusion formation and their functions are best understood in isolated cells in culture (Faix et al., 2009, Ridley, 2011), while this is less clear in the multicellular context of the developing embryo. Several recent studies identified cellular extensions in developing tissues with roles in long-distance signaling. Live imaging in *Drosophila* wing disc first uncovered cytonemes, very long actin-rich protrusions involved in gradient formation and tissue patterning that transport Hedgehog or the Bmp ligand Dpp to receiving cells at a distance (Bischoff et al., 2013, Roy et al., 2014). Short filopodia, have been implicated in establishing the bristle pattern of the *Drosophila* notum by Notch signaling (Cohen et al., 2010) and patterning the zebrafish neural plate by transport of Wnt8 (Stanganello et al., 2015). Our data support the involvement of protrusions in choreographing interlinked tissue movements in liver morphogenesis. Using sparse labeling, we uncovered that both hepatoblasts and LPM cells form protrusions, which ensure that morphogenetic movements are precisely controlled not only at the immediate interface between the two tissues but throughout the entire tissue. Moreover, in all of the above examples, distinctions are made between filopodia sending or receiving signals, whereas, in the liver bud, protrusions likely mediate EphrinB1/EphB3b bidirectional signaling. Two lines of evidence support that cellular protrusions are involved in communication between hepatoblasts and LPM at the direct tissue interface and over a distance: (1) several rows of hepatoblasts adjacent to an EphB3b^ΔICD^ clone display no EphrinB1 at the membrane, which is likely due to endocytosis of the Ephrin/Eph complex following direct cell-cell contacts (Pitulescu and Adams, 2010) and (2) in transient *ephrinb1* CRISPR/Cas9 experiments, EphrinB1-positive hepatoblasts accumulate away from the EphrinB1-EphB3b tissue interface in the budding area separated from the LPM by 1–6 hepatoblasts expressing no or low level of EphrinB1 (Figure S4), strongly supporting long-distance intercellular communication. Although basal filopodia were reported to assist signaling within an epithelium in *Drosophila* (Callejo et al., 2011, Cohen et al., 2010), we propose a rare scenario with protrusions facilitating contact-dependent communication between different tissue types, the epithelial LPM and mesenchyme-like hepatoblasts in the context of liver budding (Figure S5). Coordinating the collective movement of tissues with different architecture by cell protrusions is an effective mechanism for communication over a distance, which is likely relevant in many other tissue contexts including invasive transformed cells.

## Experimental Procedures

All experiments were performed in agreement with the NIMR and KU ethical review committees.

### Generation of Transgenic Lines

Standard cloning and transgenesis techniques were used to generate TgBAC(prox1a:GalTA4-4xUAS-E1b:uncTagRFP)^nim5^, Tg(UAS-E1b:lyn-Citrine)^nim23^, Tg(UAS-E1b:ephrinB1)^nim24^ Tg(UAS-E1b:ephrinB1^EC^)^nim25^, and Tg(hsp70l:gal4)^fci1^.

### Generation of Genetic Mutants

*ephrinb1*^*nim26*^ mutants were generated by CRISPR (clustered regularly interspaced short palindromic repeats)/Cas9 injections into one-cell-stage zebrafish embryos. Oligonucleotides targeting the genomic sequence 5′-GGA CAT TAT CTG CCC CAA AG-3′ in the second exon of the zebrafish *ephrinb1* locus were cloned into the pDR274 plasmid for gRNA production (Hwang et al., 2013). Mutations were identified by amplicon restriction using the primers efnb1F 5′-GTT TGT GTC TGG GAA GGG CTT AG-3′ and efnb1R 5′-TAT GGT GCT GCA GGA CTC GGC CTG-3′, followed by XcmI restriction and verification by sequencing. A stable line *ephrinb1*^*nim26*^ was raised carrying a 4 bp deletion and a 3 bp insertion causing a frameshift and the occurrence of a premature stop codon at position 54. Immunostaining for EphrinB1 revealed a complete absence of protein in homozygous embryos, indicating a complete loss of function.

### Immunostaining and mRNA Stainings

Rabbit α-Prox1 (Chemicon), mouse α-Prox1 (Abcam), and mouse α-ZO1 (Invitrogen) were used for immunostainings. Polyclonal antibodies against EphrinB1 and EphB3b were produced in rabbit and guinea pig, respectively.

Whole-mount in situ hybridization was performed with antisense mRNA probes for *ceruloplasmin, ephrinb1*, and *hhex*. An *ephb3b* riboprobe was generated from a 690 bp fragment, located 3′ of the Ephrin-binding domain.

### Morpholino Knockdown

Antisense morpholino oligonucleotides (Gene Tools) blocking translation or splicing of *ephrinb1* (*MOatg-ephrinb1*, *MOdon-ephrinb1*) and *ephb3b* (*MOdon-ephb3b*, *MOacc-ephb3b*) were injected into one-cell-stage embryos. Morpholinos with 5 bp mismatches were injected as controls (*MOdon-mism-ephrinb1*, *MOacc-mism-ephb3b*), producing no consistent phenotypes.

### Injection of DNA Constructs

*ephrinb1*, *ephrinb1*^*6F*^, and *ephrinb1*^*ΔV*^ were placed behind the *hsp70l* promoter and between *minitol2* sequences. They were co-injected with *transposase* mRNA (30 pg mRNA and 20 pg DNA/embryo) in one-cell-stage embryos and subjected to heat shocks at 39°C: 45 min at 22 hpf and 30 min at 28 hpf. *lyn-tdTomato*, *lyn-citrine*, and *utrophin-gfp* were placed behind the *ubiquitin* (*ubi*) promoter and between *minitol2* sequences and injected as above.

### Quantification of Hepatoblast Characteristics

Membrane-tethered fluorescent proteins were expressed in single cells or small clones by injection of DNA constructs, in which lyn-tdTomato and lyn-Citrine are under the control of the *ubiquitin* promoter, or by applying a binary transgenic approach for which the *TgBAC(prox1a:KalT4-4xUAS:uncTagRFP)*^*nim5*^ transgenic line (hereafter referred to as *Tg(prox1a:KalT4)*^*nim5*^ is crossed with *Tg(UAS:lynCitrine)*^*nim23*^. Notably, the stable *Tg(UAS:lynCitrine)*^*nim23*^ line shows mosaic expression, likely due to partial silencing. Hepatoblast shape was determined by measuring their length and width in coronal or transverse sections of whole-liver confocal stacks to calculate the L/W ratio.

Hepatoblast protrusions were manually tracked in three dimensions through consecutive sections of confocal stacks from whole-mount livers (coronal views). Protrusions with a diameter ≤1.5 μm were classified as filopodia-like (simple and branched), while flat protrusions with larger diameter were classified as lamellipodia-like. Protrusion quantity was determined as the absolute number per square micrometer of cell surface.

The orientation angle of hepatoblasts or their protrusions was determined with respect to the anteroposterior axis of the embryo. All measurements were performed with Volocity software (Improvision).

## Author Contributions

J.C. and E.A.O. designed the study, analyzed data, and wrote the manuscript. J.C. performed experiments. A.D. and S.C. developed the live-imaging strategy. A.D. generated and analyzed time-lapse data; G.K. developed the routine for cell-displacement analysis. J.B. and G.J.W. generated reagents for antibody production. J.C.F. generated transgenic lines. J.C., A.D., S.C., and E.A.O. edited the manuscript.

## Supplemental Information

Document S1. Supplemental Experimental Procedures, Figures S1–S5, and Table S1

Movie S1. Time Lapse Showing Two Phases of Directional Hepatoblast Migration during Liver Bud Formation, Related to Figure 1

Movie S2. Sparse Labeling of Hepatoblasts Reveals Two Phases of Directional Migration during Liver Bud Formation, Related to Figure 1

Movie S3. Migrating Hepatoblasts Form Filopodia- and Lamellipodia-Like Protrusions during Budding and the Onset of Outgrowth, Related to Figure 2

Movie S4. Foregut Organ Formation Proceeds Normally during Live Imaging, Related to Figure 1

Document S2. Article plus Supplemental Information
