# Supplementary material for: EphrinB1/EphB3b Coordinate Bidirectional Epithelial-Mesenchymal Interactions Controlling Liver Morphogenesis and Laterality
Source: Dev Cell. 2016 Nov 7;39(3):316–28. doi: 10.1016/j.devcel.2016.10.009 (PMC5107609; doi:10.1016/j.devcel.2016.10.009)
Supplement: Document S1. Supplemental Experimental Procedures, Figures S1–S5, and Table S1 [file mmc1.pdf]

Developmental Cell, Volume *39*

Supplemental Information

EphrinB1/EphB3b Coordinate Bidirectional Epithelial-Mesenchymal Interactions Controlling Liver Morphogenesis and Laterality

Jordi Cayuso, Aliaksandr Dzementsei, Johanna C. Fischer, Gopal Karemore, Sara Caviglia, Josefin Bartholdson, Gavin J. Wright, and Elke A. Ober

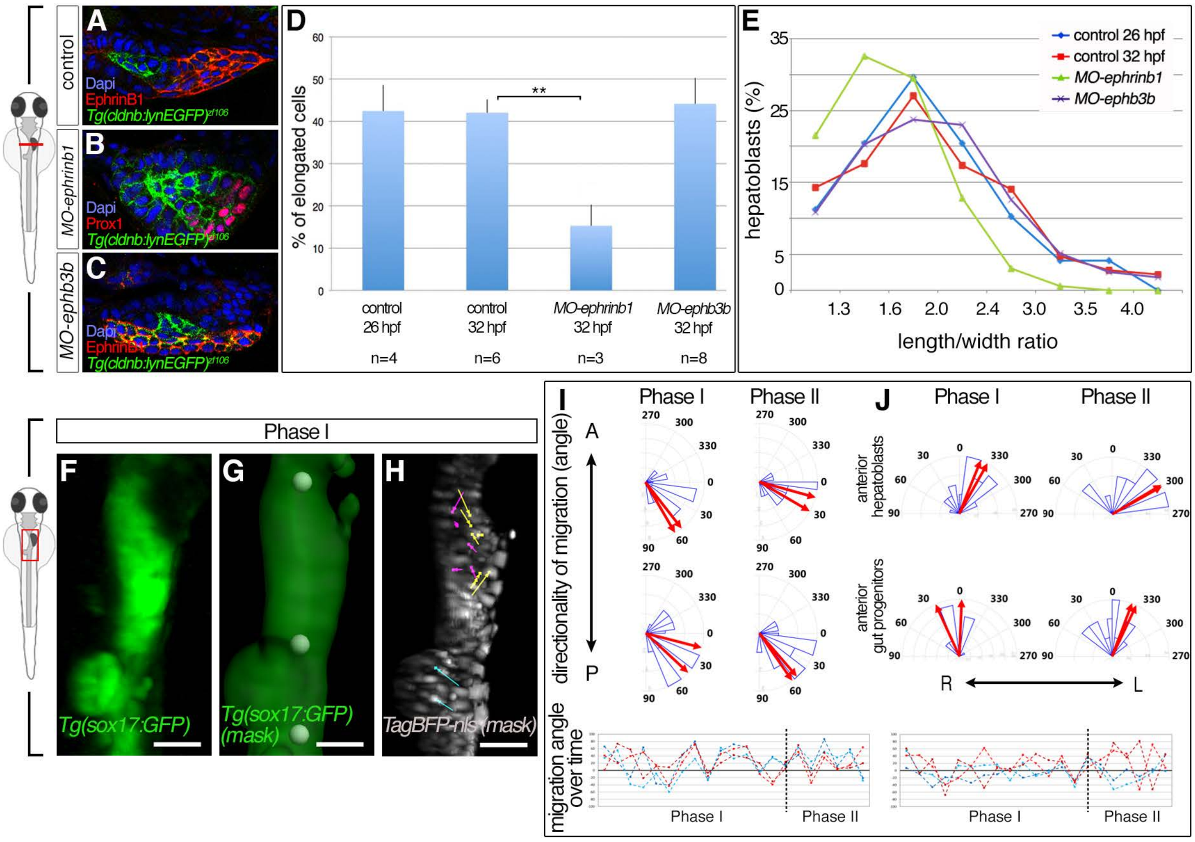

Cayuso et al. Figure S1

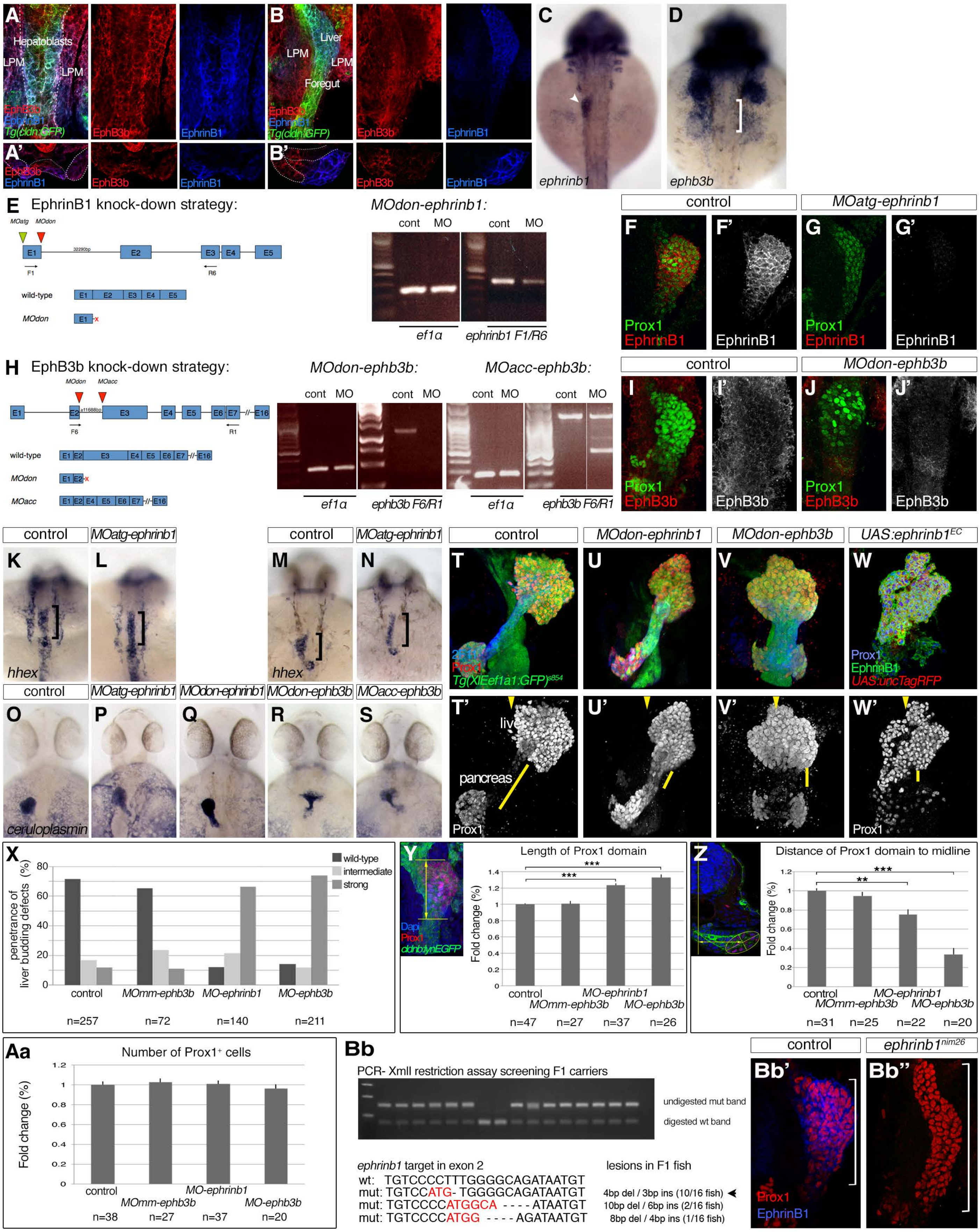

Cayuso et al. Figure S2

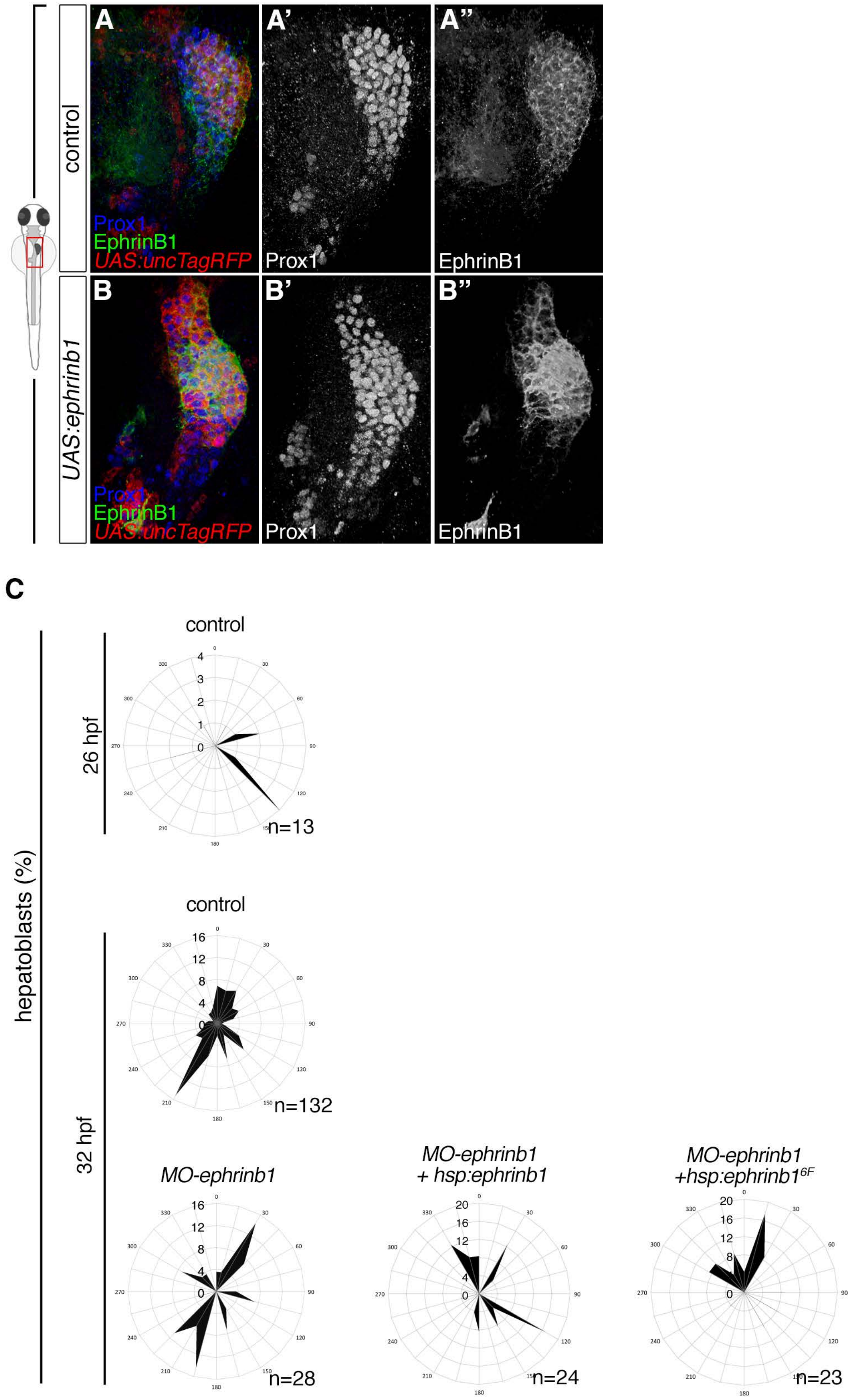

Cayuso et al. Figure S3

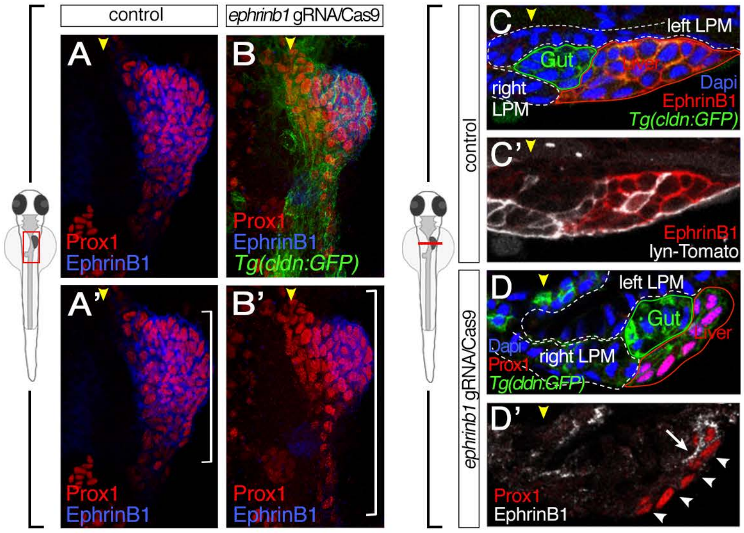

Cayuso et al. Figure S4

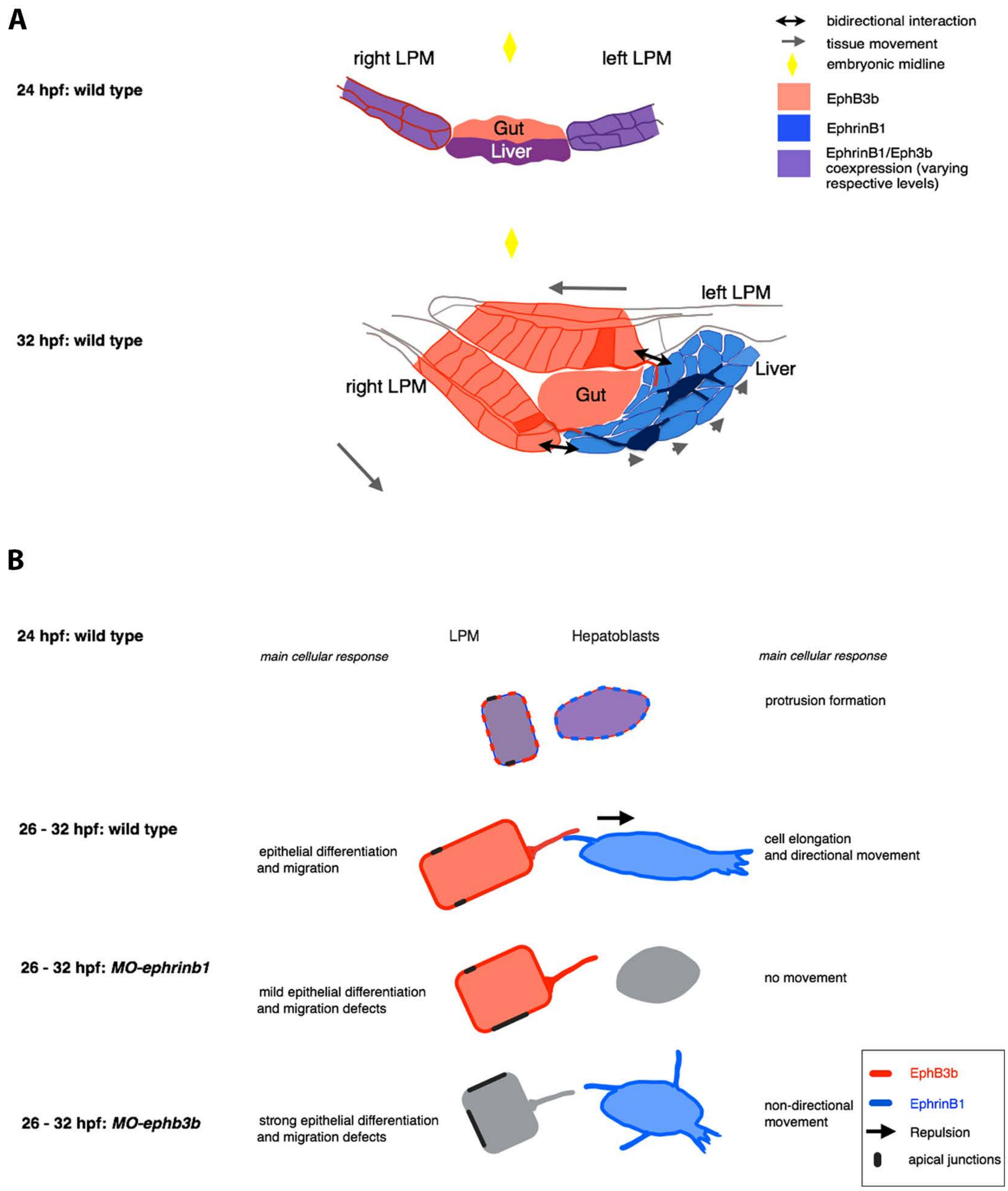

Cayuso et al. Figure S5

Supplemental legends

Supplemental Table S1: Table displaying changes in hepatoblast protrusion formation by comparing different control stages and/or diverse experimental conditions at 32 hpf. Related to Figure 6. p-values were determined by student's t-test (*, p<0.05; **, p <0.01; ***, p <0.001).

Supplemental Figure S1: Hepatoblasts undergo dynamic cell shape changes during liver budding. Related to Figure 1.
(A-C) Cell morphology was determined with EphrinB1- or *Tg(-0.8cldnb:lynEGFP)*^{zf106}-expression outlining the hepatoblasts in control (A), *MO-ephrinB1* (B) and *MO-ephB3b* embryos (C); transverse sections with liver oriented to the right. Morphometric measurements were carried out on serial sections through the bud. **(D,E)** Quantification of hepatoblast characteristics during liver budding in control, *MO-ephrinB1* and *MO-ephB3b* embryos: (D) population of elongated cells (length/width$\geq$2) per bud and (E) hepatoblast distribution according to length/width ratio; only *MO-ephrinB1* exhibit a significantly decreased length/width ratio). Error bars represent standard error of the mean; ** = p<0.01. **(F-H)** Processing steps of 4D dataset of a *Tg(sox17:GFP)*^{z99} embryo injected with *tagBFP-nls* mRNA capturing liver budding (see Figure 1J-N, movie S1). *sox17:GFP* expression (F) marks the endoderm and was used to generate a foregut mask (G) which was subsequently applied to the TagBFP-nls channel (H). Three manually selected anatomical landmarks in 3D were used for drift correction (G, round spheres). Scale bar - 40µm. **(I,J)** Rose plots represent distribution of angular displacement (blue sectors) for hepatoblasts and gut progenitors from the same anterior position and angle of mean displacement per cell for the entire period (red arrow). Displacements were recorded every 28 min with respect to the embryonic midline and left-right distribution. Line plots representing directionality of displacement over time reveal differences in individual angular cell displacement for various hepatoblasts (red hues) and gut progenitors (blue hues). Data are derived from movie S1 (see also Figure 1 J-N).

Supplemental Figure S2: EphrinB1 and EphB3b expression and validation of knock down and knock out strategies. Related to Figure 4.
(A-B') Single channel views of immunostainings for EphrinB1 and EphB3b at 22 and 32 hpf shown in Figure 4A-B'''. **(C,D)** *ephrinb1and ephb3b* are expressed in the foregut area. At 30 hpf, *ephrinb1* is expressed in the liver (arrowhead) and *ephb3b* in hepatic area (bracket). **(E)** Schematic representation of *ephrinB1* knock-down strategy, with relevant MOs indicated. RT-PCR analysis of *MOdon-ephrinB1* embryos at 28 hpf shows a strong knock-down. In addition, a stop codon in the intronic sequence terminates the resulting protein 3 bps after the exon1-intron boundary. Both *MO-ephrinB1* produce similar phenotypes (see O-Q), *MOatg-ephrinB1* were used for all experiments. **(F-G')** EphrinB1 expression is abolished or barely detectable in *MOatg-ephrinB1* embryos at 32 hpf. **(H)** Schematic representation of *ephB3b* knock-down strategy, with relevant MOs indicated. *ephB3b* maps to linkage group 22 and is partly encoded by EST CT 623350. By 5'RACE, we identified a 2970 bp coding transcript encoded by 16 exons containing all motifs conserved between EphB-receptors, including a N-terminal signal peptide and Ephrin-binding domain. In addition, we found an alternative transcript missing the first two exons, replaced by a N-terminal extension of exon 3 and lacking a signal peptide and a clear transcriptional start site. Its functional significance is unclear and it was not targeted by our knock-down strategy. RT-PCR analysis of *MOdon-ephB3b* embryos at 28 hpf shows a complete knock-down, indicated by a complete loss of the Ephrin-binding domain-containing exon3. In addition, a stop codon in the intronic sequence would terminate the resulting protein 108 bps after the exon 2-intron boundary. RT-PCR analysis of *MOacc-ephB3b* embryos at 28 hpf reveals a partial knock-down. This is consistent with the loss of the Ephrin-binding domain-containing exon 3 in a fraction of the transcripts, as indicated by the appearance of a 500bp smaller ectopic band in the *MOacc-ephB3b* lane compared to controls; control and representative *MOacc-ephB3b* sample are from different parts of the same gel, as indicated by the separating line. Although, both *MO-ephB3b* produce similar phenotypes (see O,R,S), the *MOdon-ephB3b* knock-down is consistently more complete and was used for all experiments. **(I-J')** EphB3b expression is abolished or barely detectable in *MOdon-ephB3b* embryos at 32 hpf. **(K-N)** *hhex* mRNA expression at 25 hpf reveals that the liver anlage (bracket) forms in *MO-ephrinB1* embryos (L) similar to controls (G), while impaired hepatoblast (bracket) movement into the liver bud in *MO-ephrinB1* embryos is apparent at 28 hpf (M,N). **(O-S)** Two independent MOs targeting *ephrinB1* or *ephB3b* produce equivalent phenotypes, with hepatoblasts positioned more posterior and medial (P,Q and R,S, respectively) compared to controls at 48 hpf (O). Asymmetric hepatoblast positioning is generally more severely disrupted in *MO-ephB3b* than *MO-ephrinB1* embryos. **(T-W')** Confocal projections reveal liver morphogenesis defects in *MO-ephrinB1* (U,U'), MO-*ephB3b* (V,V'),

as well as embryos expressing EphrinB1^{EC} in the context of the entire foregut domain (W,W'). *Tg(XlEef1a1:GFP)^{s854}* marks the foregut endoderm, 2F11 the hepatopancreatic ducts and Prox1 the developing liver and pancreas at 54 hpf. Upon loss of EphrinB1 or EphB3b functions hepatoblasts are ectopically positioned in the hepatopancreatic duct domain compared to controls, as indicated by shorter yellow bars, and across the midline (arrowheads; T'-V'). Similar defects are observed upon conditional expression of EphrinB1^{EC} (W,W'). (C,D, K-S) dorsal views, (A-B, F-G', I-J',T-W') confocal projections of ventral views; all anterior to the top. (A',B') confocal images of transverse sections, left is oriented to the right.
(X-Aa) Quantification and morphometric analyses of liver bud development in *MO-ephrinB1* and *MO-ephB3b* embryos at 32 hpf. **(X)** Graph showing the proportion of embryos exhibiting hepatoblast positioning defects in un-injected and *MOmm-ephB3b* controls, and following EphrinB1- or EphB3b knockdown. In whole-mount preparations the length (Y) and width of the Prox1-domain and its proximity to the embryonic midline (Z) were used to classify the liver budding defects into three categories. **(Y)** Morphometric measurements showed a significant increase in Prox1-domain length (yellow double arrow) in *MO-ephrinB1* and *MO-ephB3b* embryos compared to controls. **(Z)** Morphometric measurements revealed a significant reduction in asymmetric displacement of the Prox1-domain from the midline in *MO-ephrinB1* and *MO-ephB3b* embryos compared to controls. Specifically, the distance of the centre of the Prox1-domain to the embryonic midline was determined in transverse sections (yellow double arrow). **(Aa)** EphrinB1 or EphB3b knockdown does not significantly change the number of Prox1-expressing hepatoblasts; p-values, for all conditions >0.57. (c,e,f) Standard errors are shown; asterisks indicate the p-value for each condition (**, $p<0.01$; ***, $p<0.001$). **(Bb-Bb")** Stable genetic *ephrinb1* mutant was generated using Crispr/Cas9 genome editing. F1 fish were screened for germline transmission of lesions by XmlI digest of amplified target DNA; undigested PCR bands indicate transmission of genetic lesion. Sequencing revealed that F1 fish carry different indels. Intercrosses of carriers with a 4bp deletion and 3bp insertion show a complete loss of α-EphrinB1 staining in homozygous carriers (Bb") compared to sibling controls (Bb'). A stable line, *ephrinb1^{nim26}*, was generated from these carriers.

Supplemental Figure S3: Altering EphrinB1 and EphB3b levels leads to defects in lamellipodia orientation and hepatoblast positioning. Related to Figure 6.
(A-B") Conditional expression of *UAS:ephrinB1* causes a posterior accumulation of hepatoblasts and morphological defects of the liver domain (B-B"). **(C)** Hepatoblast lamellipodia are oriented with respect to the anteroposterior axis. Expression of EphrinB1 and EphrinB1^{6F} randomises lamellipodia orientation in comparison to control and *MO-ephrinB1* embryos, and similar to compromised EphB3b function (see Figure 6H).

Supplemental Figure S4: Mosaic inactivation of EphrinB1 by Crispr/Cas9 editing, leads to defects in hepatoblast positioning and indicate long-distance contacts. Related to Figure 7.
(A-B') at 32 hpf, EphrinB1^{+}-hepatoblasts transiently expressing lyn-Tomato distribute randomly throughout the bud (A,A'), while EphrinB1^{+} hepatoblasts (blue) aggregate mostly anterior and away from the right LPM and hepatoblasts lacking EphrinB1 form an elongated Prox1 domain (B,B') closer to the midline (yellow arrowhead). White brackets indicate the length of the Prox1-domain. Confocal projections of ventral views; anterior to the top. **(C-D')** EphrinB1^{+} hepatoblasts (white) aggregate mostly away from the right LPM, often with a gap of 1-6 hepatoblasts lacking EphrinB1 (white arrowheads) indicating long-distance cell-cell contacts sensing repulsive EphB3b from the LPM. Consistently, hepatoblast protrusions (arrow) are also EphrinB1^{+} (D'). Transverse sections with dorsal to the top, and left to the right.

Supplemental Figure S5: Working model of how bidirectional EphrinB1/EphB3b activity coordinates hepatoblasts and LPM movements in liver budding. Related to Figure 7.
(A) EphrinB1 and EphB3b are dynamically expressed during liver budding. At 24 hpf, the start of budding both proteins are co-expressed in the LPM epithelia and newly specified hepatoblasts. Notably, EphB3b is higher and at the membrane in the right than left LPM, and the foregut solely expressed EphB3b. Co-expression is associated with cis-inhibition of signalling. By 32 hpf, EphrinB1 and EphB3b domains are complementary with EphrinB1 solely in hepatoblasts, and EphB3b in the LPM epithelia and gut. Single LPM cells (dark blue) and hepatoblasts (bright red) are highlighted to visualise cell protrusions and complex morphologies representative for all cells in the respective tissue. They reveal long-distance contacts between hepatoblasts and LPM cells allowing bidirectional EphrinB1 and EphB3b signalling (double headed arrows) essential for coordinated hepatoblast and

LPM movements (grey arrows). **(B)** Schematic representation of main cellular behaviours elicited by EphrinB1 and EphB3b in hepatoblasts and LPM epithelial cells, respectively.

Movie S1: Time lapse showing two phases of directional hepatoblast migration during liver bud formation. Related to Figure 1.
Tracking of liver (yellow), gut (magenta) and pancreas (white) progenitors in the *Tg(sox17:GFP)*-positive (green) foregut region; dorsal view. Cell nuclei were labelled by injection of *TagBFP-nls* mRNA (grey). Images were taken every 7 min and the monitored period is equivalent to 24 -34 hpf of development. Stills are shown in Figure 1. Scale bar - 40μm.

Movie S2: Sparse labelling of hepatoblasts reveals two phases of directional migration during liver bud formation. Related to Figure 1.
Tracking of liver (yellow), gut (magenta) and pancreas (white) progenitors in *Tg(sox17:GFP)*-positive (green) foregut region; dorsal view. Cell nuclei were labelled by DNA injection of *UAS:TagBFP-nls* (cyan) in embryos with transgenic expression of *prox1a:kalTA4-4xUAS:tagRFP* (red) coming up predominantly in hepatocytes. Images were taken every 28 min and the monitored period is equivalent to 24 -34 hpf of development; scale bar - 40μm.

Movie S3: Migrating hepatoblasts form filopodia- and lamellipodia-like protrusions during budding and the onset of outgrowth. Related to Figure 2.
Snapshots are included in Figure 2E. Transgenic *UAS:lyn-Citrine* (green) labels cell membranes. Images were taken every 23,5 min and the monitored period is equivalent to 24 -34 hpf of development; scale bar - 40μm.

Movie S4: Foregut organ formation proceeds normally during *live*-imaging. Related to Figure 1.
Dorsal view of the embryo with transgenic expression of *sox17:GFP* (green) marking the digestive system, *prox1a:kalTA4-4xUAS:tagRFP* (red) highlighting the liver and DNA injection of *UAS:TagBFP-nls* (cyan) marking nuclei. Images were taken every 21,5 min and the monitored period is equivalent to 24 -56 hpf of development; scale bar - 100μm.

	lamellipodia/μm^2	filopodia (branched)/μm^2	filopodia/μm^2
**control 26hpf** **vs.** **control 32hpf**	** 0.0041	*** <0.0001	0.5277
**control 32 hpf** **vs.** ***MO-ephb3b***	0.5488	*** <0.0001	0.2059
**control 32 hpf** **vs.** ***MO-ephrinb1***	*** <0.0001	* 0.0105	0.6451
**control 32 hpf** **vs.** ***UAS:ephrinb1***	0.4496	*** <0.0001	** 0.009
MO-ephrinb1 **vs.** ***MO-ephrinb1+hsp:ephrinb1***	*** <0.0001	*** <0.0001	*** <0.0001
MO-ephrinb1 **vs.** ***MO-ephrinb1+hsp:ephrinb1***$^{\Delta V}$	0.3918	*** <0.0001	0.5283
MO-ephrinb1 **vs.** ***MO-ephrinb1+hsp:ephrinb1***^{6F}	* 0.0177	*** <0.0001	*** 0.0003
MO-ephrinb1+hsp:ephrinb1 ***vs.*** ***MO-ephrinb1+hsp:ephrinb1***$^{\Delta V}$	*** <0.0001	0.8794	** 0.0092
MO-ephrinb1+hsp:ephrinb1 ***vs.*** ***MO-ephrinb1+hsp:ephrinb1***^{6F}	0.105	0.3358	0.827

Cayuso et al. Table S1

Experimental Procedures

Fish stocks

Adult zebrafish and embryos were raised according to standard laboratory conditions (Westerfield, 2000). The following published strains were used: *Tg(XlEef1a1:GFP)^{s854}* (Field et al., 2003), *Tg(-0.8cldnb:lynEGFP)^{zf106}* (Haas and Gilmour, 2006), *Tg(-0.5sox17:GFP)^{zf99}* (Mizoguchi et al., 2008), *casanova/sox32^{ta56}* (Alexander et al., 1999) and wild-type. All experiments were performed in agreement with the NIMR and KU Ethical review committees.

Generation of transgenic lines

For the *prox1a BAC:kalTA4-4xUAS:uncTagRFP* transgenic driver line, a GalK cassette was recombined into the BAC clone DKEY-5J3 using PCR primers tagged with 75bp homology arms, and subsequently replaced with the final cassette (Distel et al., 2009; Warming et al., 2005) flanked by 470bp homology arms. Stable transgenic carriers were generated by injecting 25-100 pg BAC DNA into one-cell stage embryos, and screening adult fish for red fluorescent TagRFP expression in the liver. *TgBAC(prox1a:kalTA4-4xUAS:uncTagRFP)^{nim5}* are currently maintained in the F5 generation and was first described in (Dunworth et al., 2014).
Responder vectors were generated by cloning *lyn-Citrine,* full-length *ephrinb1* and *ephrinb1^{EC}*, encoding the extracellular domain and encompassing amino acids 1-236, under the control of five UAS sites and the E1b basal promoter (Köster and Fraser, 2001). To facilitate the identification of transgenic carriers, these constructs contain in addition *citrine* under the control of the lens-specific *α-Crystallin* promoter (Kurita et al., 2003). These constructs are flanked by miniTol2 elements (Urasaki et al., 2006). Stable transgenic carriers were generated by injecting 25 pg vector DNA and 40 pg *transposase* mRNA into one-cell stage embryos and raising embryos with Citrine-positive lenses to adulthood. Stable transgenic lines were established for *Tg(UAS:lyn-Citrine)^{nim23}*, *Tg(UAS:ephrinb1)^{nim24}* and *Tg(UAS:ephrinb1^{EC})^{nim25}*, and are currently maintained in the F4 generation. UAS-mediated transgene expression is predominantly mosaic, as identified by lyn-Citrine expression or α-EphrinB1 stainings. In latter case, positive clones were distinguishable due to significantly higher transgenic EphrinB1 or EphrinB1^{EC} levels, which generally require for the detection by confocal microscopy only 50% of the photomultiplier gain used for endogenous EphrinB1 in the liver.

Immunohistochemistry

Labellings were performed as described (Ober et al., 2006). The following antibodies were used: rabbit α-Prox1 (1:1000; Chemicon), mouse α-Prox1 (1:100; Abcam), mouse α-2F11 (Abcam, 1:1000) and mouse α-ZO1 (1:200, Invitrogen). Cy3- and Cy5-conjugated secondary antibodies were obtained from Jackson Immunoresearch. Embryos were mounted in 4% agarose, sectioned at 70-130 μm using a Leica Vibratome and visualised using a Zeiss LSM5 Pascal Exciter confocal microscope. Images were processed with Volocity image analysis software (PerkinElmer) and Photoshop CS (Adobe). Polyclonal antibodies against EphrinB1 and EphB3b were generated in rabbits and guinea pigs, respectively. The entire ectodomain region including the native signal peptide of zebrafish EphrinB1 and EphB3b were subcloned into a mammalian expression vector containing a C-terminal rat Cd4d3+4 tag followed by a hexa-his tag and expressed as a soluble recombinant protein using transient transfections of HEK293E cells, essentially as described (Bushell et al., 2008). Proteins were purified by Ni^{2+}-affinity chromatography (GE Healthcare). Protein concentration was determined by UV-spectroscopy using a computationally calculated extinction coefficient.

In Situ Hybridisation

Whole-mount mRNA in situ hybridisation was performed as described (Ober et al., 2006). Embryos older than 24 hpf were treated with 0.2mM 1-phenyl-2-thiourea in egg water to inhibit Melanin production. The following probes were used: *ceruloplasmin (Korzh et al., 2001),* and *hhex (Ho et al., 1999).* A riboprobe specific for *ephb3b* was generated cloning a 690 bp fragment, located 3' of the Ephrin-binding domain, into PCRII (Invitrogen). The following primers were used to amplify this sequence: Fw 5'- TCCGGGCTTTCTACAAGAAGT-3' and Rv 5'-AGGGACTCTTGCTGGCTACTC-3'.

Morpholino knockdown

Antisense morpholino oligonucleotides blocking translation or splicing of *ephrinb1* and *ephb3b* (Gene Tools, LLC) were injected into one-cell stage embryos. Morpholinos with 5 bp mismatches (*mm*) were injected as controls, producing no consistent phenotypes. 4 ng *MOatg-ephrinb1*, 2.2 ng *MOdon-*

ephrinb1, 1.5 ng *MOdon-ephb3b,* 1 ng *MOacc-ephb3b* and 1 ng *MOacc-mm-ephb3b* were injected per embryo. Sequences of morpholinos used in this study:
MOatg-ephrinb1 5'-TTGCCGAACCACATGCAACAGAGCG-3'
MOdon-ephrinb1 5'-TGCACTTACTTGGGATTTTGCGAAT-3'
MOdon-ephb3b 5'-TGTAAAAAGACAGCTCACCCCGGTC-3'
MOacc-ephb3b 5'-CCCACTGCATTAAAAAAAGAGAGCA-3'
MOacc-mm-ephb3b 5'-CgCACTGgATTAAAcAAAGAcAGgA-3'
The efficiency of the knockdown was tested by antibody staining against the targeted gene or by RT-PCR after RNA extraction with TRIzol reagent (Invitrogen) from 5 embryos per condition (Supplementary Fig. 1). Sequences of indicated PCR-primers: *ephrinb1*-F1: 5'-TTACCTCTGGATTCTGACAGC-3', *ephrinb1*-R6: 5'-GTTTTCCAAGCCTTCCTGTGT-3', *ephb3b*-F6: 5'-AAACACTGATGGACACCAAATG-3', *ephb3b*-R1: 5'-AGGGACTCTTGCTGGCTACTC-3'. Moreover, MO-injection into a p53-depleted background (*MO-p53* 5'-GCGCCATTGCTTTGCAAGAATTG-3') did not alter the observed liver morphogenesis phenotypes. These results together with the lack of apparent pyknotic nuclei, visualised by Dapi, indicate that MO-mediated EphrinB1 or EphB3b knock-down does not produce apparent unspecific defects, including apoptosis. Altogether, these findings confirm the specificity of the knock-down phenotypes.

Injection of DNA-constructs

Constructs for ubiquitous expression:The membrane-targeted *lyn-tdTomato* and *utrophin-gfp* (Burkel et al., 2007) were placed under the control of the *ubiquitin (ubi)* promoter (Mosimann et al.) and between *minitol2* sequences (Urasaki et al., 2006) and sequence verified.
Heat-shock inducible (hsp70l) constructs: Full-length and mutant forms of *ephrinb1* were placed under the control of the *hsp70l* promoter and between *minitol2* sequences and sequence verified. In $ephrinb1^{6F}$, six conserved tyrosines were replaced by phenylalanine (gene synthesis by Entelechon). Injected embryos were subjected to two heat-shocks at 39ºC: 45 min at 22 hpf and 30 min at 28 hpf. *ephb3bΔICD* lacking the intracellular domain was PCR-amplified using: *ephb3bΔICD-fw* 5'-CCGTCGACATGACAATGGATTATTTG-3'; *ephb3bΔICD*-rev 5'-ATCGAGATCTCGTCGATCCCT-3' primers. A *myc* tag was added to the N-terminus of the protein. Injected embryos were subjected to two heat-shocks at 39ºC: 30 min at 22 and 26 or 28 hpf. Embryos were fixed two or four hours later and immunostained. Positive clones were identified by Myc expression and/or increased EphB3b staining.
Kal4-inducible 5xUAS constructs: tagBFP-nls cassette was amplified from pBluescript-*tagBFP-nls* (Kanca et al., 2014); kind gift from Emmanuel Caussinus) using:
tagBFP-fw 5'-ATATGAATTCGCGTAGGGGATCCCAAAATG-3' and M13-rev 5'-TCACACAGGAAACAGCTATGAC-3' primers. Wild type *cdc42* and dominant negative $cdc42^{T17N}$-*EGFP*-fusion cassettes (kind gift from Steffen Scholpp) were extracted by PCR. All cassettes were cloned downstream of 5xUAS and the E1b basal promoter (Köster and Fraser, 2001). The constructs contain also *citrine* under the control of the lens-specific *α-Crystallin* promoter (Kurita et al., 2003), and it is flanked by *miniTol2* elements. Constructs were injected into *TgBAC(prox1a:kalTA4-4xUAS:uncTagRFP)*^{nim5} or *Tg(hsp70l:Gal4)*^{fci1} (kind gift from David Wilkinson).
All constructs (20-30 pg DNA/embryo) were co-injected with *transposase* mRNA (30-80 pg/embryo).

Latrunculin B treatment

Actin polymerisation was inhibited by Latrunculin B (LatB) (Morton, Ayscough, & McLaughlin, 2000). Given the importance of actin polymerisation for numerous cellular processes, we minimised the exposure to LatB and treated the embryos from 26 -32 hpf with low doses of the drug (0.1μg/ml in egg water). Consistent with a recent study incubating embryos >24 hpf with 0.15 μg/ml Lat B for an extended period of time ($\geq$ 17 hours) (Phng, Stanchi, & Gerhardt, 2013), our short exposure to only 0.1μg/ml Lat B caused no apparent changes to the overall embryonic body phenotype. Likewise, hepatoblast numbers in LatB- and DMSO-treated embryos are similar (DMSO: 111.25 cells, SEM 6.51; Lat B: 105.89 cells, SEM 6.53; $p=0.6$), suggesting that LatB treatment did not significantly effect cell proliferation, another cellular processes that requires functional actin dynamics.

Quantification and morphometric analysis of liver budding

Ventral views of confocal stacks from whole-mount antibody preparations were used to measure the length of the Prox1-domain along the anteroposterior axis as indicated in Figure S2U. The extent of leftward liver displacement was assessed by determining the distance of the centre of the Prox1-domain to the embryonic midline in confocal stacks of transverse sections from whole-mount antibody labellings as depicted in Figure S2V. For this, the first section was randomly chosen and subsequent

sections, every 6 μm, were analysed and the average determined for every embryo. The number of hepatoblasts was determined by automatic quantification of Prox1$^+$ nuclei. All measurements were carried out with Volocity software (PerkinElmer).

2D-Quantification of cell shapes and orientation

Cellular characteristics were determined by measuring length and width of hepatoblasts in coronal or transverse sections of whole liver confocal stacks using Volocity software (PerkinElmer), followed by calculation of the length/width ratio; on average 4 sections per liver and minimum of 3 embryos (n=3). The first section was randomly chosen and subsequently sections every 6 microns were analysed. Statistical significance was determined by Student's t-test. Orientation of elongated hepatoblasts represents the angle of the longest axis of the cell with respect to the anteroposterior axis of the embryo.

3D-Quantification of cell protrusions

Hepatoblast protrusions were manually tracked in three dimensions (x, y and z) through consecutive sections of confocal stacks from whole-mount livers (coronal views) using Volocity software (PerkinElmer). Stacks consist of optical sections acquired at least every 2 μm. Flat protrusions with a diameter $\geq$ 1.5 μm were classified as lamellipodia-like, and ones with a smaller diameter as filopodia-like (regular and branched). The protrusion number was determined as absolute number per μm^2 cell surface. Clone perimeter and height were measured and used to calculate the cell surface. Statistical significance was determined by Student's t-test. The orientation-angle of hepatoblast extensions is displayed with respect to the anteroposterior axis of the embryo.

Statistical analysis

To determine the statistical significance of the data sets, unpaired two-tailed Student's *t*-test was used. $p<0.05$ was used as a criterion for statistical significance in all experiments. Error bars represent s.e.m..

Live-Imaging and analysis

24-26 hpf embryos were embedded in a drop of 0,4% low melt agarose containing 200 μg/ml Tricaine and placed between two coverslips. Space between the cover slips was filled with approx. 1,5 ml of Embryo medium containing 0,2 mM PTU and 200 μg/ml Tricaine and sealed with vacuum grease. 4D (xyzt) imaging was performed using 20x objectives on a Leica SP8 or Zeiss LSM780 confocal microscope. Images were collected every 7-28 minutes (mostly 21 minutes), depending on single or multi-position acquisition. During the imaging process, embryo and digestive system development was delayed without altering development, including the foregut organs (see movie S4). Combinations of the following transgenes and fluorescent labels were used for tracking liver and gut progenitors: *Tg(-0,5sox17:GFP)*^{zf99} marks the endoderm, and *TgBAC(prox1a:kalTA4-4xUAS:uncTagRFP)*^{nim5} labels predominantly hepatoblasts with a mosaic onset. 200pg of *tagBFP-nls* mRNA (Figures 1J-L, S1F-H, movie S1) were injected to mark all nuclei or 20pg of UAS:TagBFP-nls DNA co-injected with 20pg of *Tol2 transposase* mRNA to mark hepatoblast nuclei under the control of *prox1a:kalTA4* (movies S2, S4). Hepatoblast migration on a single cell level and including cellular protrusions was imaged in *TgBAC(prox1a:kalTA4-4xUAS:uncTagRFP)*^{nim5}*;Tg(UAS:lyn-Citrine)*^{nim23} embryos (Figure 2E, movie S3). Sparse labeling is due to mosaic onset of *prox1a:kalTA4* and generally mosaic expression of *UAS:lyn-Citrine*, likely due to silencing. Datasets were reconstructed for subsequent analysis using Imaris BITPLANE software (version 8.1.2). The analysis included the following steps:

1) 3D Drift Correction. Samples often drift during image acquisition reducing the ability for accurately tracking and measuring cell movements over time and therefore need to be corrected for accurate tracking. The displacement of two successive data points over time was determined by normalized cross correlation of their maximum intensity projections. Correction of the whole time series was achieved by transforming each time frame with the cumulated displacements. Drift was corrected using 2-4 manually selected 3D landmarks with anatomical correspondence between all time frames using Imaris BITPLANE software.

2) Mask Generation: GFP signal from *Tg(-0,5sox17:GFP)*^{zf99} was used to create a mask of endoderm cells (Figure S1F,G).

3) Semi-automated Cell Tracking. Cells were tracked in drift-corrected datasets using nuclei marked by TagBFP-nls. An autoregressive motion algorithm was used for automatic cell tracking. Tracks were carefully analysed by at least two users and then corrected manually. Two criteria were used to discriminate between liver and gut progenitor cells: i) the position during liver bud outgrowth phase, which often corresponded to the end of the movie, and ii) *TgBAC(prox1a:kalTA4-4xUAS:uncTagRFP)*^{nim5} expression at the liver bud outgrowth phase; subsequent backtracking

confirmed cell identity. Position coordinates of every tracked cell for all time points were exported to an Excel file.

4) Generating displacement vectors for each time point. Steps 3 to 5 were carried out with MatLab R2016a software. Displacement vectors of a tracked cell were generated by subtracting X, Y and Z coordinates of two successive positions. Displacement of different cell types was determined with respect to a meaningful anatomical reference plane: the anteroposterior plane (A-P) corresponds to the embryonic midline defined at 24-26hpf, and the left-right (L-R) one is perpendicular to the A-P plane. The embryonic midline was defined using reconstructed 3D datasets. Three random points on each plane were selected to generate the corresponding plane equation in MatLab R2016a software. Later a normal vector to these planes was obtained by using point-normal form and general form of the equation of a plane in coordinate geometry.

5) Generating angle between displacement vector and reference plane. Vector geometry formulas i.e. cross product was used to determine the angle between the displacement vectors for each time frame (obtained in step 3) and the unit vector parallel to the A-P or L-R plane.

6) Representation of angular distributions. Rose plots were generated summarizing the angular distribution of displacement vectors with respect to A-P and L-R planes for all time frames. The range of angles is represented in the area of each wedge and the concentric circles represent the observed angle frequency within a particular range. The range of angles is represented by the size of each wedge (bin size), which was set to 15 degree. Line plots show orientation of the displacement vectors with respect to A-P and L-R planes for each time frame.

References:

Alexander, J., Rothenberg, M., Henry, G.L., and Stainier, D.Y. (1999). casanova plays an early and essential role in endoderm formation in zebrafish. Dev Biol *215*, 343-357.

Bushell, K.M., Sollner, C., Schuster-Boeckler, B., Bateman, A., and Wright, G.J. (2008). Large-scale screening for novel low-affinity extracellular protein interactions. Genome Res *18*, 622-630.

Distel, M., Wullimann, M.F., and Koster, R.W. (2009). Optimized Gal4 genetics for permanent gene expression mapping in zebrafish. Proc Natl Acad Sci U S A *106*, 13365-13370.

Dunworth, W. P., Cardona-Costa, J., Bozkulak, E. C., Kim, J.-D., Meadows, S., Fischer, J. C., et al. (2014). Bone morphogenetic protein 2 signaling negatively modulates lymphatic development in vertebrate embryos. *Circulation Research*, *114*(1), 56–66. http://doi.org/10.1161/CIRCRESAHA.114.302452

Haas, P., and Gilmour, D. (2006). Chemokine signaling mediates self-organizing tissue migration in the zebrafish lateral line. Dev Cell *10*, 673-680.

Ho, C.Y., Houart, C., Wilson, S.W., and Stainier, D.Y. (1999). A role for the extraembryonic yolk syncytial layer in patterning the zebrafish embryo suggested by properties of the hex gene. Curr Biol *9*, 1131-1134.

Kanca, O., Caussinus, E., Denes, A.S., Percival-Smith, A., and Affolter, M. (2014). Raeppli: a whole-tissue labeling tool for live imaging of Drosophila development. Development *141*, 472-480.

Korzh, S., Emelyanov, A., and Korzh, V. (2001). Developmental analysis of ceruloplasmin gene and liver formation in zebrafish. Mech Dev *103*, 137-139.

Köster, R.W., and Fraser, S.E. (2001). Tracing transgene expression in living zebrafish embryos. Dev Biol *233*, 329-346.

Kurita, R., Sagara, H., Aoki, Y., Link, B.A., Arai, K., and Watanabe, S. (2003). Suppression of lens growth by alphaA-crystallin promoter-driven expression of diphtheria toxin results in disruption of retinal cell organization in zebrafish. Dev Biol *255*, 113-127.

Mizoguchi, T., Verkade, H., Heath, J.K., Kuroiwa, A., and Kikuchi, Y. (2008). Sdf1/Cxcr4 signaling controls the dorsal migration of endodermal cells during zebrafish gastrulation. Development *135*, 2521-2529.

Mosimann, C., Kaufman, C.K., Li, P., Pugach, E.K., Tamplin, O.J., and Zon, L.I. (2011). Ubiquitous transgene expression and Cre-based recombination driven by the ubiquitin promoter in zebrafish. Development *138*, 169-177.

Morton, W. M., Ayscough, K. R., & McLaughlin, P. J. (2000). Latrunculin alters the actin-monomer subunit interface to prevent polymerization. *Nature Cell Biology*, *2*(6), 376–378. http://doi.org/10.1038/35014075

Phng, L.-K., Stanchi, F., & Gerhardt, H. (2013). Filopodia are dispensable for endothelial tip cell guidance. *Development*, *140*(19), 4031–4040.

Urasaki, A., Morvan, G., and Kawakami, K. (2006). Functional dissection of the Tol2 transposable element identified the minimal cis-sequence and a highly repetitive sequence in the subterminal region essential for transposition. Genetics *174*, 639-649.

Warming, S., Costantino, N., Court, D.L., Jenkins, N.A., and Copeland, N.G. (2005). Simple and highly efficient BAC recombineering using galK selection. Nucleic Acids Res *33*, e36.

Westerfield, M. (2000). The Zebrafish Book. A Guide for the Laboratory Use of Zebrafish (Danio rerio), 4th edn edn (Eugene, OR: University of Oregon Press).
