# Supplementary figures and images for: EphrinB1/EphB3b Coordinate Bidirectional Epithelial-Mesenchymal Interactions Controlling Liver Morphogenesis and Laterality
Source: Dev Cell. 2016 Nov 7;39(3):316–28. doi: 10.1016/j.devcel.2016.10.009 (PMC5107609; doi:10.1016/j.devcel.2016.10.009)

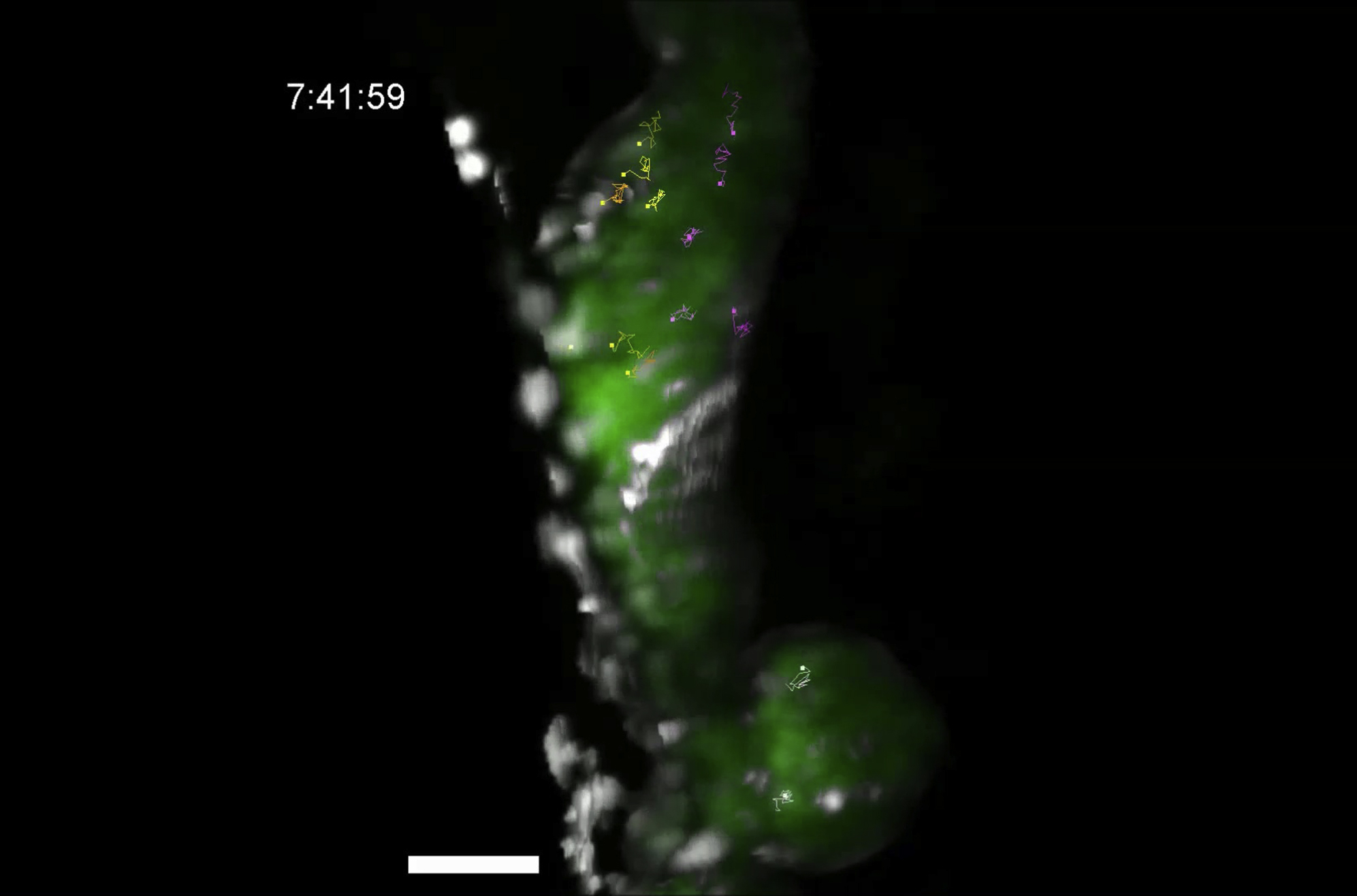

Supplement: Movie S1. Time Lapse Showing Two Phases of Directional Hepatoblast Migration during Liver Bud Formation, Related to Figure 1 [file mmc2.jpg]

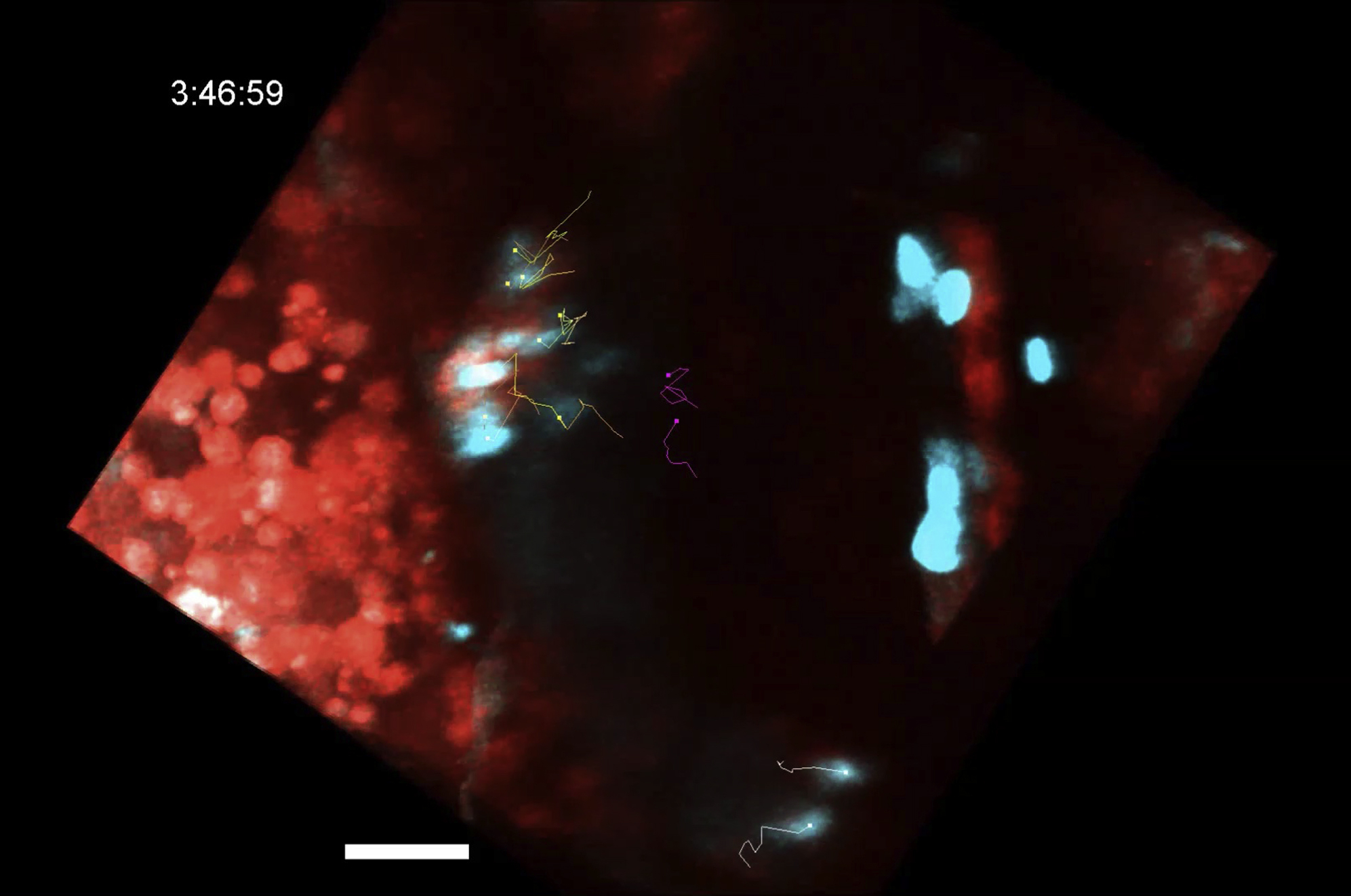

Supplement: Movie S2. Sparse Labeling of Hepatoblasts Reveals Two Phases of Directional Migration during Liver Bud Formation, Related to Figure 1 [file mmc3.jpg]

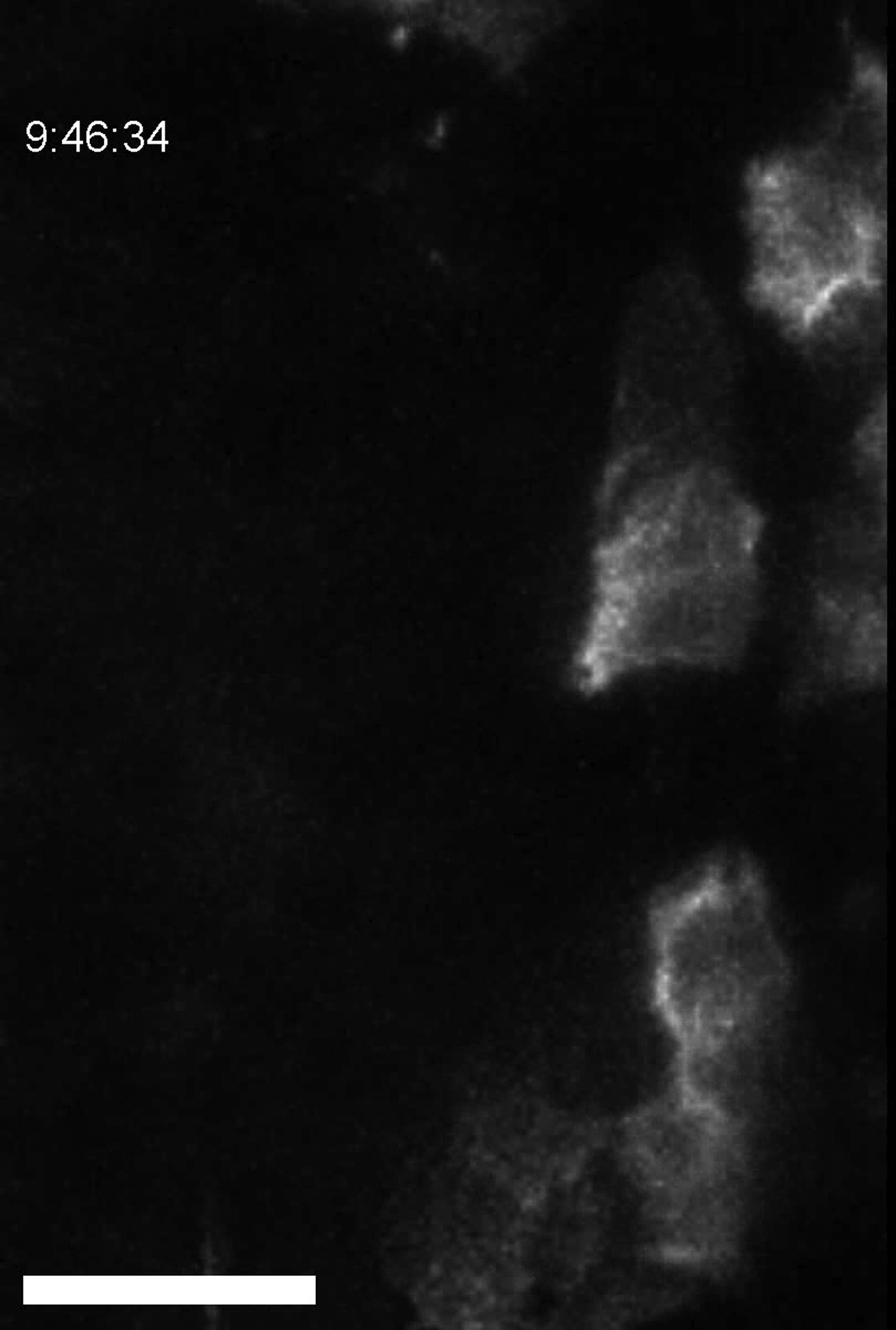

Supplement: Movie S3. Migrating Hepatoblasts Form Filopodia- and Lamellipodia-Like Protrusions during Budding and the Onset of Outgrowth, Related to Figure 2 [file mmc4.jpg]

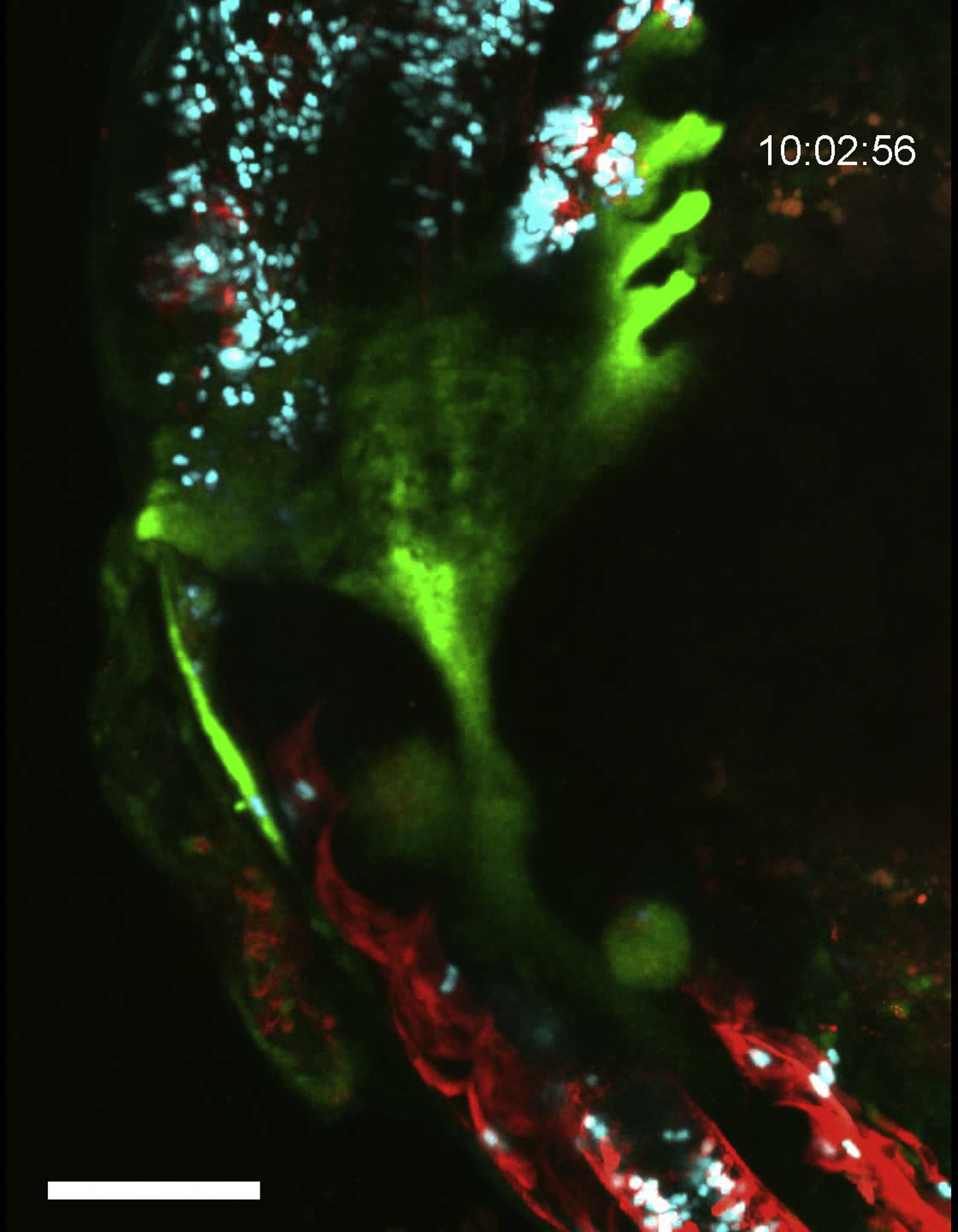

Supplement: Movie S4. Foregut Organ Formation Proceeds Normally during Live Imaging, Related to Figure 1 [file mmc5.jpg]
