# Supplementary material for: EphrinB1/EphB3b Coordinate Bidirectional Epithelial-Mesenchymal Interactions Controlling Liver Morphogenesis and Laterality
Source: Dev Cell. 2016 Nov 7;39(3):316–28. doi: 10.1016/j.devcel.2016.10.009 (PMC5107609; doi:10.1016/j.devcel.2016.10.009)
Supplement: Document S2. Article plus Supplemental Information [file mmc6.pdf]

Developmental Cell

Article

EphrinB1/EphB3b Coordinate Bidirectional Epithelial-Mesenchymal Interactions Controlling Liver Morphogenesis and Laterality

Graphical Abstract

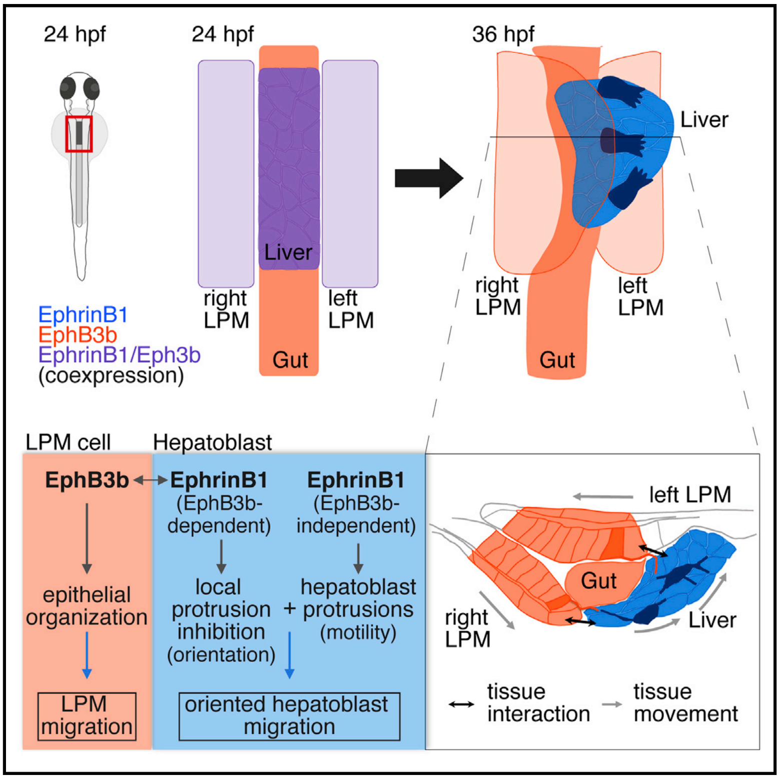

Highlights

- Cell-shape changes and oriented hepatoblast migration mediate asymmetric liver budding
- Bidirectional EphrinB1/EphB3b signaling coordinates liver and LPM movements
- Hepatoblast and LPM protrusions include long-distance connections to each other
- EphB3b-dependent and -independent EphrinB1 functions regulate hepatoblast migration

Authors

Jordi Cayuso, Aliaksandr Dzementsei, Johanna C. Fischer, ..., Josefin Bartholdson, Gavin J. Wright, Elke A. Ober

Correspondence

elke.ober@sund.ku.dk

In Brief

The mechanisms controlling the asymmetric positioning of visceral organs are largely unknown. Cayuso et al. show that bidirectional EphrinB1/EphB3b signaling during liver positioning in zebrafish coordinates active directional hepatoblast migration with the movements of the adjacent lateral plate mesoderm. Long-distance cell-cell interaction between hepatoblasts and mesoderm is mediated via filopodia.

Cayuso et al., 2016, Developmental Cell *39*, 316–328
November 7, 2016 © 2016 Published by Elsevier Inc.
http://dx.doi.org/10.1016/j.devcel.2016.10.009

Developmental Cell
Article

EphrinB1/EphB3b Coordinate Bidirectional Epithelial-Mesenchymal Interactions Controlling Liver Morphogenesis and Laterality

Jordi Cayuso,[1] Aliaksandr Dzementsei,[2] Johanna C. Fischer,[1] Gopal Karemore,[3] Sara Caviglia,[2] Josefin Bartholdson,[4] Gavin J. Wright,[4] and Elke A. Ober[1,2,5,*]
[1]Division of Developmental Biology, Mill Hill Laboratories, The Francis Crick Institute, London NW7 1AA, UK
[2]Danish Stem Cell Center (DanStem), University of Copenhagen, 2200 Copenhagen N, Denmark
[3]Novo Nordisk Foundation Center for Protein Research, Protein Imaging Platform, University of Copenhagen, 2200 Copenhagen N, Denmark
[4]Wellcome Trust Sanger Institute, Cell Surface Signalling Laboratory, Cambridge CB10 1HH, UK
[5]Lead Contact
*Correspondence: elke.ober@sund.ku.dk
http://dx.doi.org/10.1016/j.devcel.2016.10.009

SUMMARY

Positioning organs in the body often requires the movement of multiple tissues, yet the molecular and cellular mechanisms coordinating such movements are largely unknown. Here, we show that bidirectional signaling between EphrinB1 and EphB3b coordinates the movements of the hepatic endoderm and adjacent lateral plate mesoderm (LPM), resulting in asymmetric positioning of the zebrafish liver. EphrinB1 in hepatoblasts regulates directional migration and mediates interactions with the LPM, where EphB3b controls polarity and movement of the LPM. EphB3b in the LPM concomitantly repels hepatoblasts to move leftward into the liver bud. Cellular protrusions controlled by Eph/Ephrin signaling mediate hepatoblast motility and long-distance cell-cell contacts with the LPM beyond immediate tissue interfaces. Mechanistically, intracellular EphrinB1 domains mediate EphB3b-independent hepatoblast extension formation, while EpB3b interactions cause their destabilization. We propose that bidirectional short- and long-distance cell interactions between epithelial and mesenchyme-like tissues coordinate liver bud formation and laterality via cell repulsion.

INTRODUCTION

Complex cell rearrangements are a fundamental feature of embryonic development, converting patterning information into organs and embryos of distinct shapes, sizes, and organization. Great progress has been made in unraveling how single cells and groups of cells move, whereas it is largely unknown how the movement of multiple tissues is coordinated. In the digestive system, the progenitors of the foregut and its accessory organs, the lungs, liver, and pancreas, are specified from a pool of foregut endoderm cells (Zorn and Wells, 2007). These progenitor populations rearrange to form organ buds in stereotypic positions along the alimentary canal, establishing the foundation of the adult organs. Asymmetric positioning of the majority of the visceral organs, including the liver and pancreas is required for their compact packing within the abdominal cavity. Seminal studies in mouse and zebrafish suggest that migration or asymmetric cell rearrangement of adjacent mesodermal tissues have essential roles in the left-right placement of the endodermal organ progenitors (Davis et al., 2008; Horne-Badovinac, 2003). This highlights a fundamental question in development: How are complex morphogenetic movements of multiple tissues coordinated at the cellular and the molecular level?

In zebrafish, the liver progenitors, or hepatoblasts, are specified in the ventral foregut by signals secreted from the adjacent lateral plate mesoderm (LPM) (Chung et al., 2008; Ober et al., 2006; Poulain and Ober, 2011; Shin et al., 2012). Hepatoblasts are initially located symmetrically at the embryonic midline and form shortly after specification an organ bud left of the midline (Field et al., 2003) (Figures 1A–1D). Several transcriptional regulators expressed in hepatoblasts, including Hhex and Prox1, have been associated with different aspects of early liver outgrowth, such as cell proliferation, adhesion, and basal lamina remodeling (Bort et al., 2006; Lüdtke et al., 2009; Margagliotti et al., 2007; Sosa-Pineda et al., 2000; Wallace et al., 2001). To date, there is little evidence for active hepatoblast movements in liver budding (Bort et al., 2006; Klein et al., 2011), whereas the movement of the bilateral LPM epithelia adjacent to the foregut has been shown to be crucial for leftward hepatoblast positioning (Horne-Badovinac, 2003). Concomitant with leftward gut looping and liver positioning, the left LPM moves dorsal to the endoderm, while the right LPM moves ventrolaterally toward the endoderm (Figures 4A‴–4B‴). Mutants with disrupted LPM epithelial morphology or impaired ECM degradation show defective LPM movement and midline-positioned gut and liver, which led to the model that active LPM movements, in particular of the right LPM, exert a motive force on the passive endodermal progenitors directing leftward gut looping and liver outgrowth (Hochgreb-Hagele et al., 2013; Horne-Badovinac, 2003; Yin et al., 2010). How exactly the mesoderm controls this complex morphogenetic rearrangement of the liver progenitors into the liver bud is unclear.

Eph receptor tyrosine kinases and their membrane-tethered Ephrin ligands are divided into two classes: A-type GPI-linked

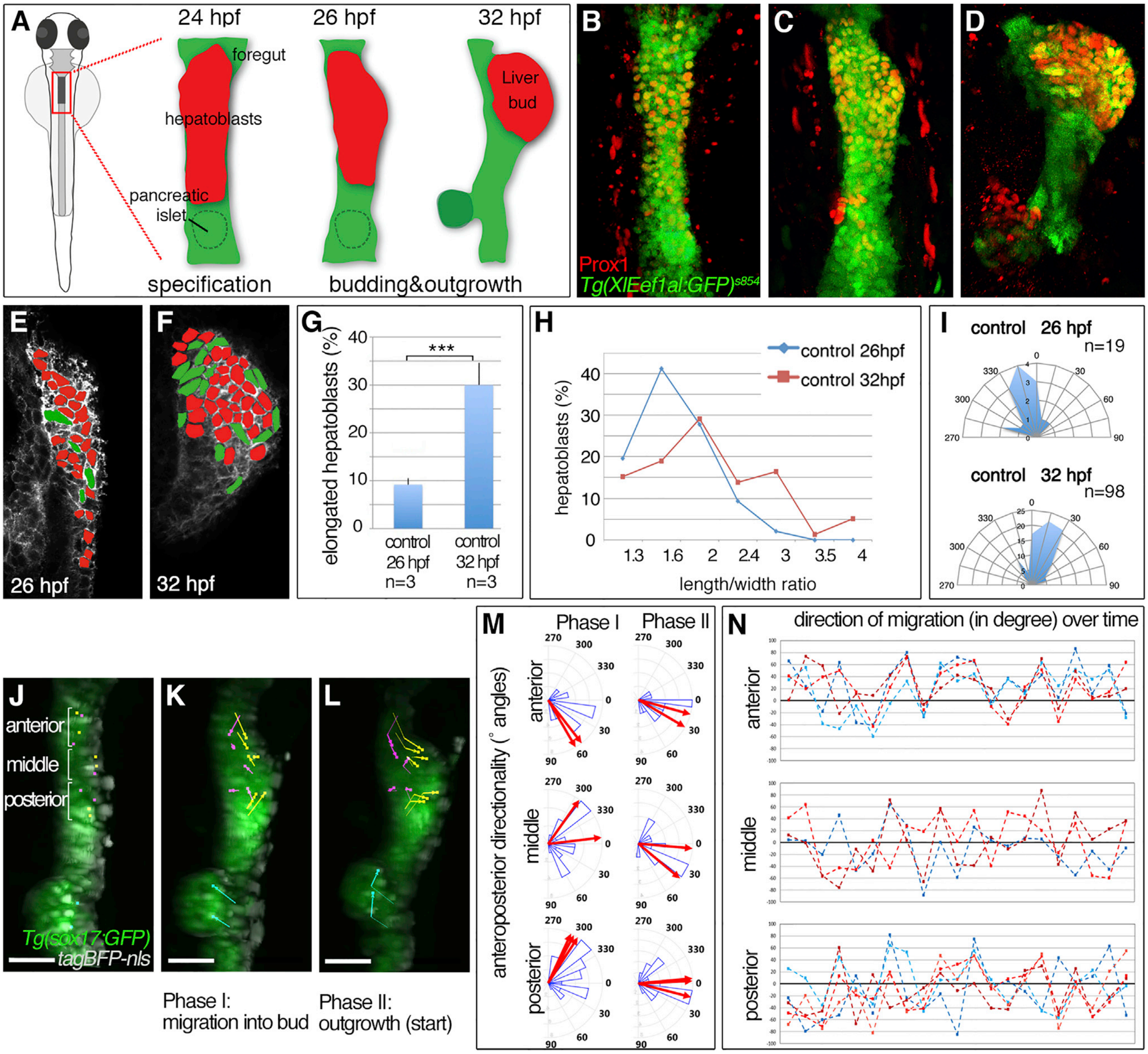

Figure 1. Hepatoblast Polarization Coincides with Liver Budding

(A–D) Stages of liver budding: Schematic (A) and confocal projections of corresponding stages with *Tg(XlEef1a1:GFP)*^{s854} marking the endoderm and Prox1 hepatoblasts; ventral views (B–D).

(E and F) EphrinB1 staining highlights cell shapes at the start of budding (E) and when a bud is apparent (F). Morphometric measurements were performed on serial coronal sections of the bud (E and F); elongated hepatoblasts (L/W $\geq$ 2) are shown in green.

(G–I) Quantification of hepatoblast shape in control embryos at 26 and 32 hpf: (G) proportion of elongated cells per bud; SEs are shown, (H) L/W distribution for one representative bud; (I) orientation of elongated hepatoblasts with respect to the anteroposterior axis.

(J–L) Time lapse of *Tg(sox17:GFP)*-positive foregut starting around 25 hpf (J) shows distinct hepatoblast movements during liver budding (K) and onset of outgrowth (L); dorsal views. TagBFP-nls (gray) marks nuclei for tracking of liver (yellow), gut (magenta), and pancreas progenitors (cyan).

(M and N) Hepatoblasts from different anteroposterior positions migrate with distinct orientation. (M) Rose plots show the distribution of angular displacement with respect to the embryonic midline for 28 min intervals (blue sectors) and the angle of mean displacement per cell for the entire period (red arrow). (N) Line plots representing directionality of displacement over time show individual angular cell displacement for various liver (red hues) and gut progenitors (blue hues).

Scale bars represent 40 μm. ***$p < 0.001$. See also Figure S1; Movies S1, S2, and S4.

Ephrin ligands interact primarily with EphA receptor tyrosine kinases, and conversely B-type transmembrane EphrinB ligands interact predominantly with EphB receptors (Kania and Klein, 2016). A unique property of Eph/Ephrin interactions is the bidirectional activation of signaling. The *trans*-interaction of Ephrin and Eph from adjacent cells initiates forward signaling in Eph-expressing cells and reverse signaling downstream of Ephrins. However, ligand and receptor expression in the same cell can

result in *cis*-interactions that interfere with forward and reverse signaling (Yaron and Sprinzak, 2012). Eph/Ephrin signaling regulates a great variety of cell behaviors, including cell adhesion, shape changes, and migration, important for diverse morphogenetic processes during embryonic development and tissue homeostasis (Kania and Klein, 2016; Pasquale, 2005). Therefore, members of the Eph and Ephrin families represent attractive candidates for controlling the morphogenetic events driving leftward liver outgrowth. Intriguingly, hepatic *EphrinB1* expression has been observed in several vertebrates (Costa et al., 2003; Fletcher et al., 1994; Thisse and Thisse, 2005), whereas its function and an interacting Eph receptor in this context are unknown.

Here, we show that bidirectionally coordinated endoderm and mesoderm movements are crucial for liver bud morphogenesis within the embryo. Contrary to previous models, we show that active hepatoblast migration is essential for liver bud formation and positioning. We identify EphrinB1 and the receptor EphB3b as key factors coordinating the interlinked morphogenetic behaviors of the hepatic endoderm and adjacent LPM, essential for directional liver outgrowth. Mechanistically, we show that EphB3b-independent EphrinB1 function controls hepatoblast protrusion formation, while asymmetric expression of EphB3b in the right LPM triggers EphrinB1-mediated repulsive activity that provides instructive directional cues for mediating asymmetric liver morphogenesis.

RESULTS

Hepatoblasts Actively Migrate during Liver Budding

To determine whether hepatoblasts rearrange actively or are passively displaced during liver budding, we examined their cell behaviors by first assessing cell shapes. Hepatoblasts were outlined by immunolabeling against the transmembrane protein EphrinB1 (Figures 1E and 1F; EphrinB1 expression is described in detail later). We determined the length/width (L/W) ratio of hepatoblasts in coronal and transverse sections at two time points: at 26 hr post fertilization (hpf), the onset of budding when the first hepatoblasts are found left of the midline; and at 32 hpf, when an organ bud has formed and outgrowth is still ongoing (Field et al., 2003). These analyses revealed significant cell-shape changes over time: in coronal sections only 9.2% of all hepatoblasts were elongated (L/W $\geq$ 2) at 26 hpf, while at 32 hpf this population increased dramatically, comprising 30% (Figures 1E–1G). Concurrently, the overall hepatoblast L/W ratio increases significantly (Figure 1H). During budding, elongated cells were predominantly oriented in a 0°–30° angle with respect to the anteroposterior axis (Figure 1I), consistent with directional, anterior-leftward hepatoblast outgrowth. Cell-shape analysis in transverse sections (encompassing dorsoventral and mediolateral axes) revealed no difference in hepatoblast elongation at 26 and 32 hpf (Figure S1), suggesting cell polarization along the anteroposterior axis. In contrast to previous models, in which gut looping and liver positioning are solely the result of asymmetric LPM migration and passive hepatoblast displacement (Hochgreb-Hagele et al., 2013; Horne-Badovinac, 2003; Yin et al., 2010), these shape changes indicate oriented hepatoblast movement during budding.

To further validate our hypothesis, we followed hepatoblast movement in the embryo using time-lapse confocal microscopy during budding, between 24 and 36 hpf. Tracking of fluorescently labeled nuclei revealed that hepatoblasts move in a coordinated fashion and neighbor exchange occurs between hepatoblasts, suggesting active collective cell migration (Figures 1J–1N and S1F–S1H, Movies S1 and S2). First, hepatoblasts move directionally to aggregate into the liver bud, with anterior hepatoblasts moving posterior leftward, posterior ones moving anterior-leftward, and the intermediate population just leftward (Figures 1K and 1M). This is followed by a second phase where hepatoblasts move more directionally to the left with a more consistent angular displacement (Figures 1L and 1M). In contrast, other endodermal populations exhibit distinct motile behaviors, such as future gut cells, which initially reside at the same anteroposterior position of the endodermal rod, but undergo smaller displacements with clear differences in the direction of movement (Figures S1I and S1J, Movies S1 and S2) and pancreatic cells which move in the opposite direction, anterior-right (Figures 1J–1M). Although hepatoblasts from the same area migrate in the same overall direction, individual cells display different directionality relative to each other at a given time point, corroborating active migration (Figure 1N). Together, these data show that hepatoblasts actively migrate during liver budding.

Hepatoblasts Form Lamellipodia- and Filopodia-like Protrusions

Our cell-shape and time-lapse analyses suggest that hepatoblasts actively move during liver budding. To corroborate this finding, we examined hepatoblast morphology at greater resolution by expressing membrane-tethered fluorescent proteins in small clones in the forming liver. This analysis revealed that wild-type hepatoblasts as well as LPM cells exhibit unexpectedly elaborate morphologies, including distinct cellular protrusions (Figure 2). We identified two major protrusion types: flat, sheet-like protrusions resembling lamellipodia (Figures 2A and 2A′), and thin filopodia-like extensions, a subset of which is branched (Figures 2B and 2B′). Filopodia and lamellipodia are F-actin-rich structures (Ridley, 2011). To examine the distribution of cellular actin in clones, we expressed GFP-tagged Utrophin, a protein associating with F-actin without interfering with its function (Burkel et al., 2007). Labeled cells showed an enrichment of GFP close to the cell membrane corresponding with cortical actin, as well as in hepatoblast and LPM protrusions (Figures 2C and 2C′), supporting their classification as filopodia- and lamellipodia-like extensions.

Hepatoblasts form filopodia-like extensions that are on average 3.4 μm long, and can reach up to 13.6 μm (equivalent to ~2 cell diameters). Similarly, epithelial LPM cells form basal protrusions, which are on average 7.2 μm and up to 26.5 μm long. These extensions interconnect both tissues, as they frequently extend from LPM clones into the hepatic domain making contacts with hepatoblasts away from the tissue border and from hepatoblasts to the border of the LPM (Figures 2B and 2B′). These findings indicate that both tissues form direct physical contacts not only at the hepatoblast/LPM interface, but also long-distance cell-cell interactions.

Each protrusion type contributes to complex cellular behaviors, with filopodia exploring and sensing the environment and lamellipodia mediating movement (Ridley, 2011). To elucidate hepatoblast behaviors during budding, protrusions were

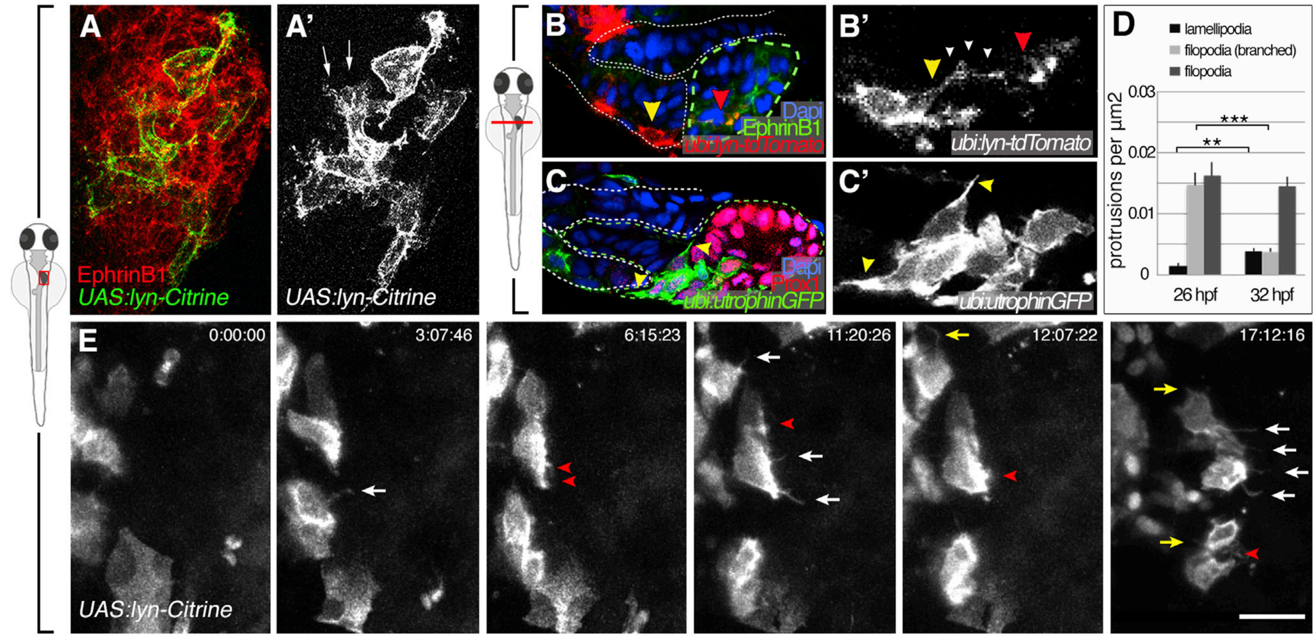

Figure 2. Hepatoblasts Form Filopodia- and Lamellipodia-like Protrusions during Liver Budding

(A–B′) Mosaic *UAS:lyn-citrine* or *ubi:lyn-tdTomato* expression shows EphrinB1$^+$ hepatoblasts form lamellipodia (arrows in A′) and filopodia-like extensions (B and B′). Extensions (white arrowheads) connect the LPM (yellow arrowhead) and hepatoblasts (red arrowhead) over several cell diameters (B and B′). Dashed lines outline the LPM (white) and endoderm (green).

(C and C′) Utrophin-GFP highlights actin in the cortical network and protrusions of hepatoblasts (arrowheads). Dashed lines delineate endoderm (green) and LPM (white).

(D) Quantification of hepatoblast protrusions shows an increase of lamellipodia and decrease of filopodia-like extensions during budding.

(E) Time lapse of migrating hepatoblasts during liver budding and early outgrowth; dorsal views. Membrane labeling with *UAS:lyn-Citrine* shows filopodia in the direction of outgrowth (white arrows) and toward the LPM (yellow arrows) and lamellipodia (red arrowheads); stills of Movie S3. Scale bars represent 30 µm. $^{**}p < 0.01$, $^{***}p < 0.001$). See also Movie S3.

quantified between 26 and 32 hpf. Hepatoblasts form about the same number of simple filopodia-like protrusions at both stages, while the number of branched filopodia-like protrusions decreases by 75% and lamellipodia formation dramatically increases by 163% at 32 hpf (Figure 2D), indicating a significant shift from predominantly sensing to more motile cell behaviors during budding. This protrusive activity was corroborated by live imaging, showing filopodia and lamellipodia-like protrusions dynamically extending in the direction of migration, as well as some toward the midline and LPM (Figure 2E and Movie S3).

Cell Protrusions Are Important for Hepatoblast Positioning

To investigate the functional relevance of these cell extensions in liver bud morphogenesis, *Tg(XlEef1a1:GFP)^{s854}* embryos with GFP highlighting the endoderm were incubated during early budding stages with the F-actin-depolymerizing drug Latrunculin B (Lat B). Given the importance of actin polymerization for numerous cellular processes, we minimized the exposure to Lat B and treated the embryos from 26–32 hpf with a low dose of the drug (0.1 µg/ml). This treatment resulted in a 33% decrease in the number of hepatoblast protrusions at 32 hpf (Figures 3A–3C), and ectopic Prox1-positive hepatoblasts in posterior positions leading to a 20% increase of the anteroposterior extent of the Prox1 domain compared with DMSO-treated controls (Figures 3D–3F). In addition, hepatoblasts resided at or close to the midline, suggesting that oriented anterior-leftward hepatoblast migration is compromised.

To determine the cell autonomous functions of protrusions in liver budding, we manipulated the small Rho GTPase Cdc42, a well-known regulator of filopodia formation (Nobes and Hall, 1995). Mosaic expression of the dominant-negative Cdc42^{T17N} (Nalbant et al., 2004) at 26 hpf resulted in a significant reduction of hepatoblast protrusions at 32 hpf (Figures 3G–3I), which is accompanied by a significant increase of the anteroposterior extent of the Prox1 domain (Figures 3J and 3K). In contrast, wild-type Cdc42 overexpression alters neither protrusion number (Figures 3I and 3K) nor the length of the Prox1 domain.

Altogether, these findings support the importance of cellular extensions for hepatoblast movement in liver budding.

EphrinB1 and EphB3b Expression Is Dynamic and Complementary in the Liver-Forming Foregut Domain

Searching for factors that mediate hepatoblast movement, we identified *ephrinb1* expression in the liver domain during budding stages (Figure S2C). EphrinB1 represented an excellent candidate, since Ephrin ligands and their Eph receptors can control cytoskeletal dynamics and thereby diverse morphogenetic processes (Kania and Klein, 2016). Given that EphrinB1 signaling is generally activated by interaction with EphB receptors, we searched for one expressed in the foregut area and identified the EphB3 homolog *ephb3b* (Figure S2D). In order to examine

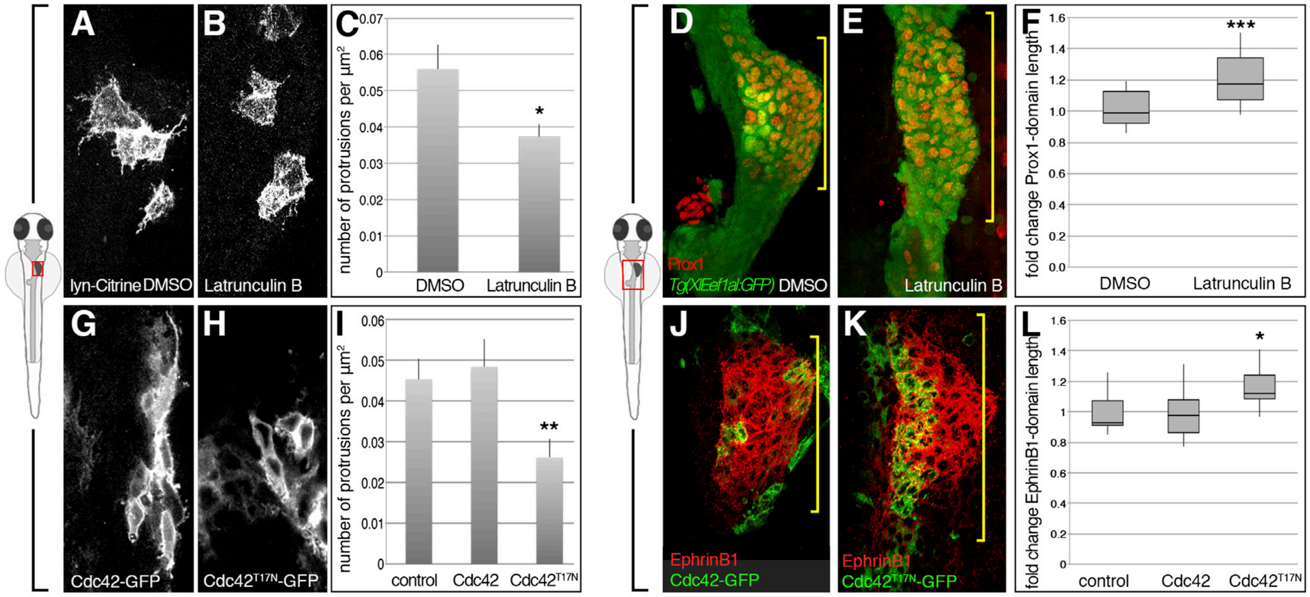

Figure 3. Compromised Protrusion Formation Correlates with Liver Budding Defects

(A–F) Latrunculin B (0.1 µg/ml) treatment during liver budding (26–32 hpf) leads to significantly less hepatoblast protrusions (A–C; DMSO clones, n = 7; Lat B clones, n = 9, N = 1), and a significantly longer Prox1 domain (bracket) (D–F; DMSO, n = 23; Lat B, n = 13, N = 2).

(G–L) Hepatoblasts expressing Cdc42^{T17N}-GFP during liver budding (26–32 hpf) form significantly less protrusions compared to controls and Cdc42-GFP (G–I; control clones, n = 10; Cdc42-GFP clones, n = 16; Cdc42^{T17N}-GFP clones, n = 18, N = 1) and a significantly longer EphrinB1 domain (bracket) (J–L; control, n = 7; Cdc42-GFP, n = 18; Cdc42^{T17N}-GFP, n = 16, N = 2). N indicates the number of experiments.

*p < 0.05, **p < 0.01, ***p < 0.001.

EphrinB1 and EphB3b expression at cellular resolution, we generated antibodies against their ectodomains that recapitulate the corresponding mRNA expression (Figures 4A–4B‴ and S2A–S2D). We found that EphrinB1 represents one of the first genes expressed in zebrafish liver precursors, starting around 22 hpf, which corresponds with the onset of previously described hepatoblast gene expression (Ober et al., 2006). At this stage, EphB3b is co-expressed with EphrinB1 in the hepatic endoderm and adjacent LPM, while, in the gut, solely EphB3b is detected (Figures 4A–4A‴). From 26 hpf, with the onset of liver budding, ligand and receptor expression become complementary: EphrinB1 is expressed in hepatoblasts, while EphB3b is present in the LPM and restricted gut domains anterior and posterior to the liver anlage (Figures 4B–4B‴). Altogether, EphrinB1 and EphB3b interaction interfaces are established between hepatoblasts and the adjacent LPM during liver budding.

EphrinB1 and EphB3b Control Early Liver Bud Morphogenesis

To determine the role of EphrinB1 and EphB3b in liver organogenesis, and the functional significance of the hepatoblast-LPM interactions, we used morpholino antisense oligonucleotide (MO)-mediated knockdown (Figures S2E–S2J′). In *ephrinB1* or *ephB3b* morphant embryos, liver specification is indistinguishable from controls at 25 hpf (Figures S2K–S2N), whereas hepatoblast positioning and bud formation are defective in 88% of *ephrinB1* morphants (n = 140) and 86% of *ephB3b* morphants (n = 211) at 32 hpf (Figures 4C–4E and S2X–S2Z). Similar defects were observed upon transgenic expression of EphrinB1^{EC}, a dominant-negative form encompassing the extracellular domain of EphrinB1 (Holder and Klein, 1999), confirming a requirement for EphrinB/EphB signaling in liver morphogenesis (Figures 4F–4F‴). In embryos with impaired EphrinB1 or EphB3b function, ectopic Prox1-positive hepatoblasts reside in more posterior and medial positions (Figures 4D–4F″ and S2X–S2Z), causing subsequent liver morphology defects at 54 hpf, with altered extrahepatic duct formation and liver tissue located closer to or ectopically at the midline (Figures S2O–S2W′). In morphant embryos, liver budding is more severely disrupted by EphB3b knockdown, with frequently bilaterally located hepatoblasts (Figures 4E–4E‴ and S2V–S2V′). To test whether the difference in phenotype might be due to incomplete EphrinB1 knockdown, we generated a genetic mutant using the Crispr/Cas9 system. *ephrinb1^{nim26}* mutants exhibit ectopic Prox1-positive hepatoblasts with 90% showing an elongated Prox1 domain at 32 hpf (n = 16; Figures S2Bb–S2Bb″). *ephrinb1* mutant and morphant embryos therefore show a similar phenotype and, unlike in EphB3b morphants, hepatoblasts are generally positioned to the left and not across the midline. This indicates that the EphrinB1 and EphB3b knockdown phenotypes are specific and not due to incomplete knockdown of EphrinB1. Moreover, the number of Prox1-positive cells is not altered in *ephrinB1* and *ephB3b* morphants (Figure S2Aa), indicating that the domain expansion is due to morphogenesis defects and not increased proliferation or ectopically specified progenitors.

EphrinB1 and EphB3b Regulate Hepatoblast Cell-Shape Changes and Epithelial LPM Organization

Ephrins/Ephs are important regulators of tissue morphogenesis, including polarization and oriented migration of cells

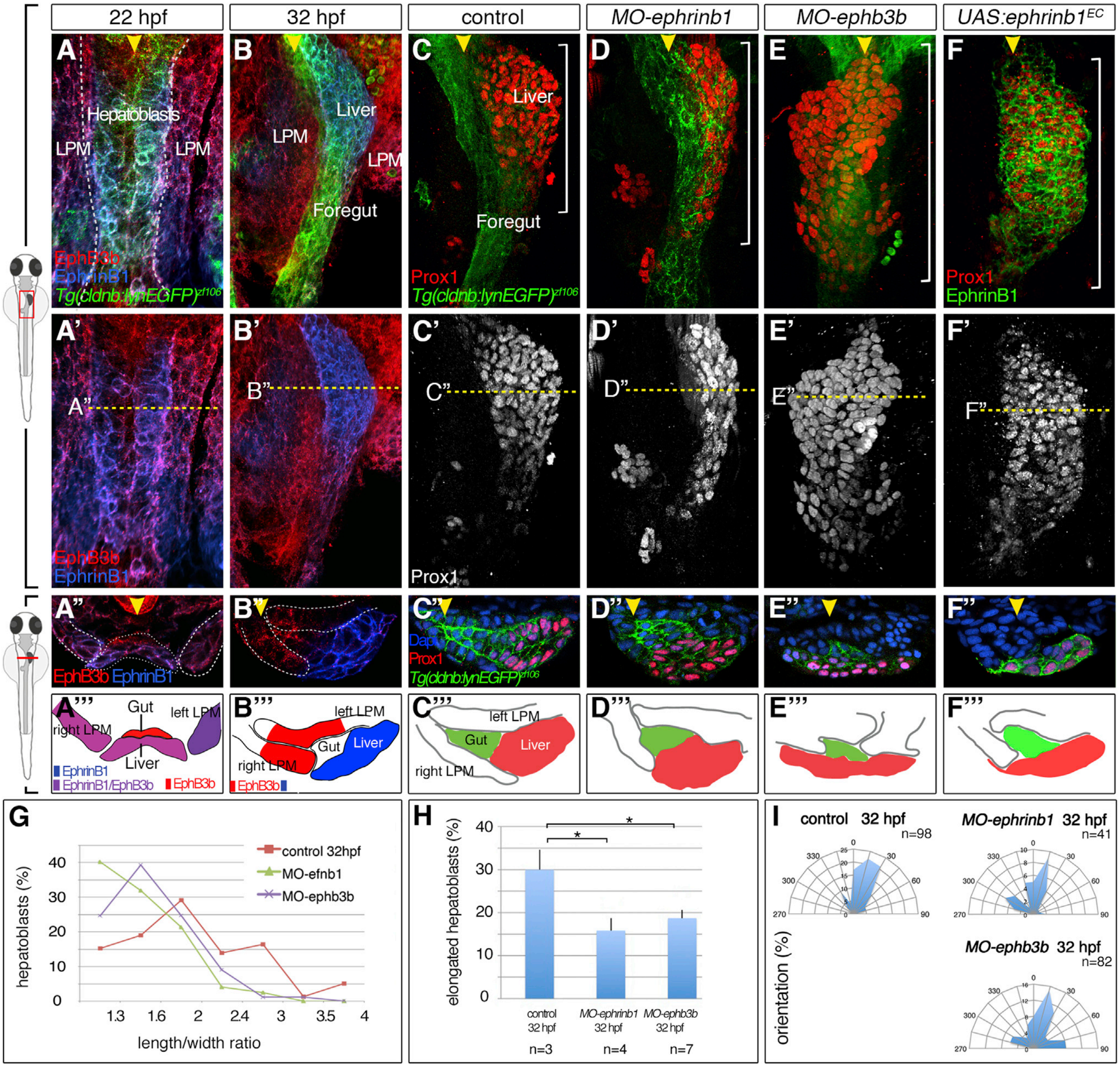

Figure 4. Complementary EphrinB1 and EphB3b Expression Controls Hepatoblast Positioning and Cell-Shape Changes in Liver Bud Formation

(A–F‴) At 22 hpf, EphrinB1 and EphB3b expression largely overlaps in future hepatoblasts and the LPM (A–A‴). Complementary expression of both factors coincides with the start of liver budding: EphrinB1 in hepatoblasts and EphB3b in the gut and LPM (B–B‴). At 32 hpf, hepatoblasts are located more posteriorly and medially compared with controls in *MO-ephrinb1* (C–D‴), in *MO-ephb3b* (E–E‴), and upon conditional *UAS:ephrinb1*^{EC} expression (F–F‴). (A–F′) ventral views of confocal projections, anterior to the top; (A″–F″) transverse sections of the foregut, as indicated by the dashed line in (A′–F′), and matching schematics (A‴–F‴); yellow arrowheads specify the midline and white brackets the length of the Prox1 domain.

(G–I) Cell shapes were determined with EphrinB1-staining at 32 hpf (see Figure 1F). Quantification of hepatoblast shape in control, *MO-ephrinb1*, and *MO-ephb3b* embryos: (G) L/W distribution for one representative bud; (H) proportion of elongated cells per bud; SEs are shown; and (I) orientation of elongated hepatoblasts with respect to the anteroposterior axis. *$p < 0.05$.

See also Figure S2.

(Pasquale, 2005; Poliakov et al., 2004). To ascertain whether EphrinB1 and EphB3b regulate liver morphogenesis by controlling cell polarity and migration during budding, we analyzed hepatoblast morphologies at 32 hpf. Coronal sections showed 47% and 37% fewer elongated hepatoblasts in *ephrinB1* or *ephb3b* morphants, respectively, displaying significantly reduced L/W ratios and altered orientations with respect to the midline (Figures 4G–4I). These findings suggest that

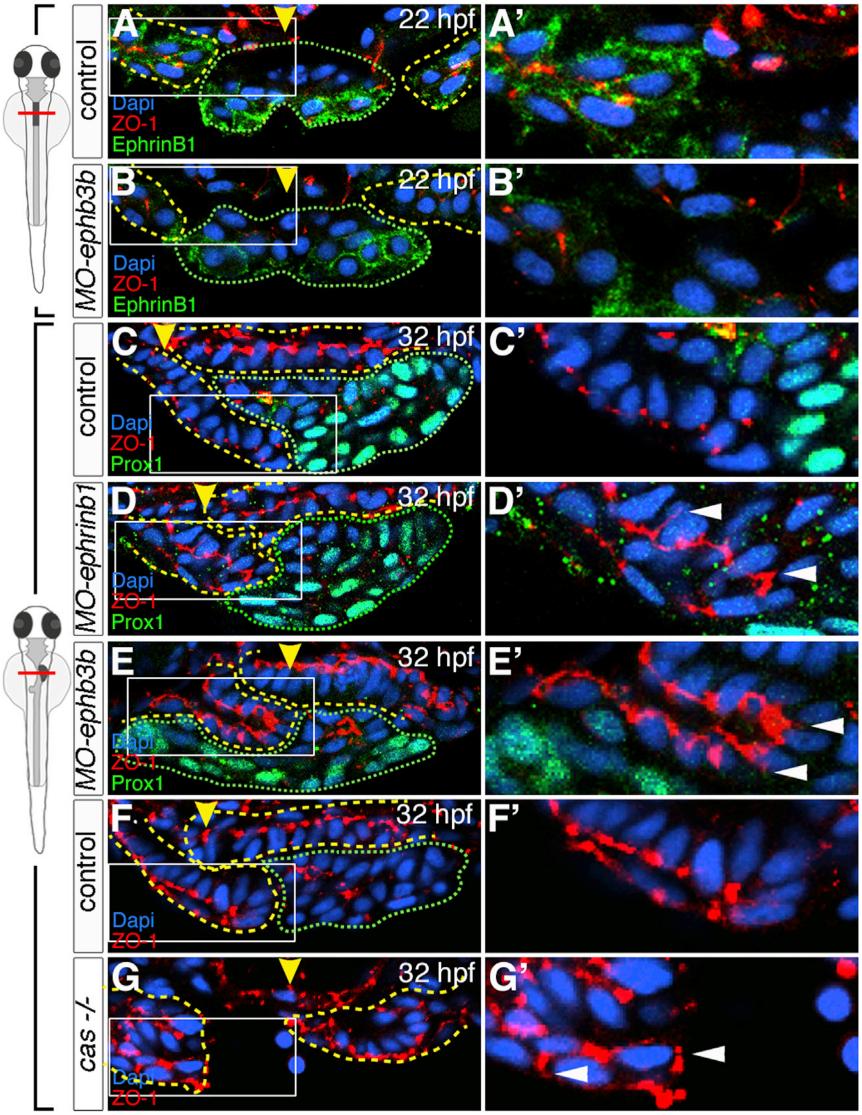

Figure 5. EphB3b Controls LPM Polarity and Asymmetric Movement

(A–E′) EphrinB1 and EphB3b regulate LPM polarity in the foregut region. α-ZO-1 staining in *MO-ephb3b* embryos reveals that junctions in the LPM form similar to controls at 22 hpf (A–B′); while at 32 hpf, ZO-1 is mislocalized (arrowheads) in *MO-ephrinb1* and *MO-ephb3b* embryos (C–E′). Yellow arrowheads specify the midline and reveal impaired gut looping in *MO-ephrinb1* and *MO-ephb3b* (C–E).

(F–G′) *casanova* mutants exhibit ZO-1 localization defects in the LPM (arrowheads) at 32 hpf.

(A–G′) Transverse sections at liver level, left side to the right; dashed lines delineate the endoderm (green) and LPM (yellow); (A′–G′) are magnifications of the areas indicated by a box in (A–G).

EphrinB1 and EphB3b regulate hepatoblast rearrangement during budding.

Since EphrinBs and EphBs represent bidirectional signaling pairs, we analyzed the morphology of the EphB3b-expressing LPM and its behaviors. Polarization of the LPM epithelia is key for asymmetric LPM migration, which directs leftward gut looping and liver positioning (Horne-Badovinac, 2003). In *ephrinb1* and *ephb3b* morphants, gut looping was impaired, with more pronounced defects in the latter (Figures 4D–4E‴ and 5C–5E′). Consistent with more severe defects in *ephb3b* morphants, medially positioned guts were accompanied by symmetrically placed left and right LPM, mostly dorsal to the endoderm (Figures 4E″, 5E, and 5E′). To visualize LPM cell polarity in morphants, we analyzed Zonula occludens1 (ZO-1) localization marking tight junctions. In *ephrinb1*-and *ephb3b*-morphant LPMs, tight junctions were established initially at 22 hpf (Figures 5A–5B′), however, their localization was subsequently disrupted and apical ZO-1 frequently expanded basally at 32 hpf (Figures 5C–5E′). Hence, both EphrinB1 and EphB3b are required to maintain LPM epithelial organization and associated asymmetric LPM movement, likely through activation of forward signaling downstream of EphB3b in the LPM.

These findings also implicate that signals from the endoderm to the mesoderm are important for mesoderm morphogenesis. To further explore the role of the endoderm in this process, we performed ZO-1 stainings in endoderm-less *casanova/sox32* mutants. This revealed severe defects in LPM polarity (Figures 5F–5G′), confirming the crucial function of the endoderm and derived signals in LPM organization.

EphrinB1 Controls Hepatoblast Protrusions by EphB3b-Independent and -Dependent Functions

Given that EphrinB1 and EphB3b control cell-shape changes and polarity during liver bud morphogenesis, and the similarity of the *MO-ephrinb1* and *MO-ephb3b* liver budding phenotypes to those following Lat B treatment, we decided to examine the role of EphrinB1 and EphB3b in hepatoblast protrusion formation. Using the sparse labeling strategy to visualize cell membranes, we observed that extension formation was generally impaired in *ephrinB1* morphants, resulting in fewer and shorter protrusions (Figures 6A, 6B, and 6G), whereas EphrinB1 overexpression significantly increases protrusion formation (Figures 6D, 6G, and S3A–S3B″, Table S1). In contrast, knockdown of EphB3b resulted in an unexpected and striking increase in branched filopodia-like protrusions (Figures 6A, 6C, and 6G), indicating that both factors seem to have opposite effects on protrusion formation. Furthermore, when examining the direction of these protrusions with respect to the anteroposterior axis, we found that lamellipodia in control or *MO-ephrinb1* embryos are preferentially oriented in the direction of hepatoblast movement and bud outgrowth, while lamellipodia orientation in *MO-ephb3b* embryos is randomized (Figure 6H). This indicates that EphrinB1

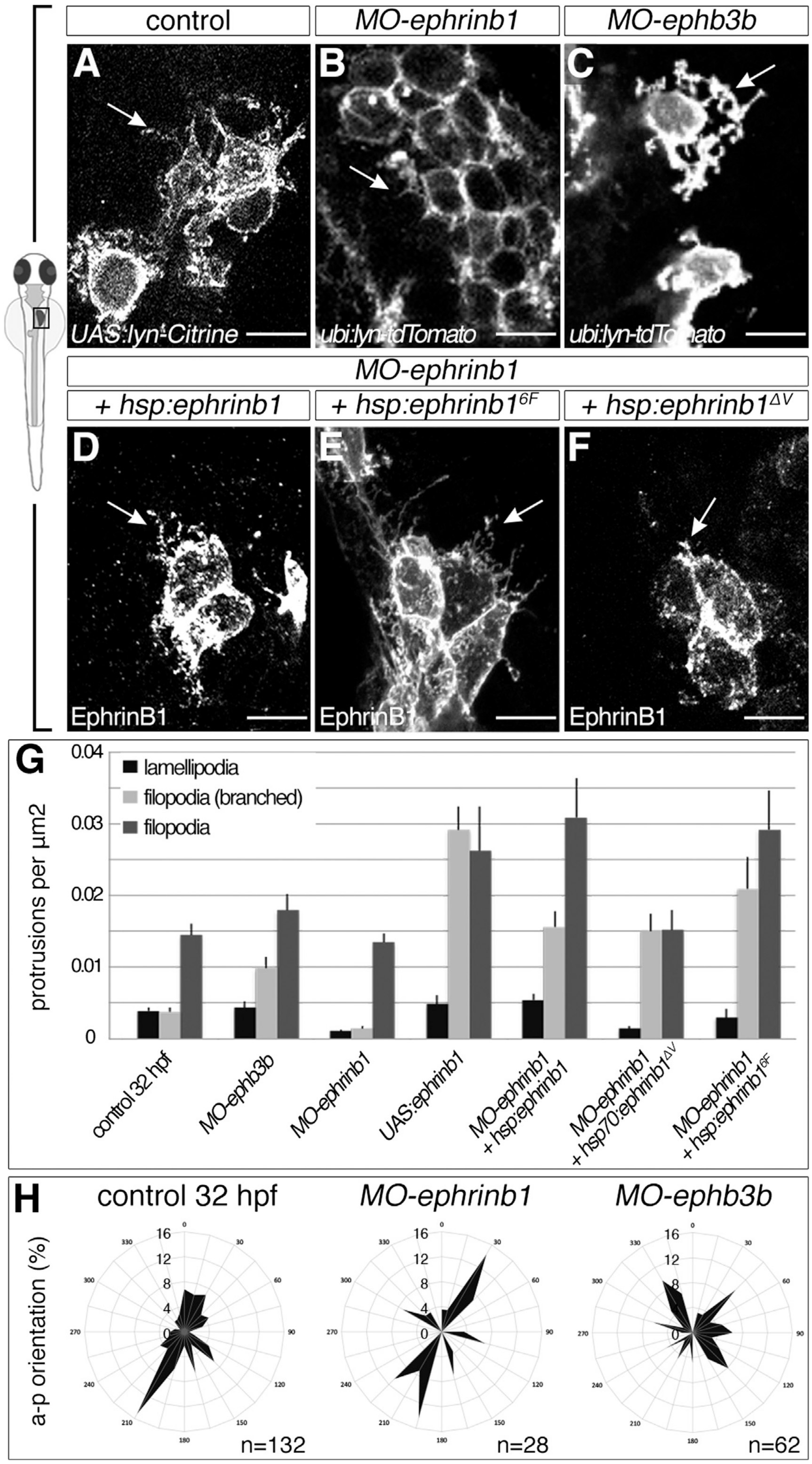

Figure 6. Opposing EphrinB1 and EphB3b Functions Control Formation of Hepatoblast Protrusions

(A–G) EphrinB1 and EphB3b regulate hepatoblast protrusion number and morphology. Sparse labeling reveals fewer and shorter filopodia in *MO-ephrinb1* hepatoblasts than in controls (A and B). Conversely, *MO-ephb3b* hepatoblasts show more complex, branched extensions (C). In contrast to EphrinB1 and EphrinB1^{6F} (D and E), EphrinB1$^{\Delta V}$ fails to rescue extension formation in *MO-ephrinb1* (F). Arrows indicate representative protrusions (A–F). Quantification of hepatoblast protrusion types in various conditions; comparative p values are shown in Table S1 (G).

(H) Hepatoblast lamellipodia orientation is randomized in *MO-ephb3b*.

(A–C) lyn-Citrine and lyn-Tomato outline hepatoblasts, (D–F) EphrinB1 staining highlights overexpression of different forms of EphrinB1, (A–F) ventral projections, anterior to the top. SEs are shown. Scale bars represent 10 μm. See also Figure S3; Table S1.

is required and sufficient to promote protrusion formation in hepatoblasts. Interaction with EphB3b expressed in LPM cells destabilizes filopodia and orients lamellipodia, thus providing spatial information. Hence, *MO-ephrinb1* hepatoblasts exhibit impaired motility, while this is intact in *ephb3b* morphant hepatoblasts, which instead, due to the lack of repulsive directional cues, distribute across the midline, explaining the common and distinct hepatoblast positioning phenotypes in either knockdown. These results also imply that EphrinB1 functions both dependently and independently of EphB3b in hepatoblasts.

Receptor-dependent and -independent EphrinB1 functions are mediated by conserved signaling motifs in the cytoplasmic domain, including six tyrosine phosphorylation sites and a C-terminal PSD-95/discs large/ZO-1 (PDZ)-interacting domain (Bochenek et al., 2010; Cowan and Henkemeyer, 2002). To distinguish domain-specific activities in hepatoblasts, we conditionally expressed full-length EphrinB1 or EphrinB1 mutant proteins in *MO-ephrinb1* hepatoblasts and examined hepatoblast protrusions. Mosaic expression of EphrinB1 or EphrinB1^{6F}, in which phosphotyrosine signaling is impaired, rescues the formation of all protrusion types during liver budding (Figures 6D, 6E, and 6G). In contrast, expression of EphrinB1$^{\Delta V}$, which is unable to interact with PDZ-domain proteins (Davy et al., 2004), failed to restore lamellipodia as well as basic filopodia formation (Figures 6F and 6G, Table S1). Notably, protrusion branching is increased upon expression of the different EphrinB1 proteins, similar to *ephb3b* morphants (Figure 6C and Table S1). This may reflect increased receptor

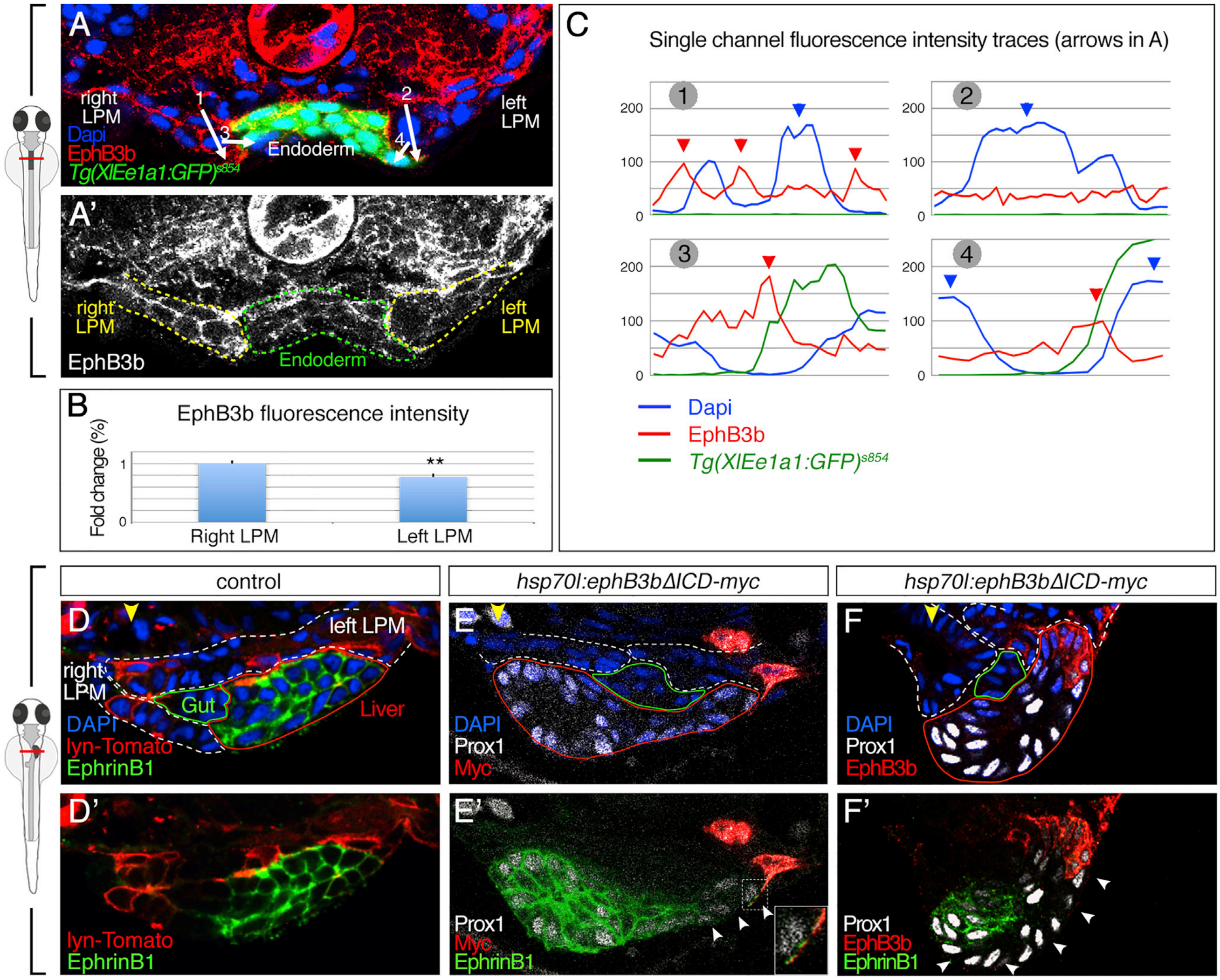

Figure 7. Asymmetric EphB3b Can Exert Repulsive Activity during Liver Budding

(A–C) EphB3b expression is higher on the right than the left LPM at 24 hpf (A and A′). Quantification of EphB3b expression measuring overall fluorescent intensity (B) and intensity profile (C). Fluorescent intensity profiles show high EphB3b at cell membranes (red arrowheads) of the right but not left LPM; numbered arrows in (A) indicate the position of profiles. The right LPM-hepatoblast interface (profile 3) shows highest EphB3b expression. High DAPI levels indicate nuclei position (blue arrowheads).

(D–F) Ectopic EphB3b$^{\Delta ICD}$ expression alters hepatoblast position. Compared with mosaic lyn-Tomato (D), mosaic EphB3b$^{\Delta ICD}$ expression on the left LPM (E) or left LPM and hepatoblasts (F) at 26 hpf causes positioning of Prox1^{+} hepatoblasts away from the clone at 32 hpf. EphrinB1 is absent from membranes in 3–7 hepatoblasts next to EphB3b$^{\Delta ICD}$ clones (white arrowheads), without altering Prox1 expression (E′ and F′). (E′) Inset shows an EphrinB1 signal in EphB3b$^{\Delta ICD}$ protrusion, indicating direct cell interaction. (A, A′ and D–F′) Transverse sections at liver level, left side to the right; yellow arrowhead specifies the midline; lines delineate the left and right LPM (white), gut (green), hepatoblasts (red).

**$p < 0.01$. See also Figures S4 and S5.

internalization, due to possible saturation with ligands (Bush and Soriano, 2010), and is consistent with compromising oriented cell motility (Figure S3C). In summary, our results suggest that EphrinB1 promotes extension formation through its PDZ-binding domain, independent of EphB3b, while, upon receptor interaction, filopodia-like protrusions collapse and lamellipodia re-orient in line with repulsive receptor functions.

A repulsion-based mechanism for leftward liver positioning would call for the asymmetric distribution of repulsive EphB3b to mediate leftward hepatoblast movement. Quantification of EphB3b levels in the LPM at 24 hpf, when the foregut endoderm, including the prospective hepatoblasts, reside at the embryonic midline, revealed a 23% higher level of EphB3b throughout the right LPM compared with the left LPM (n = 9, p = 0.0095; Figures 7A–7C). This difference is more pronounced at the plasma membranes where EphB3b levels are 70% higher at the right LPM-endoderm interphase than on the left (n = 3, p = 0.0197; Figures 7A and 7C). Shortly after, at 26 hpf, when leftward hepatoblast movement is initiated, the average expression of EphB3b is still 21% higher throughout the right LPM compared with the left. EphB3b membrane localization on the left LPM is very low or absent next to leftward migrating hepatoblasts, in line with a

permissive environment for outgrowth. The spatially confined EphB3b expression is maintained and more distinct at 32 hpf (Figures 4B–4B‴). This indicates that differences of EphB3b levels and distribution between the left and the right LPM epithelia are established prior to morphological signs of asymmetric liver outgrowth.

If the LPM provides repulsive EphB3b cues to control hepatoblast positioning, then ectopic expression of EphB3b should redirect hepatoblasts. To test this, we generated a truncated form of EphB3b lacking the intracellular domain, $EphB3b^{\Delta ICD}$, that stimulates reverse signaling upon interaction with EphrinB ligands without eliciting forward signaling (Zimmer et al., 2003). By conditional expression of $EphB3b^{\Delta ICD}$ at 26 hpf, we generated 38 clones in the liver area (38/>200 embryos) with 16 on the left side in domains with no or low endogenous EphB3b. In contrast to controls, in 81% of these embryos (13/16), liver morphology was disrupted with hepatoblasts turning around and moving to the right side, in many cases ventrally to the gut and right LPM (Figures 7D–7F′). This high correlation of altered hepatoblast location and clone position cannot be explained solely by LPM movement defects, because 19% of embryos analyzed at 32hpf (3/16) and 75% analyzed at 28–30 hpf (3/4) with ectopic liver budding still show asymmetric migration of the LPM (data not shown). The gut is mostly in its normal position to the left of the midline, indicating that liver and gut progenitors can move independently from each other and that EphB3b provides directional cues. This is further supported by experiments in which transient injection of *ephrinb1* gRNAs/Cas9 causes mosaic depletion of endogenous EphrinB1 (Figure S4). If EphB3b would not repel EphrinB1-positive hepatoblasts, they should distribute randomly among the EphrinB1-negative cells, as observed in controls in which hepatoblasts mosaically express lyn-Tomato (Figures S4A, S4A′, and S4C′). However, in 9/10 embryos, the EphrinB1-positive hepatoblasts accumulate in the budding area separated from the LPM by 1–6 hepatoblasts expressing no or low level of EphrinB1 (Figure S4). This corroborates that EphrinB1-positive hepatoblasts move away from EphB3b and that they sense EphB3b not only at the direct tissue interface but also over longer distances. Cell interactions away from the tissue interface are further supported by the observation that several cell layers of hepatoblasts next to an $EphB3b^{\Delta ICD}$ clone show no EphrinB1 at the membrane (Figures 7E′–7F′), in line with direct Eph/Ephrin interaction-triggered removal of the complex by endocytosis (Pitulescu and Adams, 2010).

DISCUSSION

Our studies provide functional evidence that liver bud formation and its asymmetric positioning require the coordinated movement of two tissues: the hepatic endoderm and the adjacent LPM (Figure S5). Filopodia-like protrusions extending over several cell diameters create LPM-hepatoblast contacts away from the immediate tissue interface, indicating long-distance interactions. Moreover, we identify EphrinB1 in the liver and EphB3b in the LPM as the bidirectional molecular link orchestrating the interconnected movement of both tissues by regulating hepatoblast motility and orientation, and the differentiation of the highly polarized LPM epithelia critical for LPM migration. We propose that the LPM directs the liver into its position by a repulsion-based mechanism, uncovering an additional mechanism for generating left-right tissue asymmetries.

Previous studies suggested that gut looping and digestive organ asymmetry is the result of actively rearranging and moving mesodermal tissues pushing the passive endoderm into position, including the zebrafish liver (Davis et al., 2008; Hecksher-Sorensen, 2004; Horne-Badovinac, 2003). Our findings indicate that zebrafish hepatoblasts are already in an asymmetric position at a stage before overt signs of gut looping (Figures 7A and 7A′) and leftward liver bud positioning is a consequence of active and coordinated rearrangement of both the LPM and endodermal hepatoblasts. By tracking individual cells during budding, we show that hepatoblasts migrate, forming dynamic filopodia and lamellipodia. Compared with adjacent gut progenitors, hepatoblasts are displaced more directionally and generally over a greater distance, supporting their active role in liver positioning. In addition, the presence of ectopic hepatoblasts at posterior positions in EphrinB1- and EphB3b-depleted embryos is consistent with impaired active hepatoblast movement or the loss of spatial information, respectively. In line with this idea EphB3b expression in the LPM and gut delineates the right as well as the anterior and posterior limits of the liver bud. Ectopic expression of $EphB3b^{\Delta ICD}$ on the left side directs hepatoblasts toward the opposite side of the embryo, while gut tissue remains unaffected, supporting asymmetric repulsive EphB3b-EphrinB1 interaction controlling oriented hepatoblast movement. We therefore propose that EphB3b from LPM cells interacts with EphrinB1 at hepatoblast membranes having a local effect on the cytoskeleton resulting in protrusion collapse, lamellipodia orientation, hepatoblast polarization, and directional migration. These hepatoblast-LPM interactions are reminiscent of morphogenetic movements at the ectoderm-mesoderm border during *Xenopus* gastrulation, where Eph/Ephrin control protrusive activity and cycles of cell attachment and detachment indicating repulsive activity between the two tissues (Rohani et al., 2011). In addition to providing the spatial cues for migrating hepatoblasts via activation of reverse signaling, EphB3b forward signaling mediates the differentiation of a highly polarized LPM epithelium underlying the asymmetric migration of this tissue. Asymmetric LPM migration in turn ensures that the interaction interface and the signaling between the LPM and hepatoblasts is maintained during progressive leftward outgrowth. Unlike the existing model for asymmetric liver positioning, our results indicate that (1) hepatoblasts actively migrate into the liver bud, (2) the LPM directs the movement of hepatoblasts during budding by triggering cell repulsion, and (3) the endoderm signals back to the LPM epithelium controlling its epithelial organization and asymmetric migration. Furthermore, our study supports the notion that neuronal guidance factors have functions outside the developing nervous system (Adams and Eichmann, 2010), including endoderm morphogenesis (Domyan et al., 2013; Klein et al., 2011).

The unique characteristic of EphrinB/EphB bidirectional signaling in eliciting specific cellular responses allows the execution of specialized behaviors of each cell population. In the LPM, EphB3b controls the differentiation of the initially squamous epithelia into a highly polarized morphology, which is prerequisite for the distinct migration of the left and right LPM (Horne-Badovinac, 2003). This is reminiscent of EphB receptors promoting

mesenchymal to epithelial transition in several cancer cells (Chiu et al., 2009; Cortina et al., 2007) and in the developing zebrafish where EphB4a regulates epithelialization of somites (Barrios et al., 2003). In hepatoblasts, EphrinB1 controls cell-shape changes and the formation of cellular protrusions. EphrinB1 and EphB3b knockdowns exhibit similar mis-positioning of hepatoblasts, however they unexpectedly result in opposite defects in protrusion formation indicative of EphB3b-dependent and -independent functions of EphrinB1 during budding. EphB-independent functions of EphrinBs were reported in cultured cells where expression of mutant EphrinB variants unable to interact with EphB led to a dramatic increase of filopodia or cell-shape changes (Bochenek et al., 2010; Tomita et al., 2006). We show that EphrinB1 controls protrusion formation via its N-terminal PDZ-binding domain. Interaction between EphrinBs and several PDZ-domain-containing proteins has been reported to occur constitutively and to be antagonized by receptor-mediated activation of EphrinBs (Brückner et al., 1999). This is consistent with our observation that hepatoblasts have more protrusions upon EphB3b knockdown, suggesting that EphB3b-EphrinB1 binding may destabilize protrusions via tyrosine phosphorylation. The different effect on hepatoblast protrusions could also be explained by compensation through other Eph receptors interacting with EphrinB1, however the similarity of the phenotypes after knockdown of EphB3b or expression of the extracellular domain of EphrinB1 suggests that EphB3b is the main receptor of EphrinB1 in this context.

Cell-cell communication is essential for guiding moving cells and coordinating these movements with those of other tissues. In contrast to tissue interaction via secreted signals, communication by cell protrusions allows the signal to be delivered to the receiving cell with a high degree of control and at a distance. Protrusion formation and their functions are best understood in isolated cells in culture (Faix et al., 2009; Ridley, 2011), while this is less clear in the multicellular context of the developing embryo. Several recent studies identified cellular extensions in developing tissues with roles in long-distance signaling. Live imaging in *Drosophila* wing disc first uncovered cytonemes, very long actin-rich protrusions involved in gradient formation and tissue patterning that transport Hedgehog or the Bmp ligand Dpp to receiving cells at a distance (Bischoff et al., 2013; Roy et al., 2014). Short filopodia, have been implicated in establishing the bristle pattern of the *Drosophila* notum by Notch signaling (Cohen et al., 2010) and patterning the zebrafish neural plate by transport of Wnt8 (Stanganello et al., 2015). Our data support the involvement of protrusions in choreographing interlinked tissue movements in liver morphogenesis. Using sparse labeling, we uncovered that both hepatoblasts and LPM cells form protrusions, which ensure that morphogenetic movements are precisely controlled not only at the immediate interface between the two tissues but throughout the entire tissue. Moreover, in all of the above examples, distinctions are made between filopodia sending or receiving signals, whereas, in the liver bud, protrusions likely mediate EphrinB1/EphB3b bidirectional signaling. Two lines of evidence support that cellular protrusions are involved in communication between hepatoblasts and LPM at the direct tissue interface and over a distance: (1) several rows of hepatoblasts adjacent to an EphB3b$^{\Delta ICD}$ clone display no EphrinB1 at the membrane, which is likely due to endocytosis of the Ephrin/Eph complex following direct cell-cell contacts (Pitulescu and Adams, 2010) and (2) in transient *ephrinb1* CRISPR/Cas9 experiments, EphrinB1-positive hepatoblasts accumulate away from the EphrinB1-EphB3b tissue interface in the budding area separated from the LPM by 1–6 hepatoblasts expressing no or low level of EphrinB1 (Figure S4), strongly supporting long-distance intercellular communication. Although basal filopodia were reported to assist signaling within an epithelium in *Drosophila* (Callejo et al., 2011; Cohen et al., 2010), we propose a rare scenario with protrusions facilitating contact-dependent communication between different tissue types, the epithelial LPM and mesenchyme-like hepatoblasts in the context of liver budding (Figure S5). Coordinating the collective movement of tissues with different architecture by cell protrusions is an effective mechanism for communication over a distance, which is likely relevant in many other tissue contexts including invasive transformed cells.

EXPERIMENTAL PROCEDURES

All experiments were performed in agreement with the NIMR and KU ethical review committees.

Generation of Transgenic Lines

Standard cloning and transgenesis techniques were used to generate TgBAC (prox1a:GalTA4-4xUAS-E1b:uncTagRFP)^{nim5}, Tg(UAS-E1b:lyn-Citrine)^{nim23}, Tg(UAS-E1b:ephrinB1)^{nim24} Tg(UAS-E1b:ephrinB1^{EC})^{nim25}, and Tg(hsp70l:gal4)^{fci1}.

Generation of Genetic Mutants

ephrinb1^{nim26} mutants were generated by CRISPR (clustered regularly interspaced short palindromic repeats)/Cas9 injections into one-cell-stage zebrafish embryos. Oligonucleotides targeting the genomic sequence 5′-GGA CAT TAT CTG CCC CAA AG-3′ in the second exon of the zebrafish *ephrinb1* locus were cloned into the pDR274 plasmid for gRNA production (Hwang et al., 2013). Mutations were identified by amplicon restriction using the primers efnb1F 5′-GTT TGT GTC TGG GAA GGG CTT AG-3′ and efnb1R 5′-TAT GGT GCT GCA GGA CTC GGC CTG-3′, followed by XcmI restriction and verification by sequencing. A stable line *ephrinb1*^{nim26} was raised carrying a 4 bp deletion and a 3 bp insertion causing a frameshift and the occurrence of a premature stop codon at position 54. Immunostaining for EphrinB1 revealed a complete absence of protein in homozygous embryos, indicating a complete loss of function.

Immunostaining and mRNA Stainings

Rabbit α-Prox1 (Chemicon), mouse α-Prox1 (Abcam), and mouse α-ZO1 (Invitrogen) were used for immunostainings. Polyclonal antibodies against EphrinB1 and EphB3b were produced in rabbit and guinea pig, respectively.

Whole-mount in situ hybridization was performed with antisense mRNA probes for *ceruloplasmin, ephrinb1*, and *hhex*. An *ephb3b* riboprobe was generated from a 690 bp fragment, located 3′ of the Ephrin-binding domain.

Morpholino Knockdown

Antisense morpholino oligonucleotides (Gene Tools) blocking translation or splicing of *ephrinb1* (*MOatg-ephrinb1*, *MOdon-ephrinb1*) and *ephb3b* (*MOdon-ephb3b*, *MOacc-ephb3b*) were injected into one-cell-stage embryos. Morpholinos with 5 bp mismatches were injected as controls (*MOdon-mism-ephrinb1*, *MOacc-mism-ephb3b*), producing no consistent phenotypes.

Injection of DNA Constructs

ephrinb1, *ephrinb1*^{6F}, and *ephrinb1*$^{\Delta V}$ were placed behind the *hsp70l* promoter and between *minitol2* sequences. They were co-injected with *transposase* mRNA (30 pg mRNA and 20 pg DNA/embryo) in one-cell-stage embryos and subjected to heat shocks at 39°C: 45 min at 22 hpf and 30 min at 28 hpf. *lyn-tdTomato*, *lyn-citrine*, and *utrophin-gfp* were placed behind the *ubiquitin* (*ubi*) promoter and between *minitol2* sequences and injected as above.

Quantification of Hepatoblast Characteristics

Membrane-tethered fluorescent proteins were expressed in single cells or small clones by injection of DNA constructs, in which lyn-tdTomato and lyn-Citrine are under the control of the *ubiquitin* promoter, or by applying a binary transgenic approach for which the *TgBAC(prox1a:KalT4-4xUAS:uncTagRFP)*^{nim5} transgenic line (hereafter referred to as *Tg(prox1a:KalT4)*^{nim5} is crossed with *Tg(UAS:lynCitrine)*^{nim23}. Notably, the stable *Tg(UAS:lynCitrine)*^{nim23} line shows mosaic expression, likely due to partial silencing. Hepatoblast shape was determined by measuring their length and width in coronal or transverse sections of whole-liver confocal stacks to calculate the L/W ratio.

Hepatoblast protrusions were manually tracked in three dimensions through consecutive sections of confocal stacks from whole-mount livers (coronal views). Protrusions with a diameter ≤ 1.5 μm were classified as filopodia-like (simple and branched), while flat protrusions with larger diameter were classified as lamellipodia-like. Protrusion quantity was determined as the absolute number per square micrometer of cell surface.

The orientation angle of hepatoblasts or their protrusions was determined with respect to the anteroposterior axis of the embryo. All measurements were performed with Volocity software (Improvision).

SUPPLEMENTAL INFORMATION

Supplemental Information includes Supplemental Experimental Procedures, five figures, one table, and four movies and can be found with this article online at http://dx.doi.org/10.1016/j.devcel.2016.10.009.

AUTHOR CONTRIBUTIONS

J.C. and E.A.O. designed the study, analyzed data, and wrote the manuscript. J.C. performed experiments. A.D. and S.C. developed the live-imaging strategy. A.D. generated and analyzed time-lapse data; G.K. developed the routine for cell-displacement analysis. J.B. and G.J.W. generated reagents for antibody production. J.C.F. generated transgenic lines. J.C., A.D., S.C., and E.A.O. edited the manuscript.

ACKNOWLEDGMENTS

We thank C. Brazill-Adams and P. Lundegaard and their teams for expert fish care, K. Rittinger and P. Walker for their help with protein purification, S. Scholpp and R. Köster for constructs, S. Gerety and D. Wilkinson for constructs and the unpublished *Tg(hsp70l:gal4)*^{fci1} line, and I. Salecker, D. Stamataki, J.-P. Vincent, and Q. Xu for discussions or suggestions on the manuscript. We thank D. Wilkinson for his support throughout the project. This work was funded by the Medical Research Council (U117581329), Novo Nordisk Foundation (NNF15CC0018344), Danish National Research Foundation (DNRF116), Wellcome Trust (098051), and an Early Postdoc Mobility Fellowship from the Swiss National Science Foundation to S.C..

Received: April 28, 2015
Revised: July 24, 2016
Accepted: October 10, 2016
Published: November 7, 2016

REFERENCES

Adams, R.H., and Eichmann, A. (2010). Axon guidance molecules in vascular patterning. Cold Spring Harb. Perspect. Biol. *2*, a001875.

Barrios, A., Poole, R.J., Durbin, L., Brennan, C., Holder, N., and Wilson, S.W. (2003). Eph/Ephrin signaling regulates the mesenchymal-to-epithelial transition of the paraxial mesoderm during somite morphogenesis. Curr. Biol. *13*, 1571–1582.

Bischoff, M., Gradilla, A.-C., Seijo, I., Andrés, G., Rodríguez-Navas, C., González-Méndez, L., and Guerrero, I. (2013). Cytonemes are required for the establishment of a normal Hedgehog morphogen gradient in *Drosophila* epithelia. Nat. Cell Biol. *15*, 1269–1281.

Bochenek, M.L., Dickinson, S., Astin, J.W., Adams, R.H., and Nobes, C.D. (2010). Ephrin-B2 regulates endothelial cell morphology and motility independently of Eph-receptor binding. J. Cell Sci. *123*, 1235–1246.

Bort, R., Signore, M., Tremblay, K., Barbera, J.P.M., and Zaret, K.S. (2006). Hex homeobox gene controls the transition of the endoderm to a pseudostratified, cell emergent epithelium for liver bud development. Dev. Biol. *290*, 44–56.

Brückner, K., Pablo Labrador, J., Scheiffele, P., Herb, A., Seeburg, P.H., and Klein, R. (1999). EphrinB ligands recruit GRIP family PDZ adaptor proteins into raft membrane microdomains. Neuron *22*, 511–524.

Burkel, B.M., von Dassow, G., and Bement, W.M. (2007). Versatile fluorescent probes for actin filaments based on the actin-binding domain of utrophin. Cell Motil. Cytoskeleton *64*, 822–832.

Bush, J.O., and Soriano, P. (2010). Ephrin-B1 forward signaling regulates craniofacial morphogenesis by controlling cell proliferation across Eph-ephrin boundaries. Genes Dev. *24*, 2068–2080.

Callejo, A., Bilioni, A., Mollica, E., Gorfinkiel, N., Andrés, G., Ibáñez, C., Torroja, C., Doglio, L., Sierra, J., and Guerrero, I. (2011). Dispatched mediates Hedgehog basolateral release to form the long-range morphogenetic gradient in the *Drosophila* wing disk epithelium. Proc. Natl. Acad. Sci. USA *108*, 12591–12598.

Chiu, S.-T., Chang, K.-J., Ting, C.-H., Shen, H.-C., Li, H., and Hsieh, F.-J. (2009). Over-expression of EphB3 enhances cell-cell contacts and suppresses tumor growth in HT-29 human colon cancer cells. Carcinogenesis *30*, 1475–1486.

Chung, W.-S., Shin, C.H., and Stainier, D.Y.R. (2008). Bmp2 signaling regulates the hepatic versus pancreatic fate decision. Dev. Cell *15*, 738–748.

Cohen, M., Georgiou, M., Stevenson, N.L., Miodownik, M., and Baum, B. (2010). Dynamic filopodia transmit intermittent delta-notch signaling to drive pattern refinement during lateral inhibition. Dev. Cell *19*, 78–89.

Cortina, C., Palomo-Ponce, S., Iglesias, M., Fernández-Masip, J.L., Vivancos, A., Whissell, G., Humà, M., Peiró, N., Gallego, L., Jonkheer, S., et al. (2007). EphB-ephrin-B interactions suppress colorectal cancer progression by compartmentalizing tumor cells. Nat. Genet. *39*, 1376–1383.

Costa, R.M.B., Mason, J., Lee, M., Amaya, E., and Zorn, A.M. (2003). Novel gene expression domains reveal early patterning of the *Xenopus* endoderm. Gene Expr. Patterns *3*, 509–519.

Cowan, C.A., and Henkemeyer, M. (2002). Ephrins in reverse, park and drive. Trends Cell Biol. *12*, 339–346.

Davis, N.M., Kurpios, N.A., Sun, X., Gros, J., Martin, J.F., and Tabin, C.J. (2008). The chirality of gut rotation derives from left-right asymmetric changes in the architecture of the dorsal mesentery. Dev. Cell *15*, 134–145.

Davy, A., Aubin, J., and Soriano, P. (2004). Ephrin-B1 forward and reverse signaling are required during mouse development. Genes Dev. *18*, 572–583.

Domyan, E.T., Branchfield, K., Gibson, D.A., Naiche, L.A., Lewandoski, M., Tessier-Lavigne, M., Ma, L., and Sun, X. (2013). Roundabout receptors are critical for foregut separation from the body wall. Dev. Cell *24*, 52–63.

Faix, J., Breitsprecher, D., Stradal, T.E.B., and Rottner, K. (2009). Filopodia: complex models for simple rods. Int. J. Biochem. Cell Biol. *41*, 1656–1664.

Field, H.A., Ober, E.A., Roeser, T., and Stainier, D.Y.R. (2003). Formation of the digestive system in zebrafish. I. Liver morphogenesis. Dev. Biol. *253*, 279–290.

Fletcher, F.A., Carpenter, M.K., Shilling, H., Baum, P., Ziegler, S.F., Gimpel, S., Hollingsworth, T., Vanden Bos, T., James, L., and Hjerrild, K. (1994). LERK-2, a binding protein for the receptor-tyrosine kinase ELK, is evolutionarily conserved and expressed in a developmentally regulated pattern. Oncogene *9*, 3241–3247.

Hecksher-Sorensen, J. (2004). The splanchnic mesodermal plate directs spleen and pancreatic laterality, and is regulated by Bapx1/Nkx3.2. Development *131*, 4665–4675.

Hochgreb-Hagele, T., Yin, C., Koo, D.E.S., Bronner, M.E., and Stainier, D.Y.R. (2013). Laminin 1a controls distinct steps during the establishment of digestive organ laterality. Development *140*, 2734–2745.

Holder, N., and Klein, R. (1999). Eph receptors and ephrins: effectors of morphogenesis. Development *126*, 2033–2044.

Horne-Badovinac, S. (2003). A cellular framework for gut-looping morphogenesis in zebrafish. Science *302*, 662–665.

Hwang, W.Y., Fu, Y., Reyon, D., Maeder, M.L., Tsai, S.Q., Sander, J.D., Peterson, R.T., Yeh, J.-R.J., and Joung, J.K. (2013). Efficient genome editing in zebrafish using a CRISPR-Cas system. Nat. Biotechnol. *31*, 227–229.

Kania, A., and Klein, R. (2016). Mechanisms of ephrin–Eph signalling in development, physiology and disease. Nat. Rev. Mol. Cell Biol. *17*, 240–256.

Klein, C., Mikutta, J., Krueger, J., Scholz, K., Brinkmann, J., Liu, D., Veerkamp, J., Siegel, D., Abdelilah-Seyfried, S., and le Noble, F. (2011). Neuron navigator 3a regulates liver organogenesis during zebrafish embryogenesis. Development *138*, 1935–1945.

Lüdtke, T.H.-W., Christoffels, V.M., Petry, M., and Kispert, A. (2009). Tbx3 promotes liver bud expansion during mouse development by suppression of cholangiocyte differentiation. Hepatology *49*, 969–978.

Margagliotti, S., Clotman, F., Pierreux, C.E., Beaudry, J.-B., Jacquemin, P., Rousseau, G.G., and Lemaigre, F.P. (2007). The Onecut transcription factors HNF-6/OC-1 and OC-2 regulate early liver expansion by controlling hepatoblast migration. Dev. Biol. *311*, 579–589.

Nalbant, P., Hodgson, L., Kraynov, V., Toutchkine, A., and Hahn, K.M. (2004). Activation of endogenous Cdc42 visualized in living cells. Science *305*, 1615–1619.

Nobes, C.D., and Hall, A. (1995). Rho, rac, and cdc42 GTPases regulate the assembly of multimolecular focal complexes associated with actin stress fibers, lamellipodia, and filopodia. Cell *81*, 53–62.

Ober, E.A., Verkade, H., Field, H.A., and Stainier, D.Y.R. (2006). Mesodermal Wnt2b signalling positively regulates liver specification. Nature *442*, 688–691.

Pasquale, E.B. (2005). Developmental Cell Biology: Eph receptor signalling casts a wide net on cell behaviour. Nat. Rev. Mol. Cell Biol. *6*, 462–475.

Pitulescu, M.E., and Adams, R.H. (2010). Eph/ephrin molecules–a hub for signaling and endocytosis. Genes Dev. *24*, 2480–2492.

Poliakov, A., Cotrina, M., and Wilkinson, D.G. (2004). Diverse roles of Eph receptors and ephrins in the regulation of cell migration and tissue assembly. Dev. Cell *7*, 465–480.

Poulain, M., and Ober, E.A. (2011). Interplay between Wnt2 and Wnt2bb controls multiple steps of early foregut-derived organ development. Development *138*, 3557–3568.

Ridley, A.J. (2011). Life at the leading edge. Cell *145*, 1012–1022.

Rohani, N., Canty, L., Luu, O., Fagotto, F., and Winklbauer, R. (2011). EphrinB/EphB signaling controls embryonic germ layer separation by contact-induced cell detachment. PLoS Biol. *9*, e1000597, 18.

Roy, S., Huang, H., Liu, S., and Kornberg, T.B. (2014). Cytoneme-mediated contact-dependent transport of the *Drosophila* decapentaplegic signaling protein. Science *343*, 1244624.

Shin, D., Weidinger, G., Moon, R.T., and Stainier, D.Y.R. (2012). Intrinsic and extrinsic modifiers of the regulative capacity of the developing liver. Mech. Dev. *128*, 525–535.

Sosa-Pineda, B., Wigle, J.T., and Oliver, G. (2000). Hepatocyte migration during liver development requires Prox1. Nat. Genet. *25*, 254–255.

Stanganello, E., Hagemann, A.I.H., Mattes, B., Sinner, C., Meyen, D., Weber, S., Schug, A., Raz, E., and Scholpp, S. (2015). Filopodia-based Wnt transport during vertebrate tissue patterning. Nat. Commun. *6*, 5846.

Thisse, C., and Thisse, B. (2005). High Throughput Expression Analysis of ZF-Models Consortium Clones, (accessed September 29, 2016). http://zfin.org.

Tomita, T., Tanaka, S., Morohashi, Y., and Iwatsubo, T. (2006). Presenilin-dependent intramembrane cleavage of ephrin-B1. Mol. Neurodegener. *1*, 2.

Wallace, K.N., Yusuff, S., Sonntag, J.M., Chin, A.J., and Pack, M. (2001). Zebrafishhhex regulates liver development and digestive organ chirality. Genesis *30*, 141–143.

Yaron, A., and Sprinzak, D. (2012). The cis side of juxtacrine signaling: a new role in the development of the nervous system. Trends Neurosci. *35*, 230–239.

Yin, C., Kikuchi, K., Hochgreb, T., Poss, K.D., and Stainier, D.Y.R. (2010). Hand2 regulates extracellular matrix remodeling essential for gut-looping morphogenesis in zebrafish. Dev. Cell *18*, 973–984.

Zimmer, M., Palmer, A., Köhler, J., and Klein, R. (2003). EphB-ephrinB bi-directional endocytosis terminates adhesion allowing contact mediated repulsion. Nat. Cell Biol. *5*, 869–878.

Zorn, A.M., and Wells, J.M. (2007). Molecular basis of vertebrate endoderm development. Int. Rev. Cytol. *259*, 49–111.

Developmental Cell, Volume *39*

Supplemental Information

EphrinB1/EphB3b Coordinate Bidirectional Epithelial-Mesenchymal Interactions Controlling Liver Morphogenesis and Laterality

Jordi Cayuso, Aliaksandr Dzementsei, Johanna C. Fischer, Gopal Karemore, Sara Caviglia, Josefin Bartholdson, Gavin J. Wright, and Elke A. Ober

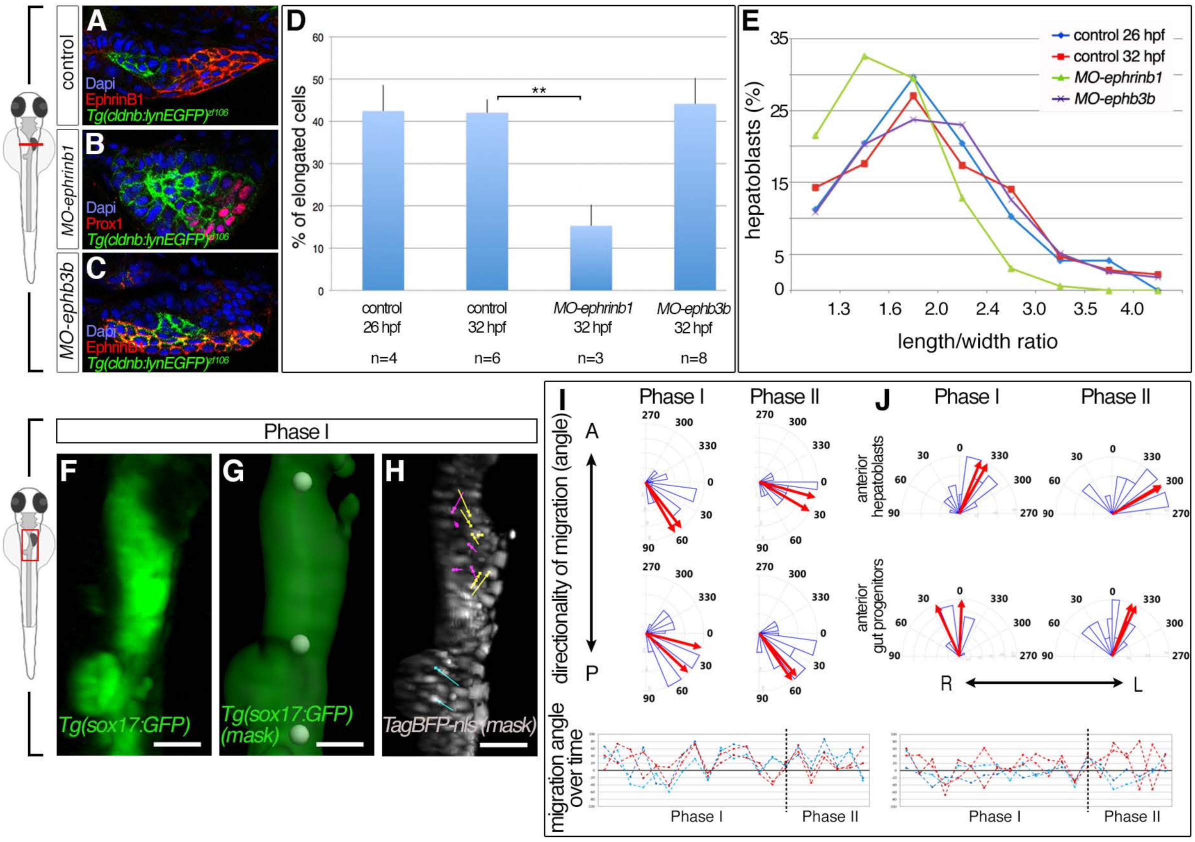

Cayuso et al. Figure S1

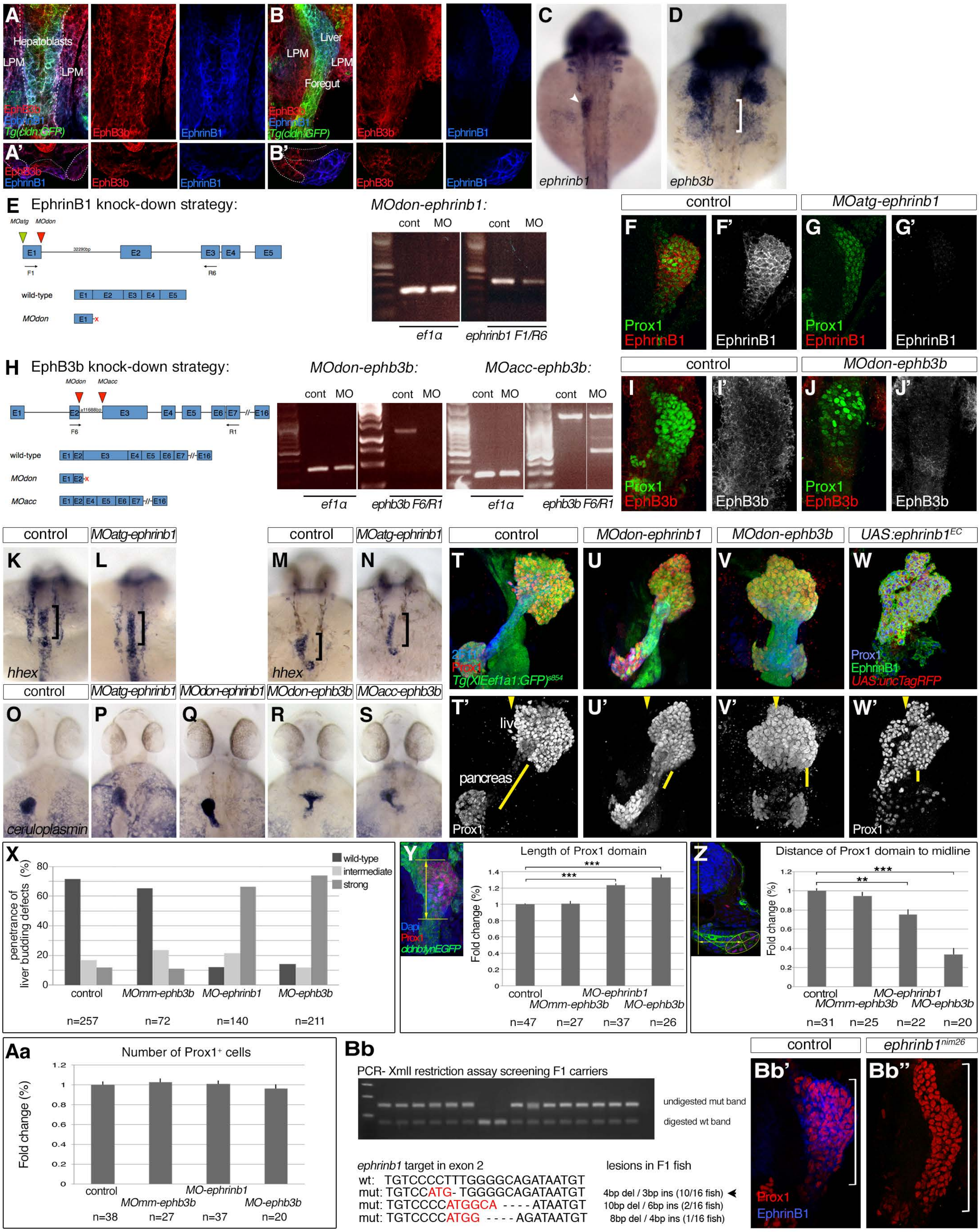

Cayuso et al. Figure S2

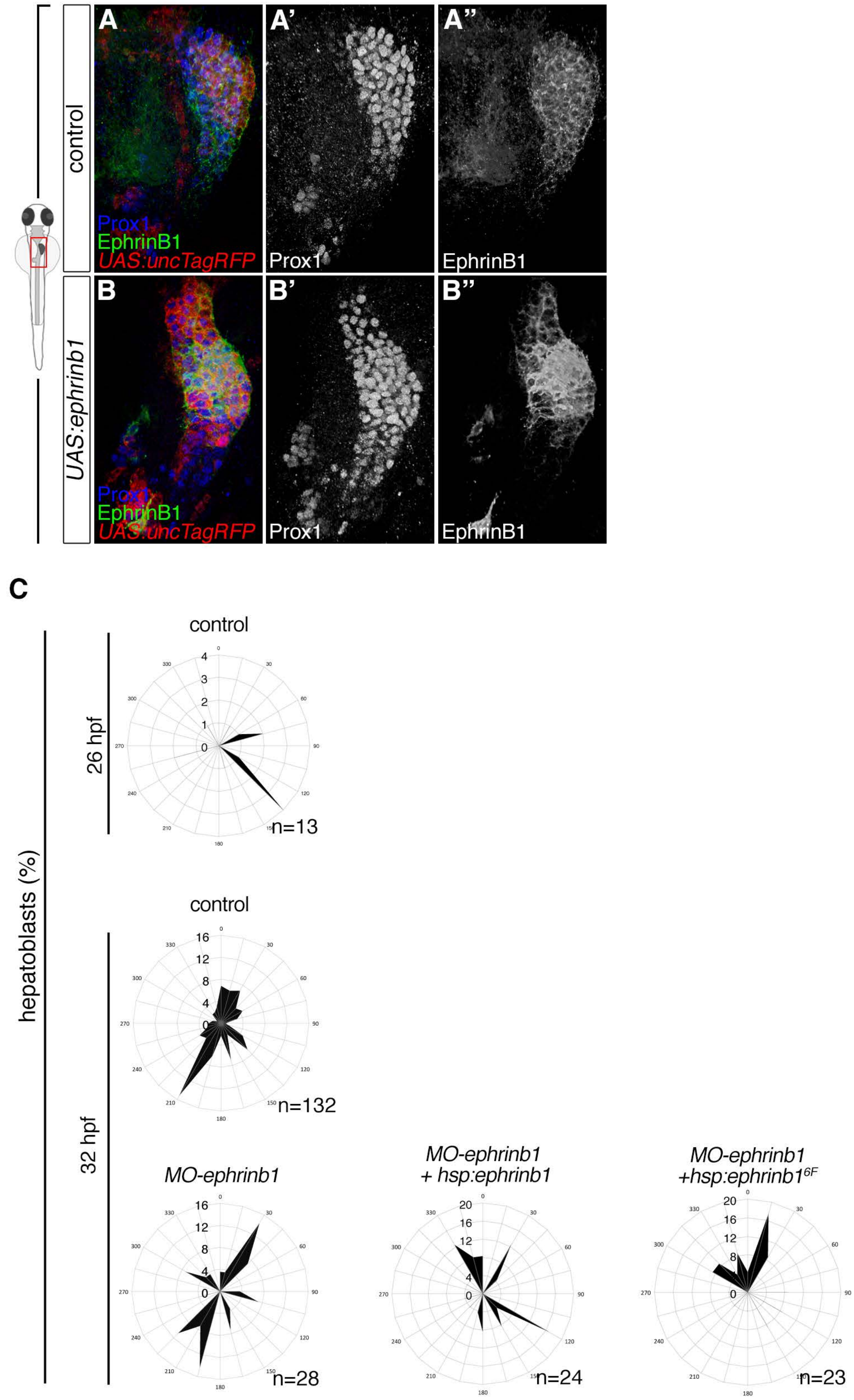

Cayuso et al. Figure S3

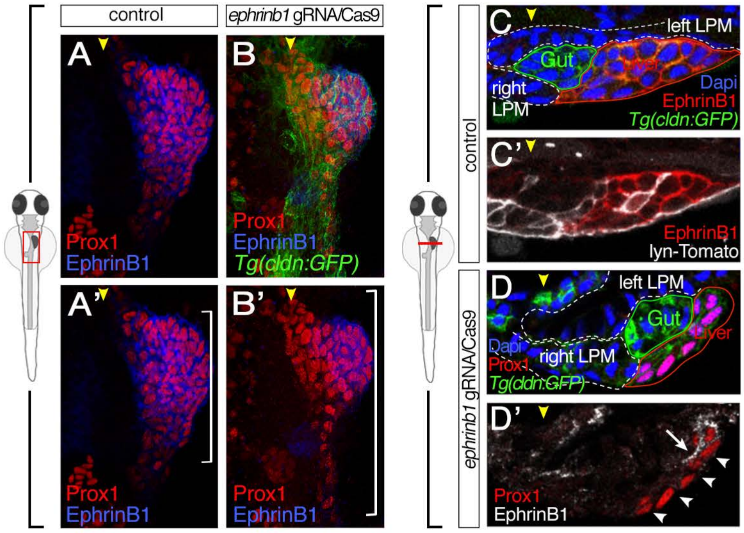

Cayuso et al. Figure S4

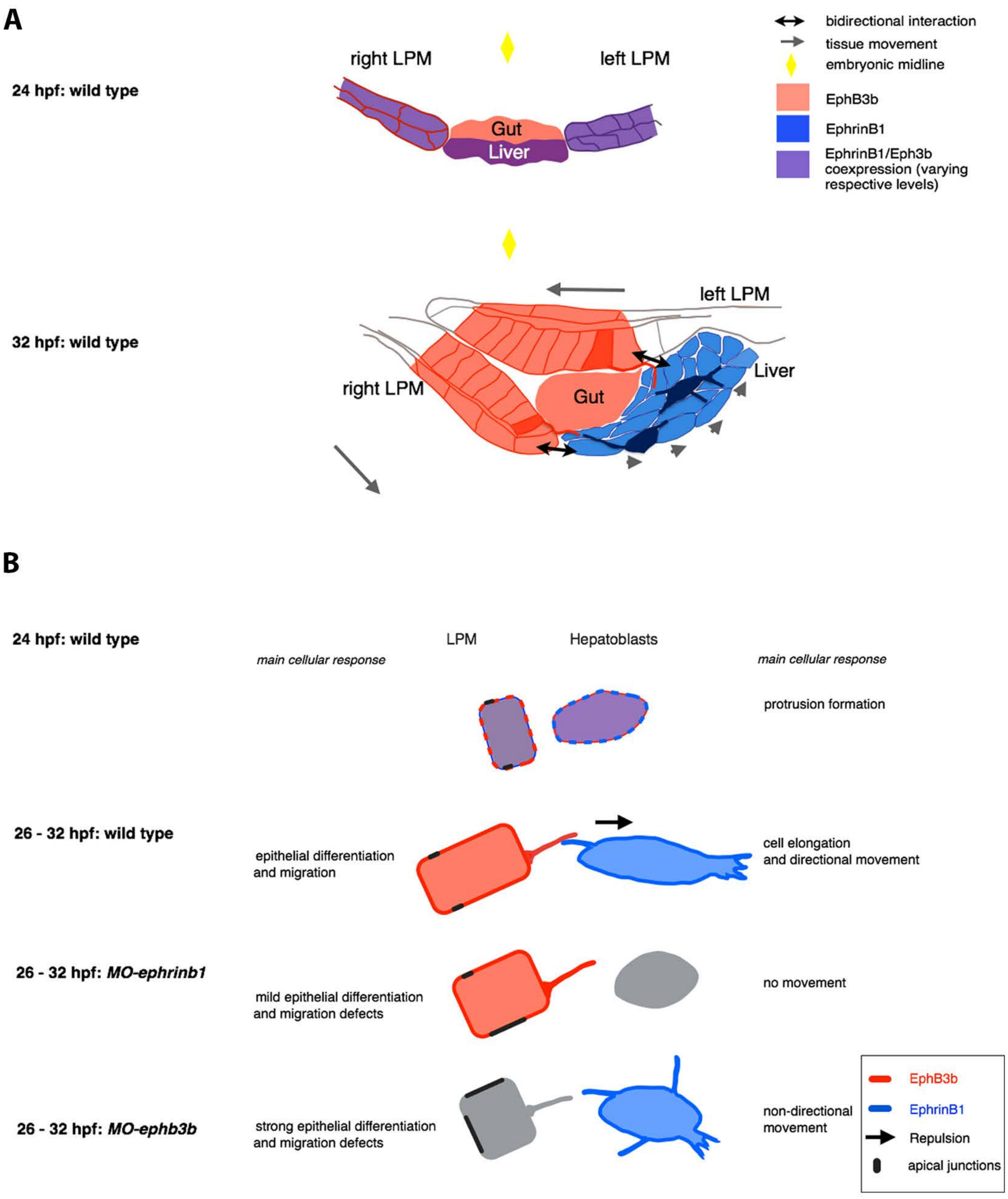

Cayuso et al. Figure S5

Supplemental legends

Supplemental Table S1: Table displaying changes in hepatoblast protrusion formation by comparing different control stages and/or diverse experimental conditions at 32 hpf. Related to Figure 6. p-values were determined by student's t-test (*, p<0.05; **, p <0.01; ***, p <0.001).

Supplemental Figure S1: Hepatoblasts undergo dynamic cell shape changes during liver budding. Related to Figure 1.
(A-C) Cell morphology was determined with EphrinB1- or *Tg(-0.8cldnb:lynEGFP)*^{zf106}-expression outlining the hepatoblasts in control (A), *MO-ephrinB1* (B) and *MO-ephB3b* embryos (C); transverse sections with liver oriented to the right. Morphometric measurements were carried out on serial sections through the bud. **(D,E)** Quantification of hepatoblast characteristics during liver budding in control, *MO-ephrinB1* and *MO-ephB3b* embryos: (D) population of elongated cells (length/width≥2) per bud and (E) hepatoblast distribution according to length/width ratio; only *MO-ephrinB1* exhibit a significantly decreased length/width ratio). Error bars represent standard error of the mean; ** = p<0.01. **(F-H)** Processing steps of 4D dataset of a *Tg(sox17:GFP)*^{s99} embryo injected with *tagBFP-nls* mRNA capturing liver budding (see Figure 1J-N, movie S1). *sox17:GFP* expression (F) marks the endoderm and was used to generate a foregut mask (G) which was subsequently applied to the TagBFP-nls channel (H). Three manually selected anatomical landmarks in 3D were used for drift correction (G, round spheres). Scale bar - 40µm. **(I,J)** Rose plots represent distribution of angular displacement (blue sectors) for hepatoblasts and gut progenitors from the same anterior position and angle of mean displacement per cell for the entire period (red arrow). Displacements were recorded every 28 min with respect to the embryonic midline and left-right distribution. Line plots representing directionality of displacement over time reveal differences in individual angular cell displacement for various hepatoblasts (red hues) and gut progenitors (blue hues). Data are derived from movie S1 (see also Figure 1 J-N).

Supplemental Figure S2: EphrinB1 and EphB3b expression and validation of knock down and knock out strategies. Related to Figure 4.
(A-B') Single channel views of immunostainings for EphrinB1 and EphB3b at 22 and 32 hpf shown in Figure 4A-B'''. **(C,D)** *ephrinb1and ephb3b* are expressed in the foregut area. At 30 hpf, *ephrinb1* is expressed in the liver (arrowhead) and *ephb3b* in hepatic area (bracket). **(E)** Schematic representation of *ephrinB1* knock-down strategy, with relevant MOs indicated. RT-PCR analysis of *MOdon-ephrinB1* embryos at 28 hpf shows a strong knock-down. In addition, a stop codon in the intronic sequence terminates the resulting protein 3 bps after the exon1-intron boundary. Both *MO-ephrinB1* produce similar phenotypes (see O-Q), *MOatg-ephrinB1* were used for all experiments. **(F-G')** EphrinB1 expression is abolished or barely detectable in *MOatg-ephrinB1* embryos at 32 hpf. **(H)** Schematic representation of *ephB3b* knock-down strategy, with relevant MOs indicated. *ephB3b* maps to linkage group 22 and is partly encoded by EST CT 623350. By 5'RACE, we identified a 2970 bp coding transcript encoded by 16 exons containing all motifs conserved between EphB-receptors, including a N-terminal signal peptide and Ephrin-binding domain. In addition, we found an alternative transcript missing the first two exons, replaced by a N-terminal extension of exon 3 and lacking a signal peptide and a clear transcriptional start site. Its functional significance is unclear and it was not targeted by our knock-down strategy. RT-PCR analysis of *MOdon-ephB3b* embryos at 28 hpf shows a complete knock-down, indicated by a complete loss of the Ephrin-binding domain-containing exon3. In addition, a stop codon in the intronic sequence would terminate the resulting protein 108 bps after the exon 2-intron boundary. RT-PCR analysis of *MOacc-ephB3b* embryos at 28 hpf reveals a partial knock-down. This is consistent with the loss of the Ephrin-binding domain-containing exon 3 in a fraction of the transcripts, as indicated by the appearance of a 500bp smaller ectopic band in the *MOacc-ephB3b* lane compared to controls; control and representative *MOacc-ephB3b* sample are from different parts of the same gel, as indicated by the separating line. Although, both *MO-ephB3b* produce similar phenotypes (see O,R,S), the *MOdon-ephB3b* knock-down is consistently more complete and was used for all experiments. **(I-J')** EphB3b expression is abolished or barely detectable in *MOdon-ephB3b* embryos at 32 hpf. **(K-N)** *hhex* mRNA expression at 25 hpf reveals that the liver anlage (bracket) forms in *MO-ephrinB1* embryos (L) similar to controls (G), while impaired hepatoblast (bracket) movement into the liver bud in *MO-ephrinB1* embryos is apparent at 28 hpf (M,N). **(O-S)** Two independent MOs targeting *ephrinB1* or *ephB3b* produce equivalent phenotypes, with hepatoblasts positioned more posterior and medial (P,Q and R,S, respectively) compared to controls at 48 hpf (O). Asymmetric hepatoblast positioning is generally more severely disrupted in *MO-ephB3b* than *MO-ephrinB1* embryos. **(T-W')** Confocal projections reveal liver morphogenesis defects in *MO-ephrinB1* (U,U'), MO-*ephB3b* (V,V'),

as well as embryos expressing EphrinB1^{EC} in the context of the entire foregut domain (W,W'). *Tg(XlEef1a1:GFP)^{s854}* marks the foregut endoderm, 2F11 the hepatopancreatic ducts and Prox1 the developing liver and pancreas at 54 hpf. Upon loss of EphrinB1 or EphB3b functions hepatoblasts are ectopically positioned in the hepatopancreatic duct domain compared to controls, as indicated by shorter yellow bars, and across the midline (arrowheads; T'-V'). Similar defects are observed upon conditional expression of EphrinB1^{EC} (W,W'). (C,D, K-S) dorsal views, (A-B, F-G', I-J',T-W') confocal projections of ventral views; all anterior to the top. (A',B') confocal images of transverse sections, left is oriented to the right.
(X-Aa) Quantification and morphometric analyses of liver bud development in *MO-ephrinB1* and *MO-ephB3b* embryos at 32 hpf. **(X)** Graph showing the proportion of embryos exhibiting hepatoblast positioning defects in un-injected and *MOmm-ephB3b* controls, and following EphrinB1- or EphB3b knockdown. In whole-mount preparations the length (Y) and width of the Prox1-domain and its proximity to the embryonic midline (Z) were used to classify the liver budding defects into three categories. **(Y)** Morphometric measurements showed a significant increase in Prox1-domain length (yellow double arrow) in *MO-ephrinB1* and *MO-ephB3b* embryos compared to controls. **(Z)** Morphometric measurements revealed a significant reduction in asymmetric displacement of the Prox1-domain from the midline in *MO-ephrinB1* and *MO-ephB3b* embryos compared to controls. Specifically, the distance of the centre of the Prox1-domain to the embryonic midline was determined in transverse sections (yellow double arrow). **(Aa)** EphrinB1 or EphB3b knockdown does not significantly change the number of Prox1-expressing hepatoblasts; p-values, for all conditions >0.57. (c,e,f) Standard errors are shown; asterisks indicate the p-value for each condition (**, $p<0.01$; ***, $p<0.001$). **(Bb-Bb")** Stable genetic *ephrinb1* mutant was generated using Crispr/Cas9 genome editing. F1 fish were screened for germline transmission of lesions by XmlI digest of amplified target DNA; undigested PCR bands indicate transmission of genetic lesion. Sequencing revealed that F1 fish carry different indels. Intercrosses of carriers with a 4bp deletion and 3bp insertion show a complete loss of α-EphrinB1 staining in homozygous carriers (Bb") compared to sibling controls (Bb'). A stable line, *ephrinb1^{nim26}*, was generated from these carriers.

Supplemental Figure S3: Altering EphrinB1 and EphB3b levels leads to defects in lamellipodia orientation and hepatoblast positioning. Related to Figure 6.
(A-B") Conditional expression of *UAS:ephrinB1* causes a posterior accumulation of hepatoblasts and morphological defects of the liver domain (B-B"). **(C)** Hepatoblast lamellipodia are oriented with respect to the anteroposterior axis. Expression of EphrinB1 and EphrinB1^{6F} randomises lamellipodia orientation in comparison to control and *MO-ephrinB1* embryos, and similar to compromised EphB3b function (see Figure 6H).

Supplemental Figure S4: Mosaic inactivation of EphrinB1 by Crispr/Cas9 editing, leads to defects in hepatoblast positioning and indicate long-distance contacts. Related to Figure 7.
(A-B') at 32 hpf, EphrinB1^{+}-hepatoblasts transiently expressing lyn-Tomato distribute randomly throughout the bud (A,A'), while EphrinB1^{+} hepatoblasts (blue) aggregate mostly anterior and away from the right LPM and hepatoblasts lacking EphrinB1 form an elongated Prox1 domain (B,B') closer to the midline (yellow arrowhead). White brackets indicate the length of the Prox1-domain. Confocal projections of ventral views; anterior to the top. **(C-D')** EphrinB1^{+} hepatoblasts (white) aggregate mostly away from the right LPM, often with a gap of 1-6 hepatoblasts lacking EphrinB1 (white arrowheads) indicating long-distance cell-cell contacts sensing repulsive EphB3b from the LPM. Consistently, hepatoblast protrusions (arrow) are also EphrinB1^{+} (D'). Transverse sections with dorsal to the top, and left to the right.

Supplemental Figure S5: Working model of how bidirectional EphrinB1/EphB3b activity coordinates hepatoblasts and LPM movements in liver budding. Related to Figure 7.
(A) EphrinB1 and EphB3b are dynamically expressed during liver budding. At 24 hpf, the start of budding both proteins are co-expressed in the LPM epithelia and newly specified hepatoblasts. Notably, EphB3b is higher and at the membrane in the right than left LPM, and the foregut solely expressed EphB3b. Co-expression is associated with cis-inhibition of signalling. By 32 hpf, EphrinB1 and EphB3b domains are complementary with EphrinB1 solely in hepatoblasts, and EphB3b in the LPM epithelia and gut. Single LPM cells (dark blue) and hepatoblasts (bright red) are highlighted to visualise cell protrusions and complex morphologies representative for all cells in the respective tissue. They reveal long-distance contacts between hepatoblasts and LPM cells allowing bidirectional EphrinB1 and EphB3b signalling (double headed arrows) essential for coordinated hepatoblast and

LPM movements (grey arrows). **(B)** Schematic representation of main cellular behaviours elicited by EphrinB1 and EphB3b in hepatoblasts and LPM epithelial cells, respectively.

Movie S1: Time lapse showing two phases of directional hepatoblast migration during liver bud formation. Related to Figure 1.
Tracking of liver (yellow), gut (magenta) and pancreas (white) progenitors in the *Tg(sox17:GFP)*-positive (green) foregut region; dorsal view. Cell nuclei were labelled by injection of *TagBFP-nls* mRNA (grey). Images were taken every 7 min and the monitored period is equivalent to 24 -34 hpf of development. Stills are shown in Figure 1. Scale bar - 40μm.

Movie S2: Sparse labelling of hepatoblasts reveals two phases of directional migration during liver bud formation. Related to Figure 1.
Tracking of liver (yellow), gut (magenta) and pancreas (white) progenitors in *Tg(sox17:GFP)*-positive (green) foregut region; dorsal view. Cell nuclei were labelled by DNA injection of *UAS:TagBFP-nls* (cyan) in embryos with transgenic expression of *prox1a:kalTA4-4xUAS:tagRFP* (red) coming up predominantly in hepatocytes. Images were taken every 28 min and the monitored period is equivalent to 24 -34 hpf of development; scale bar - 40μm.

Movie S3: Migrating hepatoblasts form filopodia- and lamellipodia-like protrusions during budding and the onset of outgrowth. Related to Figure 2.
Snapshots are included in Figure 2E. Transgenic *UAS:lyn-Citrine* (green) labels cell membranes. Images were taken every 23,5 min and the monitored period is equivalent to 24 -34 hpf of development; scale bar - 40μm.

Movie S4: Foregut organ formation proceeds normally during *live*-imaging. Related to Figure 1.
Dorsal view of the embryo with transgenic expression of *sox17:GFP* (green) marking the digestive system, *prox1a:kalTA4-4xUAS:tagRFP* (red) highlighting the liver and DNA injection of *UAS:TagBFP-nls* (cyan) marking nuclei. Images were taken every 21,5 min and the monitored period is equivalent to 24 -56 hpf of development; scale bar - 100μm.

	**lamellipodia/μm²**	**filopodia (branched)/μm²**	**filopodia/μm²**
**control 26hpf vs. control 32hpf**	** 0.0041	*** <0.0001	0.5277
**control 32 hpf vs. *MO-ephb3b***	0.5488	*** <0.0001	0.2059
**control 32 hpf vs. *MO-ephrinb1***	*** <0.0001	* 0.0105	0.6451
**control 32 hpf vs. *UAS:ephrinb1***	0.4496	*** <0.0001	** 0.009
MO-ephrinb1* vs. *MO-ephrinb1+hsp:ephrinb1	*** <0.0001	*** <0.0001	*** <0.0001
MO-ephrinb1* vs. *MO-ephrinb1+hsp:ephrinb1$^{\Delta V}$	0.3918	*** <0.0001	0.5283
MO-ephrinb1* vs. *MO-ephrinb1+hsp:ephrinb1^{6F}	* 0.0177	*** <0.0001	*** 0.0003
MO-ephrinb1+hsp:ephrinb1 vs. MO-ephrinb1+hsp:ephrinb1$^{\Delta V}$	*** <0.0001	0.8794	** 0.0092
MO-ephrinb1+hsp:ephrinb1 vs. MO-ephrinb1+hsp:ephrinb1^{6F}	0.105	0.3358	0.827

Cayuso et al. Table S1

Experimental Procedures

Fish stocks

Adult zebrafish and embryos were raised according to standard laboratory conditions (Westerfield, 2000). The following published strains were used: *Tg(XlEef1a1:GFP)*^{s854} (Field et al., 2003), *Tg(-0.8cldnb:lynEGFP)*^{zf106} (Haas and Gilmour, 2006), *Tg(-0.5sox17:GFP)*^{zf99} (Mizoguchi et al., 2008), *casanova/sox32*^{ta56} (Alexander et al., 1999) and wild-type. All experiments were performed in agreement with the NIMR and KU Ethical review committees.

Generation of transgenic lines

For the *prox1a BAC:kalTA4-4xUAS:uncTagRFP* transgenic driver line, a GalK cassette was recombined into the BAC clone DKEY-5J3 using PCR primers tagged with 75bp homology arms, and subsequently replaced with the final cassette (Distel et al., 2009; Warming et al., 2005) flanked by 470bp homology arms. Stable transgenic carriers were generated by injecting 25-100 pg BAC DNA into one-cell stage embryos, and screening adult fish for red fluorescent TagRFP expression in the liver. *TgBAC(prox1a:kalTA4-4xUAS:uncTagRFP)*^{nim5} are currently maintained in the F5 generation and was first described in (Dunworth et al., 2014).
Responder vectors were generated by cloning *lyn-Citrine,* full-length *ephrinb1* and *ephrinb1*^{EC}, encoding the extracellular domain and encompassing amino acids 1-236, under the control of five UAS sites and the E1b basal promoter (Köster and Fraser, 2001). To facilitate the identification of transgenic carriers, these constructs contain in addition *citrine* under the control of the lens-specific *α-Crystallin* promoter (Kurita et al., 2003). These constructs are flanked by miniTol2 elements (Urasaki et al., 2006). Stable transgenic carriers were generated by injecting 25 pg vector DNA and 40 pg *transposase* mRNA into one-cell stage embryos and raising embryos with Citrine-positive lenses to adulthood. Stable transgenic lines were established for *Tg(UAS:lyn-Citrine)*^{nim23}, *Tg(UAS:ephrinb1)*^{nim24} and *Tg(UAS:ephrinb1*^{EC}*)*^{nim25}, and are currently maintained in the F4 generation. UAS-mediated transgene expression is predominantly mosaic, as identified by lyn-Citrine expression or α-EphrinB1 stainings. In latter case, positive clones were distinguishable due to significantly higher transgenic EphrinB1 or EphrinB1^{EC} levels, which generally require for the detection by confocal microscopy only 50% of the photomultiplier gain used for endogenous EphrinB1 in the liver.

Immunohistochemistry

Labellings were performed as described (Ober et al., 2006). The following antibodies were used: rabbit α-Prox1 (1:1000; Chemicon), mouse α-Prox1 (1:100; Abcam), mouse α-2F11 (Abcam, 1:1000) and mouse α-ZO1 (1:200, Invitrogen). Cy3- and Cy5-conjugated secondary antibodies were obtained from Jackson Immunoresearch. Embryos were mounted in 4% agarose, sectioned at 70-130 μm using a Leica Vibratome and visualised using a Zeiss LSM5 Pascal Exciter confocal microscope. Images were processed with Volocity image analysis software (PerkinElmer) and Photoshop CS (Adobe).
Polyclonal antibodies against EphrinB1 and EphB3b were generated in rabbits and guinea pigs, respectively. The entire ectodomain region including the native signal peptide of zebrafish EphrinB1 and EphB3b were subcloned into a mammalian expression vector containing a C-terminal rat Cd4d3+4 tag followed by a hexa-his tag and expressed as a soluble recombinant protein using transient transfections of HEK293E cells, essentially as described (Bushell et al., 2008). Proteins were purified by Ni^{2+}-affinity chromatography (GE Healthcare). Protein concentration was determined by UV-spectroscopy using a computationally calculated extinction coefficient.

In Situ Hybridisation

Whole-mount mRNA in situ hybridisation was performed as described (Ober et al., 2006). Embryos older than 24 hpf were treated with 0.2mM 1-phenyl-2-thiourea in egg water to inhibit Melanin production. The following probes were used: *ceruloplasmin (Korzh et al., 2001),* and *hhex (Ho et al., 1999).* A riboprobe specific for *ephb3b* was generated cloning a 690 bp fragment, located 3' of the Ephrin-binding domain, into PCRII (Invitrogen). The following primers were used to amplify this sequence: Fw 5'- TCCGGGCTTTCTACAAGAAGT-3' and Rv 5'-AGGGACTCTTGCTGGCTACTC-3'.

Morpholino knockdown

Antisense morpholino oligonucleotides blocking translation or splicing of *ephrinb1* and *ephb3b* (Gene Tools, LLC) were injected into one-cell stage embryos. Morpholinos with 5 bp mismatches (*mm*) were injected as controls, producing no consistent phenotypes. 4 ng *MOatg-ephrinb1*, 2.2 ng *MOdon-*

ephrinb1, 1.5 ng *MOdon-ephb3b,* 1 ng *MOacc-ephb3b* and 1 ng *MOacc-mm-ephb3b* were injected per embryo. Sequences of morpholinos used in this study:
MOatg-ephrinb1 5'-TTGCCGAACCACATGCAACAGAGCG-3'
MOdon-ephrinb1 5'-TGCACTTACTTGGGATTTTGCGAAT-3'
MOdon-ephb3b 5'-TGTAAAAAGACAGCTCACCCCGGTC-3'
MOacc-ephb3b 5'-CCCACTGCATTAAAAAAAGAGAGCA-3'
MOacc-mm-ephb3b 5'-CgCACTGgATTAAAcAAAGAcAGgA-3'
The efficiency of the knockdown was tested by antibody staining against the targeted gene or by RT-PCR after RNA extraction with TRIzol reagent (Invitrogen) from 5 embryos per condition (Supplementary Fig. 1). Sequences of indicated PCR-primers: *ephrinb1*-F1: 5'-TTACCTCTGGATTCTGACAGC-3', *ephrinb1*-R6: 5'-GTTTTCCAAGCCTTCCTGTGT-3', *ephb3b*-F6: 5'-AAACACTGATGGACACCAAATG-3', *ephb3b*-R1: 5'-AGGGACTCTTGCTGGCTACTC-3'. Moreover, MO-injection into a p53-depleted background (*MO-p53* 5'-GCGCCATTGCTTTGCAAGAATTG-3') did not alter the observed liver morphogenesis phenotypes. These results together with the lack of apparent pyknotic nuclei, visualised by Dapi, indicate that MO-mediated EphrinB1 or EphB3b knock-down does not produce apparent unspecific defects, including apoptosis. Altogether, these findings confirm the specificity of the knock-down phenotypes.

Injection of DNA-constructs

<u>Constructs for ubiquitous expression:</u>The membrane-targeted *lyn-tdTomato* and *utrophin-gfp* (Burkel et al., 2007) were placed under the control of the *ubiquitin (ubi)* promoter (Mosimann et al.) and between *minitol2* sequences (Urasaki et al., 2006) and sequence verified.
<u>Heat-shock inducible (hsp70l) constructs:</u> Full-length and mutant forms of *ephrinb1* were placed under the control of the *hsp70l* promoter and between *minitol2* sequences and sequence verified. In *ephrinb1*^{6F}, six conserved tyrosines were replaced by phenylalanine (gene synthesis by Entelechon). Injected embryos were subjected to two heat-shocks at 39ºC: 45 min at 22 hpf and 30 min at 28 hpf. *ephb3bΔICD* lacking the intracellular domain was PCR-amplified using: *ephb3bΔICD-fw* 5'-CCGTCGACATGACAATGGATTATTTG-3'; *ephb3bΔICD*-rev 5'-ATCGAGATCTCGTCGATCCCT-3' primers. A *myc* tag was added to the N-terminus of the protein. Injected embryos were subjected to two heat-shocks at 39ºC: 30 min at 22 and 26 or 28 hpf. Embryos were fixed two or four hours later and immunostained. Positive clones were identified by Myc expression and/or increased EphB3b staining.
Kal4-inducible 5xUAS constructs: tagBFP-nls cassette was amplified from pBluescript-*tagBFP-nls* (Kanca et al., 2014); kind gift from Emmanuel Caussinus) using:
tagBFP-fw 5'-ATATGAATTCGCGTAGGGGATCCCAAAATG-3' and M13-rev 5'-TCACACAGGAAACAGCTATGAC-3' primers. Wild type *cdc42* and dominant negative *cdc42*^{T17N}-*EGFP*-fusion cassettes (kind gift from Steffen Scholpp) were extracted by PCR. All cassettes were cloned downstream of 5xUAS and the E1b basal promoter (Köster and Fraser, 2001). The constructs contain also *citrine* under the control of the lens-specific *α-Crystallin* promoter (Kurita et al., 2003), and it is flanked by *miniTol2* elements. Constructs were injected into *TgBAC(prox1a:kalTA4-4xUAS:uncTagRFP)*^{nim5} or *Tg(hsp70l:Gal4)*^{fci1} (kind gift from David Wilkinson).
All constructs (20-30 pg DNA/embryo) were co-injected with *transposase* mRNA (30-80 pg/embryo).

Latrunculin B treatment

Actin polymerisation was inhibited by Latrunculin B (LatB) (Morton, Ayscough, & McLaughlin, 2000). Given the importance of actin polymerisation for numerous cellular processes, we minimised the exposure to LatB and treated the embryos from 26 -32 hpf with low doses of the drug (0.1µg/ml in egg water). Consistent with a recent study incubating embryos >24 hpf with 0.15 µg/ml Lat B for an extended period of time (≥ 17 hours) (Phng, Stanchi, & Gerhardt, 2013), our short exposure to only 0.1µg/ml Lat B caused no apparent changes to the overall embryonic body phenotype. Likewise, hepatoblast numbers in LatB- and DMSO-treated embryos are similar (DMSO: 111.25 cells, SEM 6.51; Lat B: 105.89 cells, SEM 6.53; *p=0.6*), suggesting that LatB treatment did not significantly effect cell proliferation, another cellular processes that requires functional actin dynamics.

Quantification and morphometric analysis of liver budding

Ventral views of confocal stacks from whole-mount antibody preparations were used to measure the length of the Prox1-domain along the anteroposterior axis as indicated in Figure S2U. The extent of leftward liver displacement was assessed by determining the distance of the centre of the Prox1-domain to the embryonic midline in confocal stacks of transverse sections from whole-mount antibody labellings as depicted in Figure S2V. For this, the first section was randomly chosen and subsequent

sections, every 6 μm, were analysed and the average determined for every embryo. The number of hepatoblasts was determined by automatic quantification of Prox1^{+} nuclei. All measurements were carried out with Volocity software (PerkinElmer).

2D-Quantification of cell shapes and orientation

Cellular characteristics were determined by measuring length and width of hepatoblasts in coronal or transverse sections of whole liver confocal stacks using Volocity software (PerkinElmer), followed by calculation of the length/width ratio; on average 4 sections per liver and minimum of 3 embryos (n=3). The first section was randomly chosen and subsequently sections every 6 microns were analysed. Statistical significance was determined by Student's t-test. Orientation of elongated hepatoblasts represents the angle of the longest axis of the cell with respect to the anteroposterior axis of the embryo.

3D-Quantification of cell protrusions

Hepatoblast protrusions were manually tracked in three dimensions (x, y and z) through consecutive sections of confocal stacks from whole-mount livers (coronal views) using Volocity software (PerkinElmer). Stacks consist of optical sections acquired at least every 2 μm. Flat protrusions with a diameter $\geq$ 1.5 μm were classified as lamellipodia-like, and ones with a smaller diameter as filopodia-like (regular and branched). The protrusion number was determined as absolute number per μm^2 cell surface. Clone perimeter and height were measured and used to calculate the cell surface. Statistical significance was determined by Student's t-test. The orientation-angle of hepatoblast extensions is displayed with respect to the anteroposterior axis of the embryo.

Statistical analysis

To determine the statistical significance of the data sets, unpaired two-tailed Student's *t*-test was used. $p<0.05$ was used as a criterion for statistical significance in all experiments. Error bars represent s.e.m..

Live-Imaging and analysis

24-26 hpf embryos were embedded in a drop of 0,4% low melt agarose containing 200 μg/ml Tricaine and placed between two coverslips. Space between the cover slips was filled with approx. 1,5 ml of Embryo medium containing 0,2 mM PTU and 200 μg/ml Tricaine and sealed with vacuum grease. 4D (xyzt) imaging was performed using 20x objectives on a Leica SP8 or Zeiss LSM780 confocal microscope. Images were collected every 7-28 minutes (mostly 21 minutes), depending on single or multi-position acquisition. During the imaging process, embryo and digestive system development was delayed without altering development, including the foregut organs (see movie S4). Combinations of the following transgenes and fluorescent labels were used for tracking liver and gut progenitors: *Tg(-0,5sox17:GFP)^{zf99}* marks the endoderm, and *TgBAC(prox1a:kalTA4-4xUAS:uncTagRFP)^{nim5}* labels predominantly hepatoblasts with a mosaic onset. 200pg of *tagBFP-nls* mRNA (Figures 1J-L, S1F-H, movie S1) were injected to mark all nuclei or 20pg of UAS:TagBFP-nls DNA co-injected with 20pg of *Tol2 transposase* mRNA to mark hepatoblast nuclei under the control of *prox1a:kalTA4* (movies S2, S4). Hepatoblast migration on a single cell level and including cellular protrusions was imaged in *TgBAC(prox1a:kalTA4-4xUAS:uncTagRFP)^{nim5};Tg(UAS:lyn-Citrine)^{nim23}* embryos (Figure 2E, movie S3). Sparse labeling is due to mosaic onset of *prox1a:kalTA4* and generally mosaic expression of *UAS:lyn-Citrine*, likely due to silencing. Datasets were reconstructed for subsequent analysis using Imaris BITPLANE software (version 8.1.2). The analysis included the following steps:

1) 3D Drift Correction. Samples often drift during image acquisition reducing the ability for accurately tracking and measuring cell movements over time and therefore need to be corrected for accurate tracking. The displacement of two successive data points over time was determined by normalized cross correlation of their maximum intensity projections. Correction of the whole time series was achieved by transforming each time frame with the cumulated displacements. Drift was corrected using 2-4 manually selected 3D landmarks with anatomical correspondence between all time frames using Imaris BITPLANE software.

2) Mask Generation: GFP signal from *Tg(-0,5sox17:GFP)^{zf99}* was used to create a mask of endoderm cells (Figure S1F,G).

3) Semi-automated Cell Tracking. Cells were tracked in drift-corrected datasets using nuclei marked by TagBFP-nls. An autoregressive motion algorithm was used for automatic cell tracking. Tracks were carefully analysed by at least two users and then corrected manually. Two criteria were used to discriminate between liver and gut progenitor cells: i) the position during liver bud outgrowth phase, which often corresponded to the end of the movie, and ii) *TgBAC(prox1a:kalTA4-4xUAS:uncTagRFP)^{nim5}* expression at the liver bud outgrowth phase; subsequent backtracking

confirmed cell identity. Position coordinates of every tracked cell for all time points were exported to an Excel file.

4) Generating displacement vectors for each time point. Steps 3 to 5 were carried out with MatLab R2016a software. Displacement vectors of a tracked cell were generated by subtracting X, Y and Z coordinates of two successive positions. Displacement of different cell types was determined with respect to a meaningful anatomical reference plane: the anteroposterior plane (A-P) corresponds to the embryonic midline defined at 24-26hpf, and the left-right (L-R) one is perpendicular to the A-P plane. The embryonic midline was defined using reconstructed 3D datasets. Three random points on each plane were selected to generate the corresponding plane equation in MatLab R2016a software. Later a normal vector to these planes was obtained by using point-normal form and general form of the equation of a plane in coordinate geometry.

5) Generating angle between displacement vector and reference plane. Vector geometry formulas i.e. cross product was used to determine the angle between the displacement vectors for each time frame (obtained in step 3) and the unit vector parallel to the A-P or L-R plane.

6) Representation of angular distributions. Rose plots were generated summarizing the angular distribution of displacement vectors with respect to A-P and L-R planes for all time frames. The range of angles is represented in the area of each wedge and the concentric circles represent the observed angle frequency within a particular range. The range of angles is represented by the size of each wedge (bin size), which was set to 15 degree. Line plots show orientation of the displacement vectors with respect to A-P and L-R planes for each time frame.

References:

Alexander, J., Rothenberg, M., Henry, G.L., and Stainier, D.Y. (1999). casanova plays an early and essential role in endoderm formation in zebrafish. Dev Biol *215*, 343-357.

Bushell, K.M., Sollner, C., Schuster-Boeckler, B., Bateman, A., and Wright, G.J. (2008). Large-scale screening for novel low-affinity extracellular protein interactions. Genome Res *18*, 622-630.

Distel, M., Wullimann, M.F., and Koster, R.W. (2009). Optimized Gal4 genetics for permanent gene expression mapping in zebrafish. Proc Natl Acad Sci U S A *106*, 13365-13370.

Dunworth, W. P., Cardona-Costa, J., Bozkulak, E. C., Kim, J.-D., Meadows, S., Fischer, J. C., et al. (2014). Bone morphogenetic protein 2 signaling negatively modulates lymphatic development in vertebrate embryos. *Circulation Research*, *114*(1), 56–66. http://doi.org/10.1161/CIRCRESAHA.114.302452

Haas, P., and Gilmour, D. (2006). Chemokine signaling mediates self-organizing tissue migration in the zebrafish lateral line. Dev Cell *10*, 673-680.

Ho, C.Y., Houart, C., Wilson, S.W., and Stainier, D.Y. (1999). A role for the extraembryonic yolk syncytial layer in patterning the zebrafish embryo suggested by properties of the hex gene. Curr Biol *9*, 1131-1134.

Kanca, O., Caussinus, E., Denes, A.S., Percival-Smith, A., and Affolter, M. (2014). Raeppli: a whole-tissue labeling tool for live imaging of Drosophila development. Development *141*, 472-480.

Korzh, S., Emelyanov, A., and Korzh, V. (2001). Developmental analysis of ceruloplasmin gene and liver formation in zebrafish. Mech Dev *103*, 137-139.

Köster, R.W., and Fraser, S.E. (2001). Tracing transgene expression in living zebrafish embryos. Dev Biol *233*, 329-346.

Kurita, R., Sagara, H., Aoki, Y., Link, B.A., Arai, K., and Watanabe, S. (2003). Suppression of lens growth by alphaA-crystallin promoter-driven expression of diphtheria toxin results in disruption of retinal cell organization in zebrafish. Dev Biol *255*, 113-127.

Mizoguchi, T., Verkade, H., Heath, J.K., Kuroiwa, A., and Kikuchi, Y. (2008). Sdf1/Cxcr4 signaling controls the dorsal migration of endodermal cells during zebrafish gastrulation. Development *135*, 2521-2529.

Mosimann, C., Kaufman, C.K., Li, P., Pugach, E.K., Tamplin, O.J., and Zon, L.I. (2011). Ubiquitous transgene expression and Cre-based recombination driven by the ubiquitin promoter in zebrafish. Development *138*, 169-177.

Morton, W. M., Ayscough, K. R., & McLaughlin, P. J. (2000). Latrunculin alters the actin-monomer subunit interface to prevent polymerization. *Nature Cell Biology*, *2*(6), 376–378. http://doi.org/10.1038/35014075

Phng, L.-K., Stanchi, F., & Gerhardt, H. (2013). Filopodia are dispensable for endothelial tip cell guidance. *Development*, *140*(19), 4031–4040.

Urasaki, A., Morvan, G., and Kawakami, K. (2006). Functional dissection of the Tol2 transposable element identified the minimal cis-sequence and a highly repetitive sequence in the subterminal region essential for transposition. Genetics *174*, 639-649.

Warming, S., Costantino, N., Court, D.L., Jenkins, N.A., and Copeland, N.G. (2005). Simple and highly efficient BAC recombineering using galK selection. Nucleic Acids Res *33*, e36.

Westerfield, M. (2000). The Zebrafish Book. A Guide for the Laboratory Use of Zebrafish (Danio rerio), 4th edn edn (Eugene, OR: University of Oregon Press).
